# Natural short-lived halogens exert an indirect cooling effect on climate

**DOI:** 10.1038/s41586-023-06119-z

**Published:** 2023-06-28

**Authors:** Alfonso Saiz-Lopez, Rafael P. Fernandez, Qinyi Li, Carlos A. Cuevas, Xiao Fu, Douglas E. Kinnison, Simone Tilmes, Anoop S. Mahajan, Juan Carlos Gómez Martín, Fernando Iglesias-Suarez, Ryan Hossaini, John M. C. Plane, Gunnar Myhre, Jean-François Lamarque

**Affiliations:** 1 Department of Atmospheric Chemistry and Climate, Institute of Physical Chemistry Rocasolano, CSIC, Madrid, Spain; 2 Institute for Interdisciplinary Science (ICB), National Research Council (CONICET), FCEN-UNCuyo, Mendoza, Argentina; 3 Institute of Environment and Ecology, Tsinghua Shenzhen International Graduate School, Tsinghua University, Shenzhen, China; 4 Atmospheric Chemistry Observations and Modeling Laboratory, National Center for Atmospheric Research, Boulder, CO USA; 5 Centre for Climate Change Research, Indian Institute of Tropical Meteorology, Ministry of Earth Sciences, Pune, India; 6 Instituto de Astrofísica de Andalucía, CSIC, Granada, Spain; 7 Deutsches Zentrum für Luft- und Raumfahrt (DLR), Institut für Physik der Atmosphäre, Oberpfaffenhofen, Germany; 8 Lancaster Environment Centre, Lancaster University, Lancaster, UK; 9 School of Chemistry, University of Leeds, Leeds, UK; 10 CICERO Center for International Climate Research, Oslo, Norway; 11 Climate and Global Dynamics Laboratory, National Center for Atmospheric Research, Boulder, CO USA; 12 Present Address: Department of Civil and Environmental Engineering, The Hong Kong Polytechnic University, Hong Kong, China

**Keywords:** Atmospheric chemistry, Climate and Earth system modelling

## Abstract

Observational evidence shows the ubiquitous presence of ocean-emitted short-lived halogens in the global atmosphere^1–3^. Natural emissions of these chemical compounds have been anthropogenically amplified since pre-industrial times^4–6^, while, in addition, anthropogenic short-lived halocarbons are currently being emitted to the atmosphere^7,8^. Despite their widespread distribution in the atmosphere, the combined impact of these species on Earth’s radiative balance remains unknown. Here we show that short-lived halogens exert a substantial indirect cooling effect at present (−0.13 ± 0.03 watts per square metre) that arises from halogen-mediated radiative perturbations of ozone (−0.24 ± 0.02 watts per square metre), compensated by those from methane (+0.09 ± 0.01 watts per square metre), aerosols (+0.03 ± 0.01 watts per square metre) and stratospheric water vapour (+0.011 ± 0.001 watts per square metre). Importantly, this substantial cooling effect has increased since 1750 by −0.05 ± 0.03 watts per square metre (61 per cent), driven by the anthropogenic amplification of natural halogen emissions, and is projected to change further (18–31 per cent by 2100) depending on climate warming projections and socioeconomic development. We conclude that the indirect radiative effect due to short-lived halogens should now be incorporated into climate models to provide a more realistic natural baseline of Earth’s climate system.

## Abstract (web summary)

Short-lived halogens have a substantial indirect cooling effect on climate and this cooling effect has increased since pre-industrial times owing to anthropogenic amplification of natural halogen emissions.

## Main

The climate significance of ocean–land–atmosphere gas exchange has primarily focused on the partitioning of greenhouse gases (for example, carbon dioxide (CO_2_), methane (CH_4_) and nitrous oxide (N_2_O)) and the release of biologically produced dimethyl sulfide (DMS), which forms aerosols through secondary oxidation reactions^9^. Less attention has been paid to natural sources of other reactive gases that, through altering the atmospheric oxidation capacity, have the potential to impact Earth’s radiative balance and climate indirectly. One such group of reactive gases is the so-called short-lived halogen species (SLH; chlorine, bromine and iodine compounds with a lifetime of less than six months in the atmosphere). For the past two decades, observational evidence collected from around the world has shown the ubiquitous presence of SLH in the global atmosphere^10–19^. These species are naturally emitted from the oceans, polar ice and the biosphere^1,2^, presenting a variable spatio-temporal source strength that is expected to increase owing to climate change^20^. In addition, a recent rapid increase in anthropogenic emissions of chlorinated SLH has been identified in the atmosphere^7,8,21^.

The breakdown of SLH in the atmosphere yields highly reactive halogen radicals that play important roles in several atmospheric processes, including the depletion of ozone (O_3_) through catalytic cycles, direct CH_4_ chemical loss, alteration of the hydroxyl (OH) radical, and the hydrogen (HO_2_/OH) and nitrogen (NO_2_/NO) oxides balance^2^ (see reactions R1–R17 in Supplementary Table 1). Halogens also oxidize oceanic DMS, influencing the formation of cloud condensation nuclei^22^, and in the case of iodine, higher iodine oxides and oxoacids have been shown to condense spontaneously to form ultrafine aerosols^23–25^. Combined, this large and growing body of research has demonstrated that natural plus anthropogenic SLH can exert a profound impact on the chemistry and composition of the atmosphere on a global scale. However, their effect on Earth’s radiative balance remains almost unexplored.

Since their initial implementation in global chemistry–climate models^26,27^, the emissions and chemistry of SLH have revealed that they have the potential to substantially alter the oxidizing capacity of the atmosphere^28–31^, both over pristine and polluted environments. Atmospheric oxidation in turn determines the abundance of short-lived climate forcers (SLCF) such as CH_4_, tropospheric O_3_ and aerosols, which are key contributors to climate warming^32–36^. In particular, SLH constitute a natural buffer to anthropogenic O_3_ pollution owing to a negative feedback mechanism that regulates natural emissions of iodine^37,38^, as well as modulating CH_4_ lifetime via direct and indirect chemical loss processes^39^. In addition, SLH affect the evolution of O_3_ in the climate-relevant lower stratosphere^21,40^. So far, climate models used in international climate assessments, such as the Coupled Model Intercomparison Project Phase 6 (CMIP6)^41^ and the different assessment reports of the Intergovernmental Panel on Climate Change (IPCC)^32,35^, have not included the sources and chemistry of SLH. Here we use a state-of-the-art Earth-system model to quantify the contribution of SLH to the global energy balance across pre-industrial, present-day and future climates. Our results show that natural SLH exert an indirect cooling effect on the climate system and that this natural cooling effect has been amplified since pre-industrial times owing to anthropogenic activity.

## Net radiative effect

We use the Community Earth System Model (CESM; see ‘CESM (CAM-Chem) model configuration and experiments design’ in Methods) to quantify the influence of SLH on the global radiative balance (see Extended Data Table 1 for modelling cases). The radiative effect (RE) caused by halogen radicals on the main SLCF, namely O_3_, CH_4_, aerosols and stratospheric water vapour are computed for past, present and future climate scenarios (‘The RRTMG radiation module in CESM’ in Methods). Sources of SLH are grouped into three categories: natural (NAT), anthropogenically amplified natural emissions (AANE) and anthropogenic (ANT). For pre-industrial simulations (year 1750), we consider only natural sources, mainly emitted from the oceans (for example, hypoiodous acid (HOI) and bromoform (CHBr_3_)) and polar regions (for example, bromine monochloride (BrCl) and molecular chlorine (Cl_2_)) by biogenic and photochemical processes (NAT). For present-day (2020) and future (2100) simulations, anthropogenic pollution has important impacts on global SLH emissions, including (Extended Data Table 2): (1) the direct emissions of inorganic (for example, hydrogen chloride (HCl)) and organic (for example, dichloromethane (CH_2_Cl_2_)) SLH from anthropogenic activities (ANT; for example, industrial, coal burning, waste burning and so on); and (2) anthropogenic emissions of primary pollutants (for example, nitrogen oxides and volatile organic compounds from transport, shipping, industry, power plants and so on), which subsequently form secondary air pollutants (for example, O_3_ and nitric acid) that drive the anthropogenic amplification of natural SLH emissions (AANE; Methods).

**Fig. 1:**
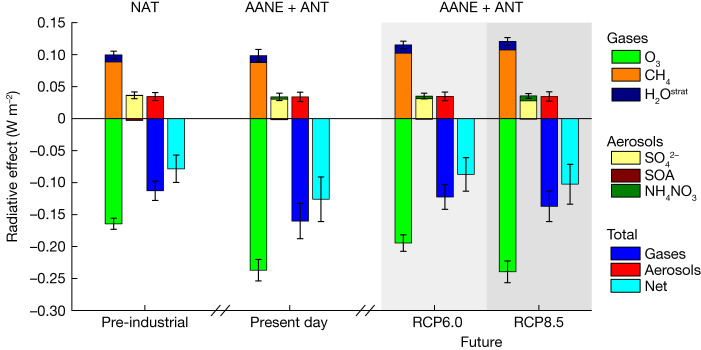
Radiative effect of SLH on gas and aerosol SLCF. RE for all-sky conditions at the top of the model owing to natural halogens in the pre-industrial (left) together with anthropogenic plus anthropogenically amplified natural emissions (AANE + ANT) in the present day (centre). The RE owing to AANE + ANT halogens in year 2100 for RCP6.0 (light-grey shading) and RCP8.5 (dark-grey shading) climate scenarios are also shown on the right. The individual contributions from different SLCF are grouped into short-lived gases (O_3_, CH_4_ and stratospheric water vapour (H_2_O^strat^)) and aerosols (mainly sulfate, SOA and NH_4_NO_3_). The halogen-mediated radiative contribution from all gases (resulting in net cooling) and aerosols (producing net warming), as well as the net (gas + aerosol) is shown for each period. The uncertainty range for each species is calculated as half of the difference between the maximum and minimum RE obtained for the complete set of model sensitivities for each individual time period (mean ± range/2) as described in Supplementary Information and Extended Data Table 5. A comparison between only AANE and AANE + ANT cases for all-sky and clear-sky conditions during different time periods is shown in Extended Data Fig. 1.

**Extended Data Fig. 1:**
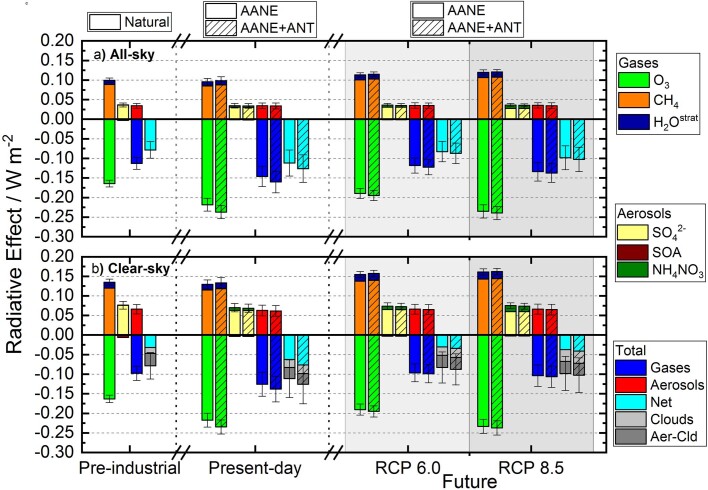
Radiative effect of SLH on short-lived climate forcers at the top of model. RE for the only AANE and AANE+ANT configurations are shown by empty and black-striped colored bars, respectively, for all-sky (**a**) and clear-sky (**b**) conditions. Results for the pre-industrial period are on the left and consider only natural halogen emissions, while the RE in year 2100 for RCP6.0 (light-grey shading) and RCP8.5 (heavy-grey shading) climate scenarios are shown on the right. The RE due to clouds and aerosol-cloud (Aer-Cld) interaction is shown on top of the net (gas+aerosol) effect on **b**. Comparison of only AANE and AANE+ANT results indicates that most of the RE due to SLH arise from the contribution of natural sources that have been anthropogenically amplified during present-day and end-of-the-century conditions. The uncertainty range for each species is computed as half of the difference between the maximum and minimum RE obtained for the complete set of model sensitivities for each individual time period (mean ± range/2) as described in the Supplementary Information (see Extended Data Table 5).

Figure 1 shows that present-day natural and anthropogenic SLH exert a net (gas + aerosols) indirect cooling effect of −0.13 ± 0.03 W m^−2^ for all-sky conditions (see Extended Data Fig. 1 for results distinguishing clear-sky, clouds and aerosol–cloud contributions). This value is the result of the distinct halogen-mediated radiative impact on O_3_ (−0.24 ± 0.02 W m^−2^), CH_4_ (+0.09 ± 0.01 W m^−2^), aerosols (+0.03 ± 0.01 W m^−2^) and stratospheric water vapour (+0.011 ± 0.001 W m^−2^). A comprehensive analysis of model uncertainty and results dependence on SLH burden is provided in Supplementary Information and summarized in Extended Data Table 3. We now detail the influence of SLH on each of the main chemically active SLCF.

### Ozone

Halogen radicals efficiently destroy atmospheric O_3_ through catalytic cycles^1^. Global models currently estimate that halogens reduce the tropospheric O_3_ burden by about 10−20% (refs. ^29,30,38^), resulting in a net cooling effect of approximately −0.1 W m^−2^ (refs. ^27,28,42^). Inclusion of halogens in our Earth-system model, for pre-industrial conditions, results in a global-mean decrease in tropospheric and stratospheric O_3_ of −3.3 Dobson units (DU) and −3.9 DU, respectively (Extended Data Table 4), leading to a total reduction in the O_3_ RE of −0.16 ± 0.01 W m^−2^ (Extended Data Table 5). The corresponding changes in present-day tropospheric and stratospheric O_3_ are −4.9 DU and −5.2 DU, respectively, which induce a net RE reduction of −0.24 ± 0.02 W m^−2^. By the end of the century, projected O_3_ RE is −0.19 ± 0.01 W m^−2^ (total O_3_ loss of −8.5 DU) for the representative concentration pathway 6.0 (RCP6.0) scenario and −0.24 ± 0.02 W m^−2^ (−10.7 DU) for RCP8.5.

### Methane

Tropospheric O_3_ is the principal source of OH, the main atmospheric oxidant and the dominant chemical sink of CH_4_, which is the second-largest greenhouse gas after CO_2_ (ref. ^33^). Our results show that SLH increase the global CH_4_ burden by +14% and +9% for pre-industrial and present-day conditions, respectively, resulting in an RE enhancement of +0.09 ± 0.01 W m^−2^ during both time periods. The greater burden and RE of CH_4_ associated with SLH is the result of the indirect halogen-driven decrease in CH_4_ oxidation by OH outweighing the direct increase in CH_4_ loss by chlorine atoms^39^. By 2100, halogen-induced CH_4_ RE is +0.10 ± 0.01 W m^−2^ for RCP6.0 and +0.11 ± 0.01 W m^−2^ for RCP8.5, resulting from burden increases of 464 Tg (11%) and 936 Tg (7%), respectively, compared with the corresponding future scenario omitting SLH (Extended Data Table 4).

### Stratospheric water vapour

In the troposphere, water vapour is regulated by the local environment (for example, temperature, dew point and so on). However, in a predominantly dehydrated stratosphere, the chemical oxidation of CH_4_ influences the stratospheric water vapour abundance. The chemistry of CH_4_ in the lower stratosphere is similar to that in the troposphere, with OH radicals oxidizing CH_4_ in the same manner (reaction R16 in Supplementary Table 1). As described above, SLH increase the CH_4_ burden and thus stratospheric water vapour, leading to a warming RE in the stratosphere of +0.011 ± 0.001 W m^−2^ (Extended Data Table 5). The relative contribution of halogen-driven water vapour RE in the future stratosphere is similar to that at present (Fig. 1).

### Aerosols

The aforementioned halogen impacts on atmospheric oxidants (OH radicals, O_3_, chlorine and so on) also lead to changes in the formation of secondary aerosols (aerosols formed following the oxidation of directly emitted gaseous precursors), including sulfate ${{\rm{SO}}}_{4}^{2-}$, secondary organic aerosols (SOA) and ammonium nitrate (NH_4_NO_3_; see reactions R18–R21 in Supplementary Table 1)^43^. It is noted that all these aerosol species present a dominant cooling effect in the troposphere owing to the reflection of solar incoming shortwave radiation, and the inclusion of SLH results in a reduction of this cooling effect by decreasing aerosol formation on the global scale. The estimated impact of halogens on aerosol RE reaches +0.03 ± 0.01 W m^−2^ for both pre-industrial and present-day conditions (see Methods for the contribution of individual aerosol species). Although a recent focus of research^43–45^, it is noted that large uncertainties still remain on the contribution of halogens to the global secondary aerosol loading.

In summary, natural changes in atmospheric composition mediated by SLH during pre-industrial times lead to a significant reduction in O_3_ RE (−0.16 ± 0.01 W m^−2^), a relatively small increase in stratospheric water vapour RE (+0.011 ± 0.001 W m^−2^), a noticeable enhancement in the CH_4_ RE (+0.09 ± 0.01 W m^−2^) and a slight increase in the RE from aerosols (+0.03 ± 0.01 W m^−2^; mostly due to sulfate reduction; Fig. 1 and Extended Data Table 5). The net pre-industrial RE is estimated to be −0.08 ± 0.02 W m^−2^, with a dominant contribution from gaseous species (−0.11 ± 0.02 W m^−2^). In comparison, the SLH-driven reduction in net RE is stronger at present (−0.13 W m^−2^ versus −0.08 W m^−2^) because: (1) the inorganic halogen tropospheric burden is larger than in pre-industrial (147–187% for Cl_*y*_, 8–9% for Br_*y*_ and 24–29% for I_*y*_; Extended Data Table 3); and (2) both CH_4_ (about 150%) and tropospheric O_3_ (about 40%) burdens have also increased since pre-industrial times owing to anthropogenic activity (Extended Data Table 4). The present-day greater abundance of tropospheric reactive halogens largely responds to the anthropogenic amplification of natural emissions (AANE) over the oceans, which dominates the change in halogen sources and burden over the direct continental emissions of anthropogenic halogens (ANT; Extended Data Table 2).

## Spatial distribution of radiative effect

**Fig. 2:**
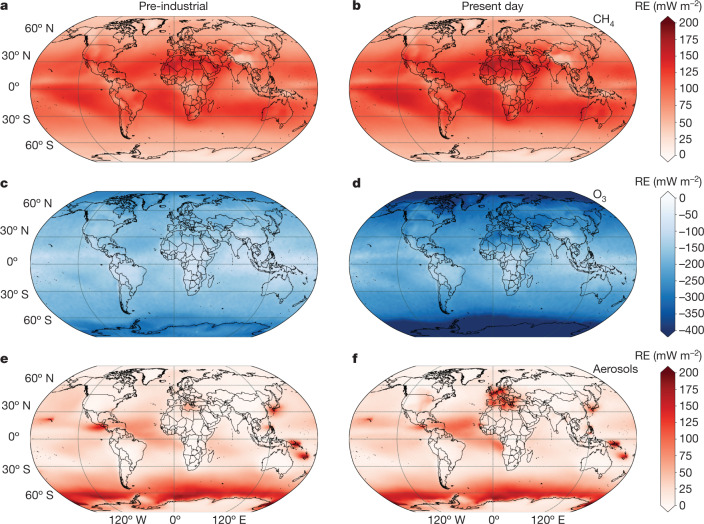
Spatially resolved SLH-driven RE of the main SLCF. **a**–**f**, The individual RE contribution arising from CH_4_ (**a**,**b**), O_3_ (**c**,**d**) and aerosols (**e**,**f**) at the top of the model are shown for the natural emission simulation during pre-industrial times (NAT; left) and the anthropogenic plus anthropogenically amplified natural emissions in the present day (AANE + ANT; right). It is noted that the CH_4_ RE reaches a maximum within the low latitudes resulting in net heating, whereas the O_3_ radiative cooling is more prominent over the high latitudes. The aerosol RE reaches a maximum over the Southern Ocean owing to the OH reduction caused by SLH, presenting spatial hotspots over industrialized regions such as Europe, North America and East Asia during the present day. The spatially resolved RE for the RCP6.0 and RCP8.5 scenarios is shown in Extended Data Fig. 2 and the radiative contribution for individual aerosol species is shown in Extended Data Fig. 4. All maps and elements were created by our research group using Matplotlib Basemap for Python.

**Fig. 3:**
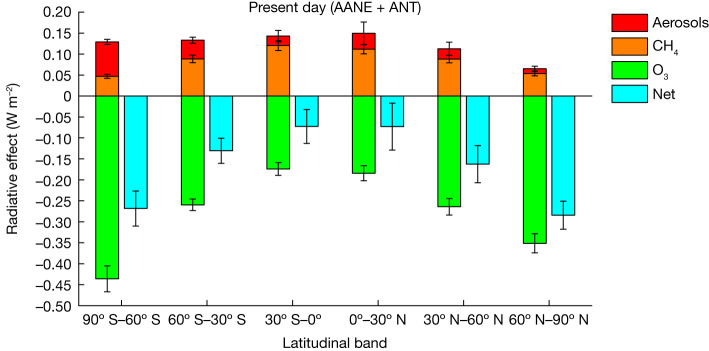
Latitudinal variation of SLH-induced RE on SLCF under present-day conditions. Despite the opposite sign of the RE induced by SLH on CH_4_ (positive orange bars, warming) and O_3_ (negative green bars, cooling), changes in CH_4_ RE peak at low latitudes and close to the Equator, whereas the O_3_ RE reaches a maximum over the high latitudes and polar regions. The SLH-mediated RE contribution from aerosols peaks over the southern high latitudes and shows the largest uncertainty over the northern mid-latitudes. Consequently, the net (gas + aerosols, cyan bars) perturbation of SLH on the radiative balance shows a pronounced latitudinal variation, where the net high-latitude RE can be up to three-times larger than within the low latitudes. The uncertainty range for each species is calculated as half of the difference between the maximum and minimum RE obtained for the complete set of present-day model sensitivities (mean ± range/2) as described in Supplementary Information (see also Extended Data Table 5).

**Extended Data Fig. 2:**
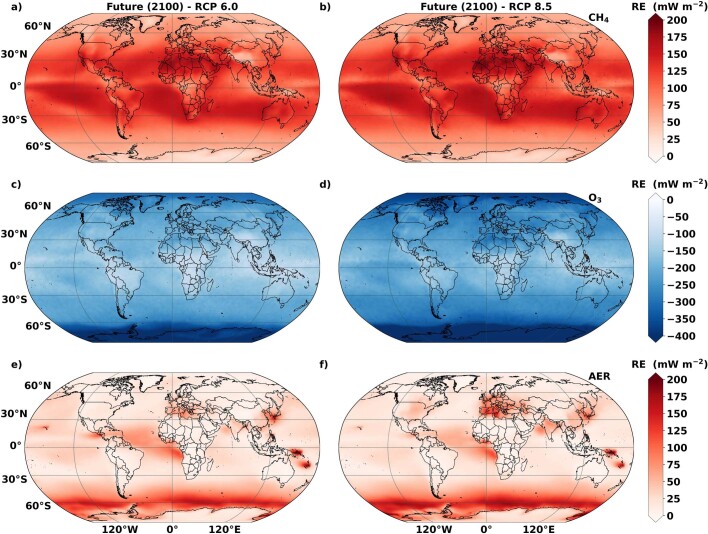
Spatially-resolved SLH-driven radiative effect of the main short-lived climate forcers during future scenarios. The individual RE contribution arising from methane (**a,b**), ozone (**c,d**) and aerosols (**e,f**) at the top of the model are shown for the AANE+ANT configuration in year 2100 for the RCP 6.0 (left column) and RCP 8.5 (right column) scenarios. Equivalent panels for pre-industrial and present-day conditions are shown in Fig. 2. All maps and elements were created by our research group using Matplotlib Basemap for Phyton.

SLH are emitted from various sources around the globe with large spatial heterogeneity. The dominant natural sources arise from the ocean whereas the main anthropogenic sources are located over continental regions (Supplementary Information). Figure 2 shows that the SLH-mediated RE during the pre-industrial and the present day is most noticeable over the open ocean and polar regions where natural halogens are emitted by seawater, sea-salt aerosols, first-year sea-ice and blowing snow (see Extended Data Fig. 2 for future scenarios). The large SLH-driven RE within high latitudes is mainly due to both tropospheric and stratospheric O_3_ changes, with a much smaller contribution from CH_4_; whereas over the low latitudes, opposite contributions from O_3_ and CH_4_ almost cancel out (Fig. 3). Indeed, SLH have been shown to increase the depth and size of the stratospheric ozone hole over Antarctica during austral spring^40,46^, which further enhances the cooling effect of halogens in the lower stratosphere over the Southern Hemisphere during the present day compared with the pre-industrial scenario (see ‘Additional aspects of SLH influence on SLCF’ in Methods). Hence, the cooling effect of SLH peaks at high latitudes for all climate scenarios, that is, within the Earth regions that are predicted to be most affected by global warming^47,48^.

**Extended Data Fig. 3:**
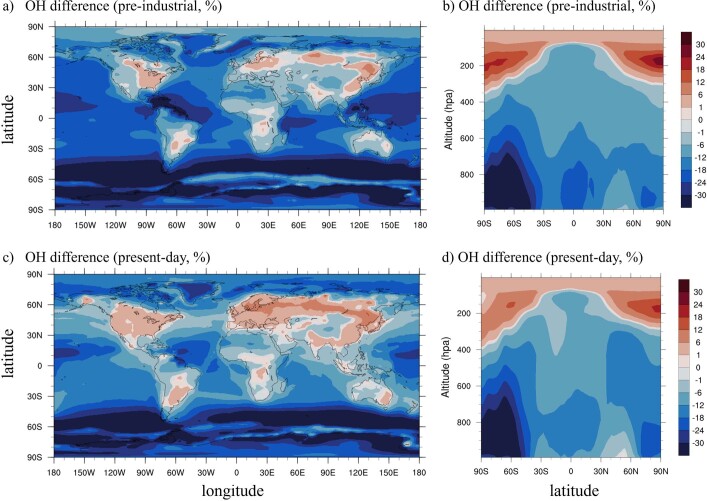
SLH-driven perturbation of the atmospheric oxidative capacity. The percentage change in hydroxyl radical (OH) mixing ratios for pre-industrial (**a,b**) and present-day (**c,d**) are computed as PI(%) = (Natural−NoSLH)/NoSLH × 100% and PD(%) = (AANE+ANT−NoSLH)/NoSLH × 100%, respectively (see definitions of model simulations in Extended Data Table 1). The annual mean surface OH difference is shown in the left column panels, whereas the annual zonal mean difference is presented on the right panels. Note that even though SLH increase surface OH abundance over the continents (mostly above industrialized regions during present-day), the net effect of SLH on the oxidative capacity is to reduce the global mean OH abundance. All maps and elements were created by our research group using gsn_csm library for NCL.

**Extended Data Fig. 4:**
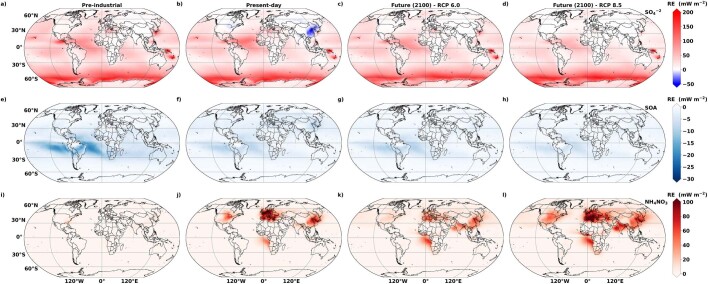
Spatially-resolved radiative effect contribution for individual aerosols. The individual contribution arising from sulfate (**a,b,c,d**; top row), SOA (**e,f,g,h**; middle row) and ammonium nitrate (**i,j,k,l**; bottom row) for the AANE+ANT configuration are shown for pre-industrial (1^st^ column; only NAT), present-day (2^nd^ column), RCP 6.0 (3^rd^ column) and RCP 8.5 (4^th^ column) scenarios. The total contribution for all aerosols together is shown in the bottom row of Fig. 2 and Extended Data Fig. 2. The aerosol RE present a pronounced spatial variability (most notorious for ammonium nitrate, which has a predominant anthropogenic origin), with maximum impacts during present-day over industrialized regions such as Europe, North America and East Asia. The halogen-driven RE of sulfate aerosols during present-day (panel **b**) changes from positive to negative over China, in agreement with ref. ^43^. All maps and elements were created by our research group using Matplotlib Basemap for Phyton.

SLH also lead to a reduction in aerosol formation and a subsequent warming on the global scale, mostly driven by the reduction in tropospheric OH abundance caused by halogens (Extended Data Fig. 3). Regional changes in present-day aerosol RE are found over industrialized and urban regions such as Europe, the east coast of North America and East Asia, where SLH coexist with high levels of air pollutants (Extended Data Fig. 4). This includes highly localized cooling effects over China (Extended Data Fig. 4b), which are consistent with the SLH-driven enhancement in aerosol haze pollution^43^ (see ‘Additional aspects of SLH influence on SLCF’ in Methods).

## Change relative to pre-industrial times

**Fig. 4:**
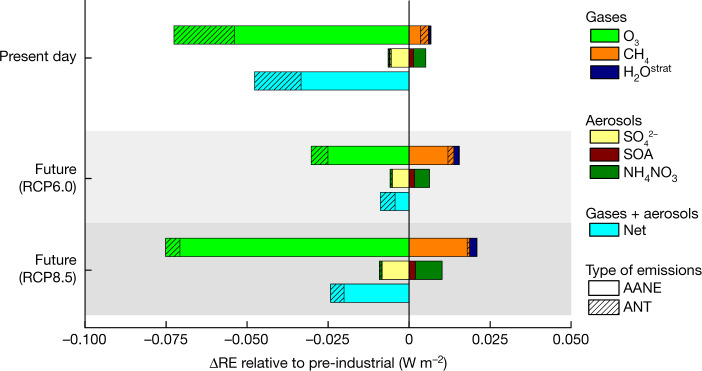
Change in SLH-driven RE on SLCF with respect to pre-industrial times. The change in radiative effect (ΔRE) for different periods of time distinguishes the contribution from pure anthropogenic halogen emissions (ANT, black-striped coloured bars) with respect to the anthropogenic amplification of natural SLH emissions (AANE, empty coloured bars). The contribution of ANT is largest during present times and, regardless of the scenario considered, the contribution of AANE increases in the future. Compared with present times, the SLH-driven ΔRE for CH_4_ is projected to increase (warming) by the end of the century regardless of the emissions scenario considered; whereas, for O_3_, the strength of the cooling effect (negative ΔRE) depends on the future RCP scenario considered. Future RCP results are based on time-slice simulations representative of the year 2100.

We now quantify the present and future changes in the RE of active SLCF relative to the pre-industrial climate (ΔRE; see ‘The RRTMG radiation module in CESM’ in Methods), evaluating the contribution and time evolution of AANE compared with ANT. Figure 4 shows that the combined ΔRE due to SLH is −0.05 ± 0.03 W m^−2^ at present, of which about 30% is due to ANT and about 70% is due to AANE. Changes in ΔRE are more uncertain towards the future: the SLH-mediated ΔRE by 2100 is −0.01 ± 0.03 W m^−2^ for RCP6.0 (about 51% for ANT and about 49% for AANE) and −0.02 ± 0.03 W m^−2^ for RCP8.5 (about 17% for ANT and about 83% for AANE). It is noted that the SLH-mediated radiative changes from the pre-industrial to present and future are mostly driven by AANE, that is, natural halogen emissions that are amplified by anthropogenic perturbations (Extended Data Fig. 1). Thus, the increase in the SLH indirect cooling effect since pre-industrial times is not a result of direct anthropogenic emissions but instead indirectly arises from the amplification of natural halogen emissions owing to human activities, and the subsequent effects of these emissions on various SLCF. The main driver of AANE is the anthropogenic increase in O_3_ pollution and its subsequent deposition onto the ocean surface^38,49^ that has amplified, by a factor of two to three, the oceanic emission of iodine since the mid-twentieth century, as evidenced by measurements in Arctic and Alpine ice cores and tree rings^4–6^. The presence of anthropogenic air pollutants (for example, strong acids) also affects the partitioning of reactive halogen species and their heterogeneous recycling on sea-salt aerosols and blowing snow, which perturbs the release of gaseous bromine and chlorine to the atmosphere^2^.

**Extended Data Fig. 5:**
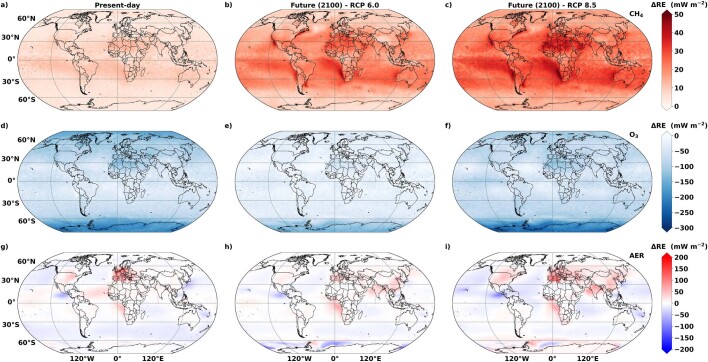
Spatial distribution of the SLH-driven change in radiative effect (ΔRE) for individual SLCF. ΔRE for methane (**a,b,c**; top row), ozone (**d,e,f**; middle row) and aerosols (**g,h,i**; bottom row) with respect to the pre-industrial period are shown for the AANE+ANT simulations. Note that methane presents a pronounced enhancement for both RCP 6.0 and RCP 8.5, while for the case of ozone ΔRE for present-day and future RCP 8.5 scenarios is larger than for the future RCP 6.0 because the latter presents more stringent air-pollutants reductions. ΔRE for aerosols shows a pronounced spatial-distribution, presenting positive and negative variations due to the different contributions of sulfate, ammonium nitrate and SOA (see Extended Data Fig. 4). All maps and elements were created by our research group using Matplotlib Basemap for Phyton.

The breakdown of ΔRE shows that the relative contributions of individual SLCF are of opposite signs and compensate each other to result in a net cooling effect (Fig. 4 and Extended Data Fig. 5). For instance, AANE dominates ΔRE for CH_4_ at the end of the century regardless of the scenario considered, which responds to the anthropogenic amplification of iodine and bromine emissions from the global oceans^38^, which significantly reduce the levels of OH radicals and in turn CH_4_ oxidation^39^. In contrast, during the present day, the relative contributions of AANE and ANT to CH_4_ ΔRE are comparable (Fig. 4). It is noted that most of the ΔREdriven by CH_4_ occurs in the lower troposphere, whereas for O_3_, a significant ΔRE contribution also occurs in the lower stratosphere, where in addition to the natural SLH changes, the rapid increase in the anthropogenic emissions of short-lived chlorocarbons also contributes to O_3_ depletion^21,28^ (Extended Data Table 4). Thus, ΔRE for O_3_ from the pre-industrial to the present has a significant contribution from pure anthropogenic sources (about 26% ANT compared with about 74% AANE), whereas it is projected to reduce to about 17% ANT (about 83% AANE) for RCP6.0 and about 6% ANT (about 94% AANE) for RCP8.5 by the end of the century. Similarly, the present-day ΔRE for CH_4_ relative to the pre-industrial period is attributed to about 42% ANT and about 58% AANE, whereas by the end of the century AANE dominates the signal (about 87% AANE under RCP6.0 and about 96% AANE for RCP8.5). The changing radiative effect of SLH across pre-industrial, present-day and future climates highlights the complex nonlinear chemical interaction between SLH and the abundance of key chemically active SLCF.

## Radiative influence of SLH on climate

**Fig. 5:**
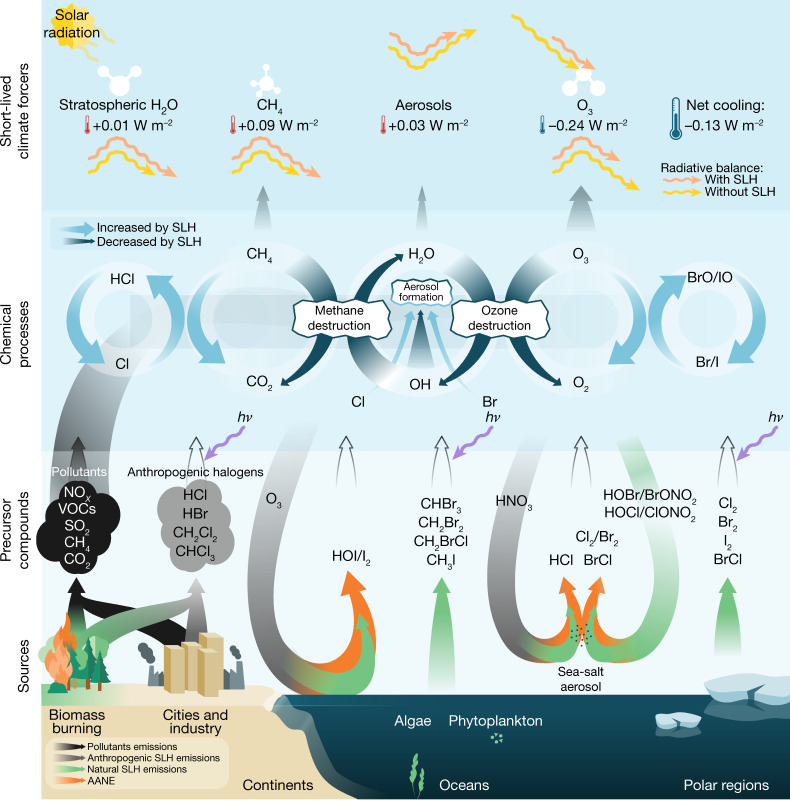
Conceptual representation of the SLH influence on atmospheric composition and radiative feedbacks within the climate system. Halogens influence the climate system through direct changes in O_3_ and OH radical chemical cycling, which in turn regulate the abundance of radiatively active SLCF such as CH_4_, aerosols and stratospheric water vapour (H_2_O). The widening (thinning) of the semi-circular arrows within the chemical process layer represents an enhancement (reduction) of the efficiency of the direct SLH-driven (light blue) and indirect OH-driven (dark blue) chemical recycling of CH_4_, H_2_O and O_3_. The green, grey and black upwards arrows within the precursor’s layer are the direct emissions of natural SLH, anthropogenic SLH and anthropogenic air pollutants, respectively. The U-shaped arrows show natural atmospheric cycling processes of halogenated (greenish tail) and anthropogenic (greyish tail) chemical reservoirs, respectively, both of which have been anthropogenically amplified (orange head) and altered the baseline state of the climate system. The length variation of the curly yellow and pink arrows on the uppermost SLCF layer represents the effect induced by SLH on Earth’s radiative balance. The individual warming and cooling effect of each individual SLCF, as well as the net SLH-driven cooling RE, are synthesized as coloured thermometers. Figure 5 was created by NorArte Visual Science (https://www.norarte.es/en/) upon request.

SLH are naturally emitted from the oceans, ice and aerosol surfaces, as well as from the biosphere and anthropogenic activities. Their natural emissions are strongly linked to climate (for example, sea surface temperature, primary productivity, lifting of sea-salt aerosols by winds and sea-ice extent) and to anthropogenic pollution (O_3_ deposition to the ocean and atmospheric acidification)^37,39^. In addition, anthropogenic SLH not controlled by the Montreal Protocol have shown a rapid increase over East Asia and other developing regions during the past decade^7,8,21^. This changing role of SLH in controlling the oxidizing capacity of the troposphere and, consequently, in regulating the abundance of radiative-active SLCF, together with the anthropogenic amplification of the natural SLH emissions (AANE), affects the baseline radiative budget of the atmosphere in different ways (Fig. 5). Therefore, past and future changes in halogen emissions, and their indirect effect on Earth’s radiative balance through altering the oxidative capacity, are determined by a combination of natural and anthropogenic emissions, climate variability and atmospheric chemistry.

The addition of present-day anthropogenic halogen emissions on top of AANE induces a slight change in global net RE (−0.11 ± 0.03 W m^−2^ for only AANE and −0.13 ± 0.03 W m^−2^ for AANE + ANT). Indeed, the relative contribution of AANE to the total halogen effect further increases in the future, regardless of considering a mid- or high-emissions scenario (Fig. 4 and Extended Data Table 5). This highlights that amplified natural halogen sources (AANE), which cannot be directly controlled by environmental agreements but whose emissions depend on the emissions of anthropogenic pollutants that can be regulated, dominate the global SLH effect on the climate system. The analysis demonstrates that the SLH-driven RE is a persistent and significant signal during all time periods, with variable uncertainties dominated by the predicted levels of tropospheric halogens within each scenario.

The halogen impacts on RE have a marked geographical distribution. Noticeably, given the larger RE influence of SLH at high latitudes (Fig. 3), the inclusion of SLH is expected to alter the atmospheric heat redistribution from the equatorial regions to the high latitudes, that is, maximizing the cooling effect of halogens over the polar regions, which are expected to suffer the largest temperature enhancements owing to global warming^47,48^.

Finally, we highlight that the net indirect cooling effect caused by SLH is the result of a trade-off between the spatially variable effects of halogens mainly on O_3_ (both tropospheric and stratospheric) and CH_4_, with a minor contribution from aerosols and stratospheric water vapour. This so far unrecognized interplay between natural SLH and Earth’s radiative balance is nonlinear across pre-industrial, present-day and future climates. Models that do not include this indirect RE may overestimate the warming induced by SLCF since pre-industrial times. Furthermore, our results show that the net cooling effect of halogens has been amplified since pre-industrial times owing to the linkage between halogen emissions and atmospheric pollutants, and this complex interplay is expected to further change depending on future climate projections. The forcing caused by SLH over the industrial era (−0.05 W m^−2^) is similar to that produced by the increase of dust emissions (−0.07 W m^−2^)^50^ and of equivalent magnitude but opposite sign as the combined contrail and contrail-induced cirrus forcing (0.06 W m^−2^)^32^. We conclude that SLH are a key component of the natural climate system as they exert an indirect cooling effect currently not accounted for in climate model assessments and, therefore, we suggest the need to include a complete representation of natural and anthropogenic SLH in climate models to reduce uncertainties in the contribution of SLCF to the evolution of Earth’s radiative balance from pre-industrial to future climates.

## Methods

### CESM (CAM-Chem) model configuration and experiments design

The Community Earth System Model (CESM) version 1.1.1 (ref. ^51^), including the Community Atmospheric Model with interactive chemistry (CAM-Chem) version 4.0 (ref. ^52^), was used to quantify the overall impact of SLH on Earth’s energy balance from pre-industrial times to the end of the twenty-first century. The model was configured with a horizontal resolution of 1.9° latitude × 2.5° longitude (96 × 144 grid points, respectively) and 26 vertical levels that extend from the surface to approximately 40 km (3.5 hPa in the upper stratosphere), following a hybrid sigma pressure coordinate^53^.

The standard chemical scheme implemented in CAM-Chem includes 169 species with both gas-phase and heterogeneous reactions coupled to the radiation module^54^. Updates for the chemical processing of SLH include a state-of-the-art chemical mechanism for halogens in the troposphere and the stratosphere, which has been described in detail in previous studies. Briefly, ref. ^26^ presented the implementation of reactive halogen species sources and chemistry in CAM-Chem, including a comprehensive validation of halocarbon source gases using available observations. References ^30,38,55,56^ then further updated the halogen CAM-Chem set-up to include a more detailed representation of chlorine, bromine and iodine gas- and heterogeneous-phase chemistry, which allowed to quantify the influence of SLH on stratospheric O_3_ (ref. ^40^). A polar module, including inorganic halogen sea-ice emissions from the Arctic and Antarctica^57^, as well as the impact of halogens on CH_4_ lifetime and burden^39^, have also been implemented into the current SLH version of CAM-Chem. Furthermore, here we have implemented and improved a few additional model developments: (1) the OH/O_3_/NO_3_-initiated SOA production yield was updated^58^; (2) chlorine- and bromine-induced formation of SOA was considered^43^; we added (3) the HOBr + S reaction to form sulfate aerosol following refs. ^59,60^, as well as (4) the heterogeneous recycling of bromine species on anthropogenic aerosols^43,61^; and we also included (5) a consistent representation of iodine-containing particle formation from higher iodine oxides^25,62^ and (6) the injection of gas-phase and particulate iodine to the stratosphere^13,46^. The main reactions of relevance for this work are summarized in Supplementary Table 1; for a full set of halogen reactions implemented in CAM-Chem, see the supplementary material in ref. ^63^.

Natural SLH sources in CESM (CAM-Chem) include both biogenic and abiotic pathways (Fig. 5). Biogenic sources comprise nine halocarbons (CHBr_3_, CH_2_Br_2_, CH_2_BrCl, CHBr_2_Cl, CHBrCl_2_, CH_3_I, CH_2_I_2_, CH_2_IBr and CH_2_ICl), which are the result of micro- and macro-algae as well as phytoplankton metabolism coupled to photochemistry at the ocean’s surface^26^. The evolution of these SLH biogenic emissions is treated in a consistent framework in which they are coupled to physical and biogeochemical changes (for example, sea surface temperature, marine primary production and so on) related to climate and atmospheric composition^37^. Abiotic sources have distinct routes for chlorine and bromine compared with iodine. Chlorine and bromine are released from sea-salt aerosols following acid displacement (for example, induced by HNO_3_) as well as heterogeneous reactions of nitrogenated (for example, N_2_O_5_), halogenated (for example, HOBr, HOCl and HOI) and halo-nitrogenated (for example, BrONO_2_, ClONO_2_ and IONO_2_) reservoirs, constituting the dominant sources of reactive bromine and chlorine in the lower troposphere^55,64–66^. Inorganic iodine (HOI and I_2_), however, is directly emitted from the ocean surface following O_3_ deposition on seawater and its reactions with aqueous iodide^38,49,67^. Emissions of bimolecular inter-halogen species (that is, Cl_2_, Br_2_ and I_2_, as well as BrCl, IBr and ICl) from the sea-ice surface within the Arctic and Antarctica are also computed online^57^ (Fig. 5).

Anthropogenic SLH sources are included following an emission inventory of the two dominant organic chlorine species (CH_2_Cl_2_ and C_2_Cl_4_) (ref. ^68^), complemented by lower boundary conditions of other anthropogenic chlorinated substances (CHCl_3_, C_2_H_4_Cl_2_ and C_2_HCl_3_) (refs. ^39,64,69^). In this study, we further implemented an anthropogenic global emission inventory of reactive inorganic halogen species for the year 2014 (applied to present-day conditions), including inorganic chlorine (HCl and fine particle chloride) from coal burning, biomass burning and waste burning, as well as inorganic bromine (HBr and Br_2_) and iodine (HI and I_2_) from coal burning (see further details in ‘Emission inventory of global anthropogenic inorganic halogens’). Extended Data Table 2 and Supplementary Figs. 1–3 show the contribution of natural and anthropogenic emissions to the atmospheric halogen budget, and Extended Data Table 3 summarizes the surface mixing ratios and tropospheric burden for total inorganic chlorine (Cl_*y*_), bromine (Br_*y*_) and iodine (I_*y*_) for the natural (AANE) and full (AANE + ANT) simulations during the pre-industrial, the present-day and the end of the century. Supplementary Figs. 5–7 show the geographical and vertical distributions of Cl_*y*_, Br_*y*_ and I_*y*_.

The standard CESM (CAM-Chem) anthropogenic pollutant emissions developed for the Chemistry–Climate Intercomparison Project (CCMI)^70^ have been used here following ref. ^51^. These include anthropogenic and biomass burning emissions from the Monitoring Atmospheric Composition and Climate/CityZen inventory (MAC City) with an annual resolution until the year 2010 (ref. ^71^), merged with IPCC Fifth Assessment Report emissions afterwards^72^. CAM-Chem was configured with the bulk aerosol model, which simulates the distribution of externally mixed sulfate, black carbon, primary organic carbon, sea salt and dust, as described in ref. ^52^. Aircraft emissions of black carbon and nitrogen dioxide, as well as volcanic emissions of sulfur and sulfate, are vertically distributed. The set-up also includes an emissions-driven approach for CH_4_ instead of applying the standard lower boundary surface mixing ratios for long-lived species. The main CH_4_ sources include agriculture, landfill, fossil fuel industry, biomass and biofuel burning, and natural emissions from wetlands (see ref. ^39^ for further details). Biogenic emissions are calculated online within the land module using the Model of Emissions of Gases and Aerosols from Nature (MEGAN) version 2.1 (ref. ^73^).

The model set-up is based on specified dynamic time-slice simulations considering three distinct periods: pre-industrial times, representative of the year 1750; present-day conditions for the year 2020; and future conditions at the end of the century (year 2100) for two different projected scenarios (see below). Time-slice simulations for each period comprise 15-year integrations driven by nudging every 3 h a varying meteorology (temperature, winds and surface pressure) from a previous simulation that omitted the contribution of SLH^30,55,56^. It is noted that even though the meteorology was obtained considering mean climatological boundary conditions representative of 2000–2020, pre-industrial, present-day and future sensitivities considered sea surface temperature and sea-ice conditions representative of each time period^37^. All experiments were initialized from a previous simulation after allowing 40 years of spin-up to ensure all chemical species, particularly CH_4_, were stabilized. Beyond historical periods, future projections are based on the mid- and high-end RCP (RCP6.0 and RCP8.5, respectively) emissions scenarios^74,75^ for both long-lived species and short-lived precursor emissions. Long-lived halogen-containing species (CH_3_Cl, CH_3_CCl_3_, CCl_4_, CFC-11, CFC-12, CFC-113, HCFC-22, CFC-114, CFC-115, HCFC-141b, HCFC-142b, CH_3_Br, H-1301, H-1211, H-1202 and H-2402) lower boundary conditions follow the A1 halogen scenario from the Scientific Assessment on Ozone Depletion (SAOD-2010) report^76^.

Benchmark model simulations for all time periods are split into three categories with distinctive treatment of SLH (Extended Data Table 1): (1) NoSLH: standard chemical scheme without SLH sources and chemistry; (2) NAT/AANE: only natural SLH emissions scenario (NAT for pre-industrial; AANE for present-day and future scenarios where the online computation of natural SLH emissions have been anthropogenically amplified); and (3) AANE + ANT: anthropogenically amplified natural emissions plus anthropogenic SLH sources for the present day and future. It is noted that anthropogenic and biogenic emissions other than SLH are identical within the NoSLH, AANE and AANE + ANT scenarios. Given that no anthropogenic SLH emissions are considered for pre-industrial runs, NAT represents pristine background halogen conditions, whereas AANE represents perturbed halogen conditions owing to anthropogenic air pollutants affecting the SLH natural source strength, particularly via the abiotic route of O_3_ deposition on the ocean surface, the acid enhancement of sea-salt recycling and the biotic route of SLH emissions due to changes in climate (Fig. 5 and Extended Data Table 2)^38,39,77^. The difference in the radiation budget between AANE and NoSLH represents the RE driven by the natural amplification of the halogen burden owing to the background levels of pollutants during a fixed period of time; whereas the difference between AANE + ANT and NoSLH represents the RE of all reactive halogen species.

### The RRTMG radiation module in CESM

In this work, we distinguish between the terms radiative effect (RE) and the change in radiative effect (ΔRE): RE (Fig. 1) is the change in the radiative balance between a simulation considering SLH with respect to a baseline simulation omitting SLH, both during the same time period; whereas ΔRE (Fig. 4) is the change in RE between different time periods (for example, between present day and pre-industrial times). RE and ΔRE were computed using the Rapid Radiative Transfer Model for Global circulation models (RRTMG) package^78^, which is currently the default radiative transfer scheme included in CESM v2 (ref. ^79^). The RRTMG radiation module provides an online diagnostic tool to quantify and distinguish the downwards and upwards as well as shortwave and longwave radiation at various layers, including the surface and top of the model^80,81^. In particular, RRTMG allows splitting the individual radiative contribution for independent radiatively active constituents, which can be added or subtracted one by one to or from the complete radiative components list (for example, considering the single-addition and single-subtraction contribution of each species to the total radiative budget; see ref. ^80^). Radiative magnitudes shown in this work were obtained considering the 15-year global mean for each individual benchmark configurations and the 5-year mean for the complete set of sensitivities described in Supplementary Information. The RE uncertainty associated with each independent simulation represents the interannual variability computed as two times the standard deviation (2*σ*) of the multi-year global average.

Here we use the RRTMG diagnosis variables FSNT (net solar flux at top of model) and FLNT (net longwave flux at top of model) for all-sky conditions, as well as their equivalent streaming for clear-sky conditions (FSNTC and FLNTC, respectively). Individual values of all magnitudes were obtained for the following list of radiatively active climate forcers (CESM name-list variables included in parenthesis): (1) gases: water vapour (H_2_O), carbon dioxide (CO_2_), nitrous oxide (N_2_O), ozone (O_3_), methane (CH_4_) and chlorofluorocarbons (CFC12 and CFC11STAR, which includes the contribution from CFC11 plus other minor CFCs and HCFCs); and (2) aerosols: sulfate (SO_4_), dust (DST01-04), black carbon (CB1 and CB2), organic carbon (OC1 and OC2), secondary organic aerosols (SOAM, SOAI, SOAT, SOAB and SOAX), ammonium nitrate (NH_4_NO_3_) sea salt (SSLT01-04) and iodine particles (IOP). The RE for individual gas- and aerosol-phase species, as well as that resulting from the sum of all gases, aerosols and the net (gas + aerosols) effect of each radiatively active species (represented by *S*) for each emission sensitivity case (*C*; NAT, AANE, AANE + ANT) and period of time (*T*; pre-industrial, present day, and future RCP6.0 and RCP8.5), were computed as follows:

1 $${\rm{RE}}{(S)}_{T}^{C}={[{\rm{FSNT}}(S)-{\rm{FLNT}}(S)]}_{T}^{C}-{[{\rm{FSNT}}(S)-{\rm{FLNT}}(S)]}_{T}^{{\rm{NoSLH}}}$$

The change in the RE for a given time period with respect to pre-industrial times is computed as follows (see equations (2) and (3) below). First, the RE for the AANE and AANE + ANT scenarios for each period of time (for example, respectively defined as ${\rm{RE}}{(S)}_{{\rm{PD}}}^{{\rm{AANE}}}$ and ${\rm{RE}}{(S)}_{{\rm{PD}}}^{{\rm{AANE+ANT}}}$ for present-day (PD) conditions) is computed relative to the NoSLH scenario. For the case of pre-industrial (PI), only the natural RE $({\rm{RE}}{(S)}_{{\rm{PI}}}^{{\rm{NAT}}})$ is considered. Second, we compute the change in RE during the present day $(\Delta {\rm{RE}}{(S)}_{{\rm{PD}}-{\rm{PI}}}^{C})$, always with respect to the RE obtained for the pre-industrial, and split the natural (AANE) with respect to the anthropogenic (ANT) contributions as follows:

2 $$\Delta {\rm{RE}}{(S)}_{{\rm{PD-PI}}}^{{\rm{AANE}}}={\rm{RE}}{(S)}_{{\rm{PD}}}^{{\rm{AANE}}}-{\rm{RE}}{(S)}_{{\rm{PI}}}^{{\rm{NAT}}}$$

3 $$\Delta {\rm{RE}}{(S)}_{{\rm{PD-PI}}}^{{\rm{ANT}}}={\rm{RE}}{(S)}_{{\rm{PD}}}^{{\rm{AANE+ANT}}}-\Delta {\rm{RE}}{(S)}_{{\rm{PD-PI}}}^{{\rm{AANE}}}$$

An equivalent procedure was applied to compute the change in RE by the end of the century for the RCP6.0 $(\Delta {\rm{RE}}{(S)}_{{\rm{RCP6.0-PI}}}^{C})$ and RCP8.5 $(\Delta {\rm{RE}}{(S)}_{{\rm{RCP8.5-PI}}}^{C})$ scenarios relative to pre-industrial times.

It is noted that owing to the superposition of absorption bands of the different radiatively active species, the sum of the individual RE contribution of each species slightly differs from the net RE of all species combined^82^. Indeed, this difference depends on the consideration of a single-addition or single-subtraction analysis in the radiative computation, and can result in a non-zero RE contribution from non-reactive gases such as CO_2_. To minimize these overlapping differences, we computed the normalized RE for all species considering the 0.428 (single addition) and 0.572 (single subtraction) weighting factors provided in ref. ^80^, whereas for aerosols only, single-subtraction magnitudes were considered. The small nonlinearity on the radiative assignation of the net RE to individual SLCF (that is, −0.03 W m^−2^) is attributed to neglecting the contribution of rapid adjustments (that is, radiation-driven changes in land surface and tropospheric temperatures) as well as to the different longwave absorption of overlapping bands when individual species are added to or subtracted from the radiation name-list. For the particular case of stratospheric water vapour, we computed its RE as 12.5% of the CH_4_ RE (that is, in the middle of the various estimates compiled in the IPCC Sixth Assessment Report)^32^.

### Emissions inventory of global anthropogenic inorganic halogens

In this study, we further develop a global emissions inventory of reactive inorganic halogen species for the year 2014 (applied to present-day conditions), including inorganic chlorine (HCl and fine particle chloride) from coal burning, biomass burning and waste burning, as well as inorganic bromine (HBr and Br_2_) and iodine (HI and I_2_) from coal burning. Source strength estimates of these inorganic halogen sources are zeroed for pre-industrial conditions and scaled into the future based on the RCP6.0 or RCP8.5 evolution of anthropogenic sulfur dioxide and carbon monoxide from biomass burning^39^.

Within our global inorganic halogen inventory, country-level emissions are calculated using the emissions factor method, following the methodology used in previous studies^43,83^. Briefly, for activity data, country-level coal consumption from power plants, industry and residential burning are obtained from the International Energy Agency (www.iea.org) database. Dry matter burned from forest, grassland, peat and agriculture waste are derived from the Global Fire Emission Database (www.globalfiredata.org). Waste burned in incineration plants or by open burning are obtained from official statistics or calculated based on ref. ^84^. For China, detailed local and county-level activity data are used. Emissions factors of gas-phase halogen species arising from coal burning are calculated based on halogen content in coal and removal efficiencies of air pollution control devices. The halogen content in coal is obtained from our previous studies^43,83^, the United States Geological Survey database and other measurements^85,86^. The installation rates of different air pollution control devices are from the Tsinghua emissions database^87–89^ for China and from the PKU-FUEL database for other regions (inventory.pku.edu.cn). Other parameters, such as release rates, removal efficiencies and other emissions factors, are described in detail in our previous studies^43,83^. The proportions of emitted inorganic halogen species were set as 70% and 30% for HBr and Br_2_, and 95% and 5% for HI and I_2_, respectively^90,91^.

Supplementary Figs. 1–3 present the spatial distribution of anthropogenic halogen emissions in comparison with the oceanic natural SLH emissions implemented in CAM-Chem^37^. Hotspots for continental chlorine emissions are located in China, India, Southeast Asia and Africa, with a peak emission intensity larger than 1.0 × 10^−13^ kg m^−2^ s^−1^. China and India are also the major emitters of anthropogenic bromine and iodine, with emission fluxes higher than 1.0 × 10^−13^ kg m^−2^ s^−1^ in polluted areas. The global mean source strength for natural (NAT), anthropogenic (ANT) and AANE is compared in Extended Data Table 2.

### Additional aspects of SLH influence on SLCF

Given the current uncertainties on the SLH–aerosol interaction over both polluted and pristine environments^43^, the net aerosol RE induced by SLH sources and chemistry presents the largest relative errors of all the SLCF considered in this work (Supplementary Information). Sulfate dominates the net RE of aerosols, reaching +0.036 ± 0.005 W m^−2^ for the pre-industrial and +0.030 ± 0.006 W m^−2^ for present-day conditions. Even though the SLH-induced NH_4_NO_3_ RE is small at present (+0.004 ± 0.001 W m^−2^), this species showed a pre-industrial to present-day burden enhancement that is two times larger than that for sulfate (Extended Data Table 4). This means that during pre-industrial times, the larger halogen-driven changes in atmospheric oxidants affected mostly sulfate, which has a significant natural precursor. In contrast, during present-day and future scenarios, the SLH influence on NH_4_NO_3_ is larger because of its dominant anthropogenic precursors. Consequently, the cooling effect of both sulfate and NH_4_NO_3_ is weaker when natural halogens are considered. Regarding secondary organic aerosols, AANE drives a global reduction of their formation (owing to a less oxidative atmosphere), while localized ANT emissions of inorganic halogens over industrial regions can enhance secondary aerosol formation during haze pollution events^43^ (Extended Data Fig. 4).

In the end-of-the-century future projections, the net RE induced by SLH is weaker than in the present time regardless of the emissions scenario considered (RE = −0.09 ± 0.03 W m^−2^ for RCP6.0 and RE = −0.10 ± 0.03 W m^−2^ for RCP8.5; Fig. 1 and Extended Data Table 5). However, the independent contributions of the individual gases altering the net radiative balance differ: under RCP6.0 and owing to the more stringent restriction on air pollutant emissions, the global tropospheric O_3_ burden is reduced by the end of the century^37^, and consequently the SLH influence on O_3_ RE is significantly weaker (RE = −0.19 ± 0.01 W m^−2^) compared with the present. In contrast, owing to the future increase in global CH_4_ burden, its warming RE owing to SLH slightly increases with respect to the present (RE = +0.10 ± 0.01 W m^−2^). Under RCP8.5, the enhancement in tropospheric O_3_ burden results in a similar net RE as in the present day (RE = −0.24 ± 0.02 W m^−2^), which is offset by the larger increase in CH_4_ emissions projected under RCP8.5, resulting in a halogen-driven RE warming of +0.11 ± 0.01 W m^−2^ for CH_4_. This dichotomy in the opposite contribution of halogen-mediated O_3_ and CH_4_ REs under different climate scenarios (Fig. 4) highlights the nonlinear chemical interaction between SLH and SLCF^33^.

Regarding the spatial heterogeneity of the RE, it is noted that the SLH-mediated changes in atmospheric composition depend significantly on the chlorine, bromine and iodine distribution over both oceanic and continental domains (Supplementary Figs. 5–7), which in turn shift the nonlinear atmospheric chemistry response in different ways (Extended Data Fig. 3). For instance, emissions of SLH in clean environments (for example, oceanic and polar) tend to reduce tropospheric O_3_, thereby leading to a reduction in atmospheric oxidation capacity (see reactions R3–R7 in Supplementary Table 1); whereas in polluted environments (for example, urban and industrial) SLH emissions can result in tropospheric O_3_ formation^92^, which in turn enhances the oxidizing capacity (that is, increase in OH) on regional scales (see reactions R9–R15 in Supplementary Table 1). Consequently, during present-day and future simulations, where SLH coexist with high levels of air pollutants, the change in RE due to SLH over continental regions is more pronounced compared with pre-industrial times (Extended Data Fig. 4). Therefore, future research focused on the spatial and seasonal variability of the SLH-mediated RE is needed to improve our understanding of the evolution of the baseline Earth’s radiative budget.

The SLH influence on O_3_ is the highest in the lowermost stratosphere, presenting a pronounced latitudinal dependence^93^ that increases towards the high latitudes, altering the O_3_ budget exactly in the region where surface temperature and climate are most sensitive to O_3_ perturbations^94^. Despite the well known influence of SLH on the Antarctic ozone hole^76^, approximately half of the additional stratospheric O_3_ destruction driven by short-lived bromine over Antarctica during the present time corresponds to a baseline O_3_ destruction on the global stratosphere^40^, a feature observed in our simulations during all time periods. This background additional O_3_ destruction owing to SLH is larger at high latitudes compared with the low latitudes (Fig. 3) and is also observed for sensitivity simulations where polar halogen sea-ice emissions are turned off (Supplementary Information). In addition, as the efficiency of the natural bromine and iodine background on stratospheric O_3_ depletion peaks during late spring and summer^40,46^, significant SLH-driven stratospheric O_3_ cooling is observed also by the end of the century over the Arctic and Antarctica regardless of the continuous reduction of anthropogenic long-lived O_3_-depleting substances (Fig. 2 and Extended Data Fig. 2). It is noted that our model configuration does not consider the dynamical feedbacks of stratospheric O_3_ that have been shown to influence surface temperature and precipitation over the southern tip of South America^95^. Both chemical (production and mostly loss) and transport (stratosphere-to-troposphere exchange) processes are altered when SLH are included^63^, although a distinction of each independent contribution is outside the scope of this work. Finally, it is noted that the radiative changes driven by stratospheric water vapour are only due to the chemical contribution from CH_4_ photochemistry in the stratosphere but, as all simulations were forced with the same meteorology, the results presented here do not account for the changes in tropopause temperature and/or dynamical features affecting the climate evolution of stratosphere–troposphere exchange.

### Evaluation of CESM (CAM-Chem) performance

We have conducted our simulations on the basis of previous studies (see ‘CESM (CAM-Chem) model configuration and experiments design’), which validated the modelled abundance of tropospheric reactive halogens and other relevant species (O_3_, OH and CH_4_) using a comprehensive set of ground-, ship-, aircraft- and satellite-based observations over the past 20 years. Briefly, ref. ^26^ performed an evaluation of the natural oceanic sources of short-lived halocarbons in CESM (CAM-Chem) against a large dataset of near-surface and aircraft campaigns in extra-polar regions, and ref. ^27^ included additional observations of reactive SLH and O_3_ over the tropics. Reference ^55^ showed the model ability to reproduce the bromine transport from the surface to the stratosphere, and refs. ^40^ and ^96^ reported an improvement in the total O_3_ column and ozone hole area over the Antarctic region, and in representing the observed vertical distribution of O_3_ mixing ratio in tropical regions, respectively. Reference ^77^ showed that the inclusion of natural SLH bromine and chlorine in CESM resulted in more realistic stratospheric inorganic halogen levels, improving the agreement with the SBUV-MOD (Solar Backscatter Ultraviolet merged total O_3_ column) dataset^97,98^. The implementation of iodine chemistry in CESM (CAM-Chem)^30^ allowed reproducing aircraft observations in the tropical upper tropopause^56^, suggesting the occurrence of iodine-driven stratospheric O_3_ depletion, which was later confirmed by ref. ^13^. In addition, refs. ^19,38^ demonstrated the need to consider an inorganic iodine source from the ocean surface to accurately reproduce the observed iodine oxide mixing ratios over the open ocean. The supplementary information of ref. ^37^ summarizes the performance of the SLH version of CESM in all previous modelling versus observational studies. Afterwards, ref. ^93^ evaluated the injection of inorganic and organic bromine to the stratosphere when chemical schemes with different degrees of complexity are considered, and ref. ^46^ reported the improved model performance of stratospheric O_3_ by including reactive iodine chemistry into the CESM/WACCM4-SD configuration. Furthermore, ref. ^99^ demonstrated satisfactory modelling of O_3_, as well as for the global sea-salt aerosol abundance in the marine boundary layer compared with global observational results; ref. ^39^ validated the modelled CH_4_, OH and reactive chlorine species against previous reports. To summarize, CESM (CAM-Chem) has consistently been able to provide reasonable estimates of the key ingredients relevant to our study, including reactive halogen species, sea-salt aerosols, tropospheric and stratospheric O_3_, tropospheric OH and global CH_4_ for the present day.

Here we provide further evaluation of CESM results for CH_4_ and O_3_ in pre-industrial and present-day simulations. The previous reported level of CH_4_ in the pre-industrial era is about 722 ppbv (parts per billion by volume)^35,100^, ranging from 697 ppbv over Antarctica to 759 ppbv over the Arctic based on ice-core observations^101^. Our simulated global average CH_4_ for the pre-industrial NAT case is at similar levels (722 ppbv global, 703 ppbv for Antarctica and 745 ppbv for the Arctic), suggesting that our model set-up properly represents the pre-industrial CH_4_ abundance. Reports on pre-industrial O_3_ are sparse. Supplementary Table 10 summarizes the available observation reports of O_3_ in the pre-industrial periods^102^. The average O_3_ mixing ratio at various sites is about 10 ppbv, ranging from 6.2 ppbv to 14.4 ppbv. Our modelling results at the same locations as observations for pre-industrial conditions averaged to be about 20 ppbv in the NoSLH case (between 16.2 ppbv and 24.1 ppbv) and about 15 ppbv in the NAT case (between 11.5 ppbv and 18.9 ppbv). Although the NAT case still overestimates the uncertain pre-industrial data, a robust feature of our simulations is that the model bias is significantly reduced compared with the NoSLH case (Supplementary Table 10), which supports and highlights the importance of considering SLH in climate models to improve the representation of pre-industrial O_3_ abundance. It is worth noting that most current climate models tend to overestimate the low surface O_3_ concentrations compared with these rather uncertain semi-quantitative observations performed during the late nineteenth century^102^.

For the present-day CH_4_ evaluation, we used the surface monthly average CH_4_ mixing ratio observations for the period 2000–2019 from the National Oceanic and Atmospheric Administration (NOAA) network^103^, which show a global average mixing ratio of 1,848 ppbv. Our CESM present-day results for both NoSLH and AANE + ANT represent reasonably well the global CH_4_, with global mean surface mixing ratios of 1,683 ppbv and 1,836 ppbv, respectively, considering the same grid points where the observations were made (Supplementary Fig. 10). Noticeably, SLH increased the simulated CH_4_ surface mixing ratio by 153 ppbv (or about 9%), compared with the NoSLH case within these sampling sites, highlighting that the inclusion of SLH brings the simulated CH_4_ levels closer to the NOAA observations. We used the monthly average surface O_3_ data from 2000 to 2015 in the Tropospheric Ozone Assessment Report (TOAR) dataset^104^ (https://toar-data.org/) to evaluate our present-day O_3_ CESM (CAM-Chem) results for the different model configurations. Supplementary Fig. 11 shows that both the NoSLH and AANE + ANT cases reproduce the global mean and range of observed surface O_3_ concentrations, although with some overestimation. The global O_3_ average of the TOAR dataset reaches 27.7 ppbv, whereas that for NoSLH is 41.1 ppbv and that for AANE + ANT is 33.8 ppbv, suggesting that the inclusion of SLH results in a more realistic representation of global surface O_3_. It is noted that even though the NoSLH configuration of CESM (CAM-Chem) tends to overestimate present-day surface O_3_, our modelled tropospheric O_3_ burden is on the lower edge of the group of chemistry–climate models participating in the CMIP6 activity^41,63^. However, our model configuration including SLH sources and chemistry reduces the high model bias of CMIP6 models^41^, and therefore results in a closer agreement with satellite- and ozonesonde-derived products relevant to global atmospheric chemistry model evaluation^63,105^.

To determine a robust estimation of the main uncertainties of the radiative and climatic influence of SLH, we performed a comprehensive sensitivity analysis where the emission strength, recycling efficiency and/or chemical reactivity of chlorine, bromine and iodine, as well as the background abundance of SLCF during each period of time, were varied within the range of values based on the most recent literature. This ‘standalone’ sensitivity analysis is described in Supplementary Information and relies on refs. ^107–113^. The analysis demonstrates that SLH induce a persistent and significant cooling signal during all time periods, with variable uncertainties dominated by the predicted levels of tropospheric halogens within each scenario. This SLH-driven RE is a robust signal for all scenarios considered, surpassing the estimated uncertainties related to the variable levels of tropospheric halogens and abundance of climate forcers for the different configurations. The uncertainty range for RE and ΔRE is computed considering the complete set of model sensitivities as described in Supplementary Information and summarized in Extended Data Table 5. Further research using other models and projected scenarios is required to shed light on the remaining unknowns related to the coupling between anthropogenic pollutant emissions and the SLH influence on the evolution of Earth’s radiative balance.

## Online content

Any methods, additional references, Nature Portfolio reporting summaries, source data, extended data, supplementary information, acknowledgements, peer review information; details of author contributions and competing interests; and statements of data and code availability are available at 10.1038/s41586-023-06119-z.

## Supplementary information

Supplementary Information This file contains Supplementary Text 1–4, Tables 1–10 and Figs. 1–11.

## Figures and Tables

**Extended Data Table 1:**
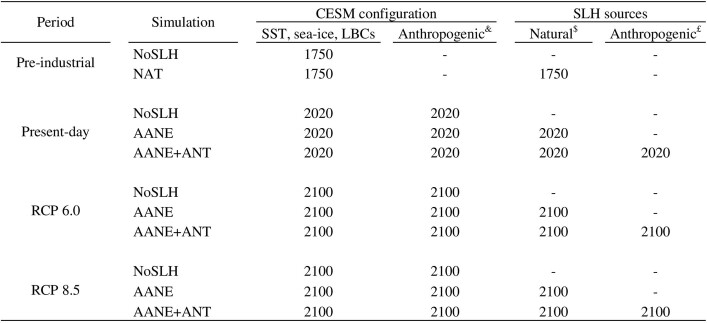
Design of CESM (CAM-Chem) benchmark model simulations

^&^The pre-industrial methane emission inventory used here^39^ results in a global mean surface CH_4_ mixing ratio of 722 ppbv for the pre-industrial NAT simulation. For present-day, cyclical methane emissions for year 2020 were used^39^. Long-lived gases lower boundary conditions (LBC) for pre-industrial times are from the Atmospheric Chemistry and Climate Model Intercomparison Project (ACCMIP)^70,106^, assuming that there was no emission change between 1750 and 1850. To obtain a realistic representation of the present-day stratospheric halogen burden, the long-lived LBCs were fixed to year 2020 for the SAOD-2010 scenario A1^76^. The main anthropogenic pollutants sources for year 2020 follow the Chemistry Climate Model Initiative (CCMI) recommendation for present-day conditions^51^ which are cycled during the 15-years integrations.

^$^Climate-induced changes in natural halocarbon fluxes follow the cli+bio sensitivity described in ref. ^37^, which includes both changes in physical (mainly due to sea-surface temperature (SST)) and biogeochemical (mostly related to marine primary production) factors. The short-lived halocarbon oceanic emissions during pre-industrial conditions are assumed to be identical to year 1850 (ref. ^39^). The sea-salt aerosol (SSA)-dehalogenation source for bromine and chlorine is based on ref. ^55^, with an updated scheme for HCl release due to the acidification of tropospheric aerosols^56^. Oceanic emissions of HOI/I_2_ are based on ref. ^38^, while sea-ice polar emissions follow ref. ^57^. All online inorganic halogen emissions are computed based on SST and sea-ice conditions representative of each period of time.

^£^Anthropogenic sources and LBCs of short-lived chlorocarbons during present-day conditions are based on refs. ^64^,^68^,^69^. For pre-industrial sensitivities anthropogenic SLHs were forced to zero, while for future simulations SLHs emissions and LBCs were assumed to linearly decrease down to zero after reaching their maximum values in year 2030 (ref. ^39^). The continental sources of inorganic halogens are described in ‘Emissions inventory of global anthropogenic halogens’ in Methods.

**Extended Data Table 2:**
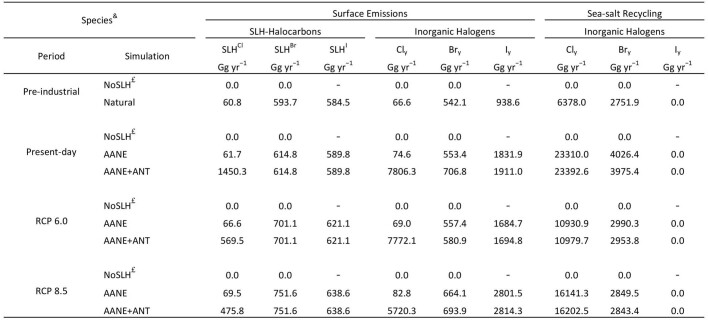
Natural, anthropogenic and anthropogenically amplified natural emissions of chlorine, bromine and iodine

^&^The global annual mean source strength for SLH is computed as follows: SLH^Cl^ = CHCl_3_ + CH_2_Cl_2_ + C_2_Cl_4_ + C_2_H_4_Cl_2_ + C_2_HCl_3_ + CH_2_BrCl + CH_2_ICl + CHBrCl_2_ + CHBr_2_Cl; SLH^Br^ = CHBr_3_ + CH_2_Br_2_ + CH_2_BrCl + CH_2_IBr + CHBrCl_2_ + CHBr_2_Cl; SLH^I^ = CH_3_I + CH_2_I_2_ + CH_2_ICl + CH_2_IBr; while the total inorganic halogen abundance is defined as: Cl_y_ = Cl + Cl_2_ + ClO + OClO + Cl_2_O_2_ + HCl + HOCl + ClONO_2_ + ClNO_2_ + BrCl + ICl; Br_y_ = Br + Br_2_ + BrO + HBr + HOBr + BrONO_2_ + BrNO_2_ + BrCl + IBr; I_y_ = I + I_2_ + IO + OIO + HI + HOI + IONO_2_ + INO_2_ + INO + IBr + ICl + I_2_O_2_ + I_2_O_3_ + I_2_O_4_. For total mass computations, only the halogen mass is considered, whereas for the sum of mixing ratios the halogen atomicity for each species is considered.

^£^Note that the NoSLH scheme does not include iodine chemistry but considers long-lived chlorine and bromine.

**Extended Data Table 3:**
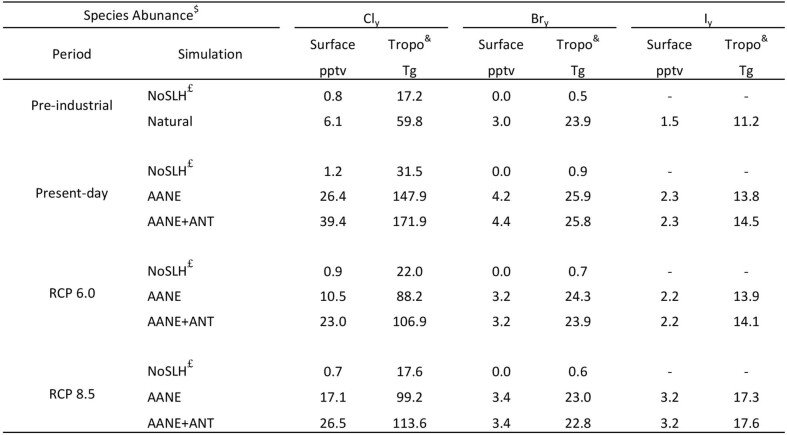
Global mean surface mixing ratio and tropospheric burden of inorganic halogens

^$^Total inorganic halogen abundance X_y_ (with X = Cl, Br, I) is defined in the footnote of Extended Data Table 2.

^&^Tropospheric burden (Tropo) has been computed considering the chemical tropopause (e.g O_3_ < 150 ppbv).

^£^Note that the NoSLH scheme does not include iodine chemistry but considers long-lived chlorine and bromine.

**Extended Data Table 4:**
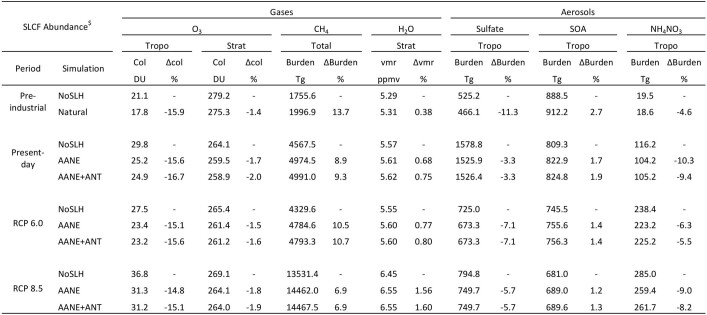
Global mean tropospheric and stratospheric abundance of main short-lived climate forcers

^$^Tropospheric (Tropo) and stratospheric (Strat) ozone columns (in Dobson units, DU), aerosol burdens (in Tg) and mean stratospheric water vapour abundance (in ppmv) have been computed considering the chemical tropopause (e.g O_3_ < 150 ppbv for Tropo and O_3_ ≥ 150 ppbv for Strat). For the case of methane, the total (Tropo+Strat) global burden (in Tg) is shown.

^&^The percentage change in the abundance of the different species during each time period has been computed between either AANE or AANE+ANT cases with respect to the NoSLH case.

**Extended Data Table 5:**
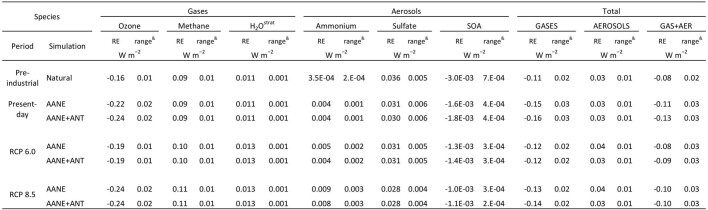
SLH-driven radiative effect for the main gas-phase and aerosol short-lived climate forcers

^&^Mean RE values are expressed as the 15-years average for each of the Natural, AANE and AANE+ANT configurations during a specific time period, while the uncertainty is computed as half of the difference between the maximum and minimum RE obtained for a complete set of model sensitivities (expressed as mean ± range/2) during each period of time (see Supplementary Information for further details). The uncertainty range for the total gases, aerosols and gases+aerosols columns are computed as the sum of the individual uncertainty of all species in each group.

## Data Availability

The data supporting this article, including the SLH chemical mechanism, model configuration files and post-processing scripts, are available at Mendeley Data (10.17632/gb7695c4vy.2). The complete dataset and routines used in this study are available from the corresponding author on reasonable request.

## Code availability

The benchmark CESM code is available from https://www.cesm.ucar.edu/models/releases. Administration of model updates related to SLH chemistry are maintained by NCAR engineers and will become available with the next CESM release.
