# Supplementary material for: Natural short-lived halogens exert an indirect cooling effect on climate
Source: Nature. 2023 Jun 28;618(7967):967–73. doi: 10.1038/s41586-023-06119-z (PMC10307623; doi:10.1038/s41586-023-06119-z)
Supplement: Supplementary file 1 — This file contains Supplementary Text 1–4, Tables 1–10 and Figs. 1–11. [file 41586_2023_6119_MOESM1_ESM.pdf]

nature portfolio

https://doi.org/10.1038/s41586-023-06119-z

# Supplementary information

# Natural short-lived halogens exert an indirect cooling effect on climate

In the format provided by the authors and unedited

# Supplementary Information for
# Natural short-lived halogens exert an indirect cooling effect on climate

Alfonso Saiz-Lopez[1*#], Rafael P. Fernandez[1,2#], Qinyi Li[1,$], Carlos A. Cuevas[1], Xiao Fu[3], Douglas E. Kinnison[4], Simone Tilmes[4], Anoop S. Mahajan[5], Juan Carlos Gómez Martín[6], Fernando Iglesias-Suarez[7], Ryan Hossaini[8], John M. C. Plane[9], Gunnar Myhre[10] and Jean-François Lamarque[11]

[1]Department of Atmospheric Chemistry and Climate, Institute of Physical Chemistry Rocasolano, CSIC, Madrid 28006, Spain
[2]Institute for Interdisciplinary Science (ICB), National Research Council (CONICET), FCEN-UNCuyo, Mendoza 5501, Argentina
[3]Institute of Environment and Ecology, Tsinghua Shenzhen International Graduate School, Tsinghua University, Shenzhen 518055, China
[4]Atmospheric Chemistry Observations & Modeling Laboratory, National Center for Atmospheric Research, Boulder, CO, USA
[5]Centre for Climate Change Research, Indian Institute of Tropical Meteorology, Ministry of Earth Sciences, Pune, 411008 India
[6]Instituto de Astrofísica de Andalucía, CSIC, 18008 Granada, Spain
[7]Deutsches Zentrum für Luft- und Raumfahrt (DLR), Institut für Physik der Atmosphäre, Oberpfaffenhofen, Germany
[8]Lancaster Environment Centre, Lancaster University, Lancaster, UK
[9]School of Chemistry, University of Leeds, LS2 9JT Leeds, UK
[10]CICERO Center for International Climate Research, 0318 Oslo, Norway
[11]Climate and Global Dynamics Laboratory, National Center for Atmospheric Research, Boulder, CO, USA
[$]Present address: Department of Civil and Environmental Engineering, The Hong Kong Polytechnic University, Hong Kong, China

[*]Corresponding author. Email: a.saiz@csic.es
[#] These authors contributed equally.

## This PDF file includes:

Supplementary Text ST1 to ST4
Supplementary Tables S1 to S10
Supplementary Figures S1 to S11

# Supplementary Text

## ST1. Sensitivity analysis of SLH sources, abundance and radiative influence on SLCF

To quantitatively evaluate the magnitude of the radiative effect (RE) induced by short-lived halogens (SLH) on the climate system, and address the model uncertainties associated with the dominant processes controlling the anthropogenic amplification of natural emissions (AANE) that directly or indirectly impact on the global abundance of radiatively-active short-lived climate forcers (SLCF; in particular, ozone ($O_3$), methane ($CH_4$), stratospheric water vapour ($H_2O^{strat}$) and aerosols), we perform a thorough set of sensitivities using our CESM/CAM-Chem (Community Earth System Model & Community Atmospheric Model with interactive Chemistry) SLH benchmark model configuration. This consists of a total of 44 additional simulations where: *i)* the halogen source strength for each individual species was increased/decreased within the accepted range of values described in the literature; *ii)* individual reactions implemented in our state-of-the-art tropospheric SLH chemical mechanism where turned on/off; and *iii)* different boundary conditions and/or emission trends of the main anthropogenic pollutants during each period of time were considered. To highlight the dominant role played by ocean-emitted halogens over the contribution from anthropogenic (ANT) sources, the sensitivity analysis was performed for both AANE+ANT as well as for only AANE scenarios (see EDT1 and section 'CESM (CAM-Chem) model configuration and experiments design' in Methods for the definition of each benchmark simulation). Supplementary Table S1 summarizes the main reactions controlling the influence of SLH on the abundance of the main SLCF considered in this work.

In order to optimize the total number of simulations and reduce the computational cost, we initially focused the uncertainty analysis on present-day conditions (PD2020) and performed independent sensitivities where the halogen burden of only one of the chlorine (*high_Cl*), bromine (*low_Br*) or iodine (*high_I* and *low_I*) abundances was modified with respect to the benchmark CESM/CAM-Chem setup while maintaining the others unaltered (see Supplementary Table S2). Additional simulations considering the benchmark halogen emission strengths but without activating model updates related to SLH influence on aerosols (*aerchem_OFF*), as well as on polar sea-ice emissions (*polar_OFF*), were performed. Even though only 3-year long simulations were found to be necessary to allow a tropospheric halogen stabilization, we performed 5-year long iterations for each individual run and computed the model multi-year mean uncertainty as two times the standard deviation (2σ) of the global mean RE (see section 'The RRTMG radiation module in CESM' in Methods). Finally, and due to the larger atmospheric lifetime of methane compared to ozone and SLH, we performed additional 15-year long sensitivities where instead of considering cyclical $CH_4$ emission and boundary conditions representative of year 2020, we used initial $CH_4$ conditions representative of year 2000 and implemented model trends for the 2000-2020 period following Li et al.[39] (*CH4_icbc*). Below we provide a point-by-point description of the rationale beyond the selection of each of the independent sensitivities performed for PD2020 conditions:

- ***high_Cl*:** CESM/CAM-Chem includes three main sources of short-lived chlorine: *i)* natural halocarbon emissions from the ocean; *ii)* inorganic chlorine ($Cl_y$) emissions resulting from the sea-salt aerosol (SSA) recycling of halogen reservoirs, including acid-displacement; and *iii)* continental anthropogenic sources mostly related to biomass burning. Note that the SSA-dehalogenation process is computed online and depends on the reactive uptake recycling efficiency (gamma-factor) for different chlorine reservoir species (e.g., $ClONO_2$,

$ClNO_2$ and HOCl)[26] and nitric acid ($HNO_3$)[64]. The SSA-dehalogenation source in CESM/CAM-Chem dominates over the others (~23 Tg Cl $yr^{-1}$ compared to a total source of ~32 Tg Cl $yr^{-1}$, see EDT2), but is almost 4 times smaller than an equivalent implementation of SSA-dehalogenation in the TOMCAT model (~90 Tg Cl $yr^{-1}$)[64] and between 1.5 and 3 times lower than the range of values estimated by Graedel et al.[107] (~37−73 Tg Cl $yr^{-1}$). Therefore, we performed an upper limit sensitivity by doubling the uptake efficiency (2×gamma-factor) of the heterogeneous recycling reactions (see Supplementary Table S2). We found it not necessary to perform a *low_Cl* sensitivity as CESM/CAM-Chem inorganic chlorine levels are lower than the $Cl_y$ burden in the few chemistry-climate models that have implemented tropospheric chlorine chemistry[29,64]. Note, however, that the chorine burden relies not only on $Cl_y$ emissions, but also on the online computation of wet-scavenging and dry-deposition, which is parameterized in different ways between models. Consequently, the $Cl_y$ burden enhancement in CESM/CAM-Chem does not respond linearly to an increase in its dominant source (see Supplementary Text ST2). We highlight that, even if the *high_Cl* recycling efficiency is considered, the chlorine tropospheric levels in CESM/CAM-Chem are in the low edge of the estimated $Cl_y$ burdens of other models, and consequently its influence on the abundance of radiatively-active SLCF –directly or indirectly– may well be underestimated.

- ***low_Br:*** Bromine sources in CESM/CAM-Chem are equivalent to those for chlorine, although for the case of sea-salt bromine recycling, the implemented parameterization applies only to bromine reservoir species (e.g., $BrONO_2$, $BrNO_2$ and HOBr). The global SSA-debromification source in the current CESM/CAM-Chem setup reaches ~4 Tg $yr^{-1}$, which is approximately 30% larger than in previous simulations performed by our group (~2.9 Tg $yr^{-1}$)[55,93]. This is largely associated with the fact that previous "only bromine" studies neglected the tropospheric perturbations introduced by the $Cl_y$ sources considered here. Even though our current global inorganic bromine ($Br_y$) source is larger than the initial implementation obtained from other models (~1.5−2.0 Tg $yr^{-1}$)[108–110], it remains lower than the recent estimates obtained with GEOS-Chem when the reactivity of bromine with tropospheric aerosols is considered (3.5−6.4 Tg $yr^{-1}$)[59]. Note that as CESM/CAM-Chem includes independent washout efficiency for each individual species, our tropospheric BrO columns and vertical profiles are smaller than those obtained with GEOS-Chem[111]. Based on this, we performed only a lower limit sensitivity reducing the bromine recycling efficiency by 30% compared with our benchmark model configuration (0.7×gamma-factor). For a detailed description of the SSA-dehalogenation source in CESM/CAM-Chem, please see the Supplementary Material in Ordoñez et al.[26] and Fernandez et al.[55]. Given the minor role of anthropogenic bromine sources on the global bromine burden (see EDT3), we did not explore the contribution from these continental sources during the sensitivity analysis.

- ***low_I*** and ***high_I***: Implementation of iodine sources in CESM/CAM-Chem differs from those of bromine and chlorine, as sea-salt aerosol recycling of iodine species does not represent a net iodine source, but instead results only in a partitioning shift between reactive and reservoir species[30]. On top of the natural iodocarbon emission source arising from the ocean[26], the dominant source of inorganic iodine ($I_y$) to the atmosphere arises from the ozone-driven oxidation of aqueous iodide occurring at the seawater surface[19]. Indeed,

Prados-Roman et al.[38] showed that the implementation of this coupled ozone-iodine abiotic source in CESM/CAM-Chem was required to reproduce the observed levels of iodine monoxide (IO) over the marine boundary layer (MBL), and proposed for the first-time that ozone-deposition over the ocean and the subsequent iodine-emissions resulting from the oxidative processes resulted in a negative-feedback mechanism that regulated anthropogenic ozone pollution since pre-industrial times. Given the larger influence of tropospheric iodine chemistry on ozone destruction over the MBL and free troposphere, and despite the global oceanic $I_y$ source in CESM/CAM-Chem (~1.9 Tg $yr^{-1}$) is the smallest of all the models that have mapped iodine emissions based on the Prados-Roman et al.[38] original implementation (~2.7 Tg $yr^{-1}$ in GEOS-Chem[29] and (~2.9 Tg $yr^{-1}$ in SOCOL[112]), we performed both a lower limit (*low_I*) and upper limit (*high_I*) sensitivity where we respectively reduced by 15% and increased by 40% the oceanic iodine flux strength compared to the benchmark simulation. Here, is also worth mentioning that the ozone burden and ozone deposition rates computed with the standard CESM version (i.e., omitting the contribution from SLH)[51] that participated in the Coupled Model Intercomparison Project Phase 6 (CMIP6)[41] lie in the low edge of all earth system models that performed coupled tropospheric chemistry simulations. Indeed, the ozone dry deposition rates in CESM are between 5% to 30% lower than the multi-model CMIP6 mean (see Tables 1 and 2 in Griffiths et al.[41]) depending on the period of time considered, which is consistent with a conservative representation of iodine sources and impacts in our current SLH version of CESM/CAM-Chem.

- ***polar_OFF***: The implementation of polar halogen sources and chemistry arising from the Arctic and Antarctic sea-ice in CESM/CAM-Chem has been described in detail in Fernandez et al.[57]. The net polar-halogens source strength shows a pronounced seasonality and its inclusion in the model is necessary to reproduce the large chlorine, bromine and iodine enhancements that drive the Arctic and Antarctic ozone depletion events observed during spring. In order to evaluate the overall contribution of polar halogen sources with respect to the global- and annual- emission budget for each halogen family, we performed an additional sensitivity analysis by turning off the CESM/CAM-Chem polar module. Even though the influence of polar halogen sources on global tropospheric ozone possesses interesting features, we believe that extending the polar analysis here will be too specific and distract from the main point of the current sensitivity analysis. Thus, we have focused here only on addressing the uncertainty associated with the overall contribution of polar sea-ice sources to the global- and annual- mean halogen flux and burden, and leave for future work a detailed description of the seasonal influence of polar halogens on the global scale.

- ***aerchem_OFF***: Section 'CESM (CAM-Chem) model configuration and experiments design' in Methods describes in detail the benchmark AANE and AANE+ANT configurations, including the different sources, reactions and processes in which SLH participate. In particular, the current setup includes recent improvements and/or updates related to the formation of secondary organic aerosols (SOA), the production of sulfate aerosols and the heterogeneous recycling of bromine and chlorine species on anthropogenic aerosols[59,61]. Despite the continuous improvements in the representation of these heterogeneous processes in our SLH chemical-scheme[43,58,61], the description of gas-phase

reactions is more detailed than the simplified parameterizations used to represent the halogen-aerosol interaction. This is partially due to the recent migration from bulk to modal representation of tropospheric aerosols in CESM[54]. Thus, to provide a first-order uncertainty estimate of the halogen-aerosol interaction, we performed an additional sensitivity turning off all the direct reactions involving SLH and aerosols. Consequently, the aerosol burden and RE obtained for the *aerchem_OFF* sensitivity is indicative of the indirect influence of SLH on aerosol formation through changes in OH abundance.

- ***CH4_icbc***: Of all the SLCF considered in this work, methane is the one with the longest lifetime ($\tau \approx 10$ years) and, consequently, the estimated RE for this species will depend on both the initial condition (IC) and boundary condition (BC) considered. Indeed, Li et al.[39], highlighted the importance of performing ~40 years spin-up to allow for methane stabilization resulting from the SLH influence on the atmospheric oxidative capacity (e.g., OH abundance). Consequently, all of the benchmark NoSLH, AANE and AANE+ANT simulations during present-day were initialized from a simulation with already stabilized $CH_4$ levels, and were cycled with 2020 pollutant emissions during a 15-year period. The resulting $CH_4$ surface abundances in our model are in line with global observations (see section 'Evaluation of CESM (CAM-Chem) performance' in Methods and Supplementary Figure S10). Given the current trends in methane emissions and abundances, we performed an additional 15-year long sensitivity starting with IC/BC from year 2000 and forced the model with serial $CH_4$ emissions instead of cyclical. Consequently, the tropospheric methane burden and total RE from methane for the *CH4_icbc* sensitivity is the only one indicative of the modelled response dependence to the background levels of methane, which in turn are directly affected by chlorine and indirectly affected by SLH-driven changes in OH[39]. Note that in order to compute a consistent RE for the present-day *CH4_icbc* sensitivities, we performed additional 15-year long simulations with identical $CH_4$ IC/BC for the corresponding NoSLH configuration (see EDT1 and Supplementary Table S2).

Based on the main results of each of the above individual sensitivities for present-day conditions (results summarized in Supplementary Text ST2), we configured a combined-halogen scenario where we used the maximum and minimum source strengths from each independent halogen case, and compared the halogen burden and SLH-driven RE with respect to those obtained when only a single halogen species was increased or decreased. These combined-halogen scenarios (*SLH_high* and *SLH_low*) resulted in overall changes that reflect the additional ozone depletion efficiency due to inter-halogen reactions (i.e., $ClO_x + BrO_x$, $BrO_x + IO_x$ and $ClO_x + IO_x$ coupling) occurring both in the troposphere[30] and stratosphere[46]. Note that we performed the *SLH_high* and *SLH_low* sensitivities for both the AANE+ANT and AANE conditions. In summary, the complete set of sensitivities performed for all past, present and future scenarios were configured as follows:

- ***SLH_low:*** We decreased the recycling efficiency of the SSA-debromification source by 30% as well as the oceanic surface iodine flux by 15% following the *low_Br* and *low_I* sensitivities, while maintaining the chlorine emissions, polar sources, aerosol chemical-scheme and methane IC/BC unaltered. Even though the largest emission ranges for bromine apply to present-time conditions (where SSA-dehalogenation maximizes, see Supplementary Table S3), while for the case of iodine the largest uncertainties are

associated with pre-industrial times (where individual CMIP6 models show the largest variability, see Table 2 in Griffiths et al.[41]), we applied both 15% and 30% halogen reductions during all periods of time. Although the uncertainty analysis presented here has been performed with a single model, we impose the largest possible range of values on tropospheric halogen sources provided in the literature. In doing so we acknowledge that the strength of the SSA-dehalogenation source during different time periods may change depending on the SSA lifting and deposition parameterizations considered[51,54].

- ***SLH_high:*** We increased the efficiency of acid-displacement driven SSA-dechlorification by 100% (i.e., 2×gamma-factor), as well as the flux strength of the oceanic iodine source by 40%, while maintaining the bromine emissions and remaining processes unaltered. Note that besides the significant enhancement in the emission of both chlorine and iodine, our modelled tropospheric halogen burdens are still lower than those obtained with other global models that implemented tropospheric halogen chemistry. This implies that in any case, our upper limit radiative implications of SLH on the climate system are conservative.

- ***CH4_icbc***: The 15 years-long *CH4_icbc* sensitivity was also performed for pre-industrial conditions, as well as for each of the future Representative Concentration Pathways (RCP) scenarios, RCP 6.0 and RCP 8.5. For pre-industrial conditions, and due to the lack of observations to estimate a methane source trend, we used cyclical emission strength but performed additional 15year long simulation to reach a different IC. For each of the RCPs, the sensitivities were initialized in year 2085 and considered the estimated trend in $CH_4$ evolution from 2085 to 2100 instead of using cyclical values for year 2100. Similar to the present-day simulations, we performed additional 15-year long simulations with equivalent methane IC/BC for the corresponding NoSLH benchmark configuration for both RCPs.

Supplementary Table S2 summarizes the complete set of simulations performed. In order to derive a consistent error estimate of the radiative influence of SLH during all time periods, we only used the *SLH_high*, *SLH_low* and *CH4_icbc* sensitivities to determine the uncertainty on the individual and combined RE contributions arising from each individual SLCF. In doing so, we evaluated the model changes in the online emission flux of each individual source of organic and inorganic halogen species (Supplementary Tables S3 and S4) controlling the global mean atmospheric abundance of inorganic chlorine, bromine and iodine (Supplementary Table S5), as well as on the consequent changes on the tropospheric and stratospheric burdens of $O_3$, $CH_4$, $H_2O^{strat}$ and aerosols (Supplementary Table S6). Supplementary Tables S7, S8 and S9 show the mean global RE for each sensitivity as well as the modelled uncertainty obtained for the multi-year average (expressed as mean $\pm$ 2$\sigma$), while EDT5 summarizes the mean and uncertainty range of the radiative effect associated with the atmospheric levels of halogens and SLCF represented in the complete set of CESM/CAM-Chem sensitivities (expressed as mean $\pm$ range/2). Results indicate that the dominant factors determining the uncertainty of the SLH-driven RE are the predicted levels of tropospheric chlorine, bromine and iodine within each scenario. Below we provide a comprehensive analysis of the main conclusions achieved throughout the sensitivity analysis, and highlight the main similarities and differences between the results obtained for present-day, pre-industrial and future conditions.

## ST2: Model response to changes in the recycling efficiency of halogen emissions

EDT2 presents the emission flux of short-lived halocarbons ($SLH^{Cl}$, $SLH^{Br}$ and $SLH^{I}$) and total inorganic halogens ($Cl_y$, $Br_y$ and $I_y$) emitted at the model surface as well as resulting from SSA-dehalogenation for the simulations described in EDT1. The contribution from oceanic SLH-halocarbons (61−70 Gg Cl $yr^{-1}$, 594−752 Gg Br $yr^{-1}$ and 565−639 Gg I $yr^{-1}$; range of values across pre-industrial, present-day and future scenarios) are significantly smaller than the contribution resulting from the heterogeneous recycling of inorganic halogen species. In agreement with previous reports[38], the dominant source of chlorine and bromine is SSA-dehalogenation (~23 Tg Cl $yr^{-1}$ and ~4 Tg Br $yr^{-1}$ for present-day conditions), while for iodine the oceanic source of HOI and $I_2$ (total of ~1.9 Tg I $yr^{-1}$) dominates. Only for the case of chlorine, and due to the rapid increase in emissions observed in the recent past over East Asia[113], the anthropogenic $SLH^{Cl}$ contribution during present-day (~1.5 Tg Cl $yr^{-1}$) is larger than the natural oceanic sources. Nevertheless, its contribution is more than 10 times smaller than the SSA-dehalogenation and is expected to be reduced in the future[99]. Note that anthropogenic emissions occurring mostly from coal-burning activities (~7.8 Tg Cl $yr^{-1}$) represent also a significant source of chlorine within continental domains. Supplementary Figures S1-S3 show the spatial distribution of the natural and anthropogenic chlorine, bromine and iodine sources for each configuration and period of time.

Supplementary Table S3 shows the halogen emission flux for all sensitivities performed during present-day, which is directly linked to the change in recycling efficiency imposed to the main processes controlling the online emissions of inorganic halogens. Note that in all cases, the percentage flux change (ΔFlux) relative to the benchmark AANE or AANE+ANT configurations is presented. Most notably, all the SLH-halocarbon sources for present-day conditions remain identical for all sensitivities because they are not computed online but instead taken from the Ordoñez et al.[26] inventory. For the case of the surface inorganic sources, it is worth noting that AANE results for chlorine and bromine are indicative of the polar sea-ice emissions, and consequently, they remain almost unaltered for all sensitivities with exception of the *polar_OFF* case where these contributions are zeroed. For the case of iodine, the *SLH_low* and *SLH_high* scenarios show approximately a 10% reduction and 30% enhancement on the oceanic iodine source, which are very similar to those obtained for the *low_I* and *high_I* sensitivities. Similarly, the dominant SSA-dehalogenation source for the *SLH_low* and *SLH_high* scenarios increase (decrease) the chlorine (bromine) flux by ~30% (~−55%), which is in line with the results obtained with the individual *high_Cl* (*low_Br*) sensitivity. Finally, it is worth noting that given the larger contribution from anthropogenic chlorine surface emissions (compared to bromine and iodine) for the AANE+ANT configuration, the percentage change of the natural $Cl_y$ source is reduced relative to the only AANE case.

Supplementary Table S4 show equivalent results for the main sensitivities (*SLH_low*, *SLH_high* and *CH4_icbc*) performed during pre-industrial, present-day and future scenarios. Percentage changes with respect to the benchmark simulation are equivalent during all time periods, resulting in 24−30% enhancement in acid-displacement chlorine emissions, 49−55% reduction for SSA-debromification; and approximately 10−12% decrease (*SLH_low*) and 29−31% increase (*SLH-high*) on the oceanic iodine flux. The very small (< 3%) changes observed for the *CH4_icbc* case are due to the atmospheric changes in the oxidative capacity of the troposphere when the IC/BC of pollutants are changed, but do not represent a significant change in the global burden for any of

the halogen families. Supplementary Figure S4 summarizes and compares the main results compiled in Supplementary Tables S3 and S4.

## ST3: Tropospheric and stratospheric changes on SLH and SLCF abundances

EDT3 summarizes the global-mean surface abundance and tropospheric burden for inorganic chlorine, bromine and iodine. Most notably, both AANE and AANE+ANT setups present very similar halogen abundances, highlighting that oceanic-driven natural processes, which are largely controlled by inorganic-halogen recycling on SSA and/or ozone deposition-rates over the sea-surface, must be considered to obtain a realistic representation of halogen abundance in the troposphere. For the case of chlorine, the contribution from anthropogenic SLH-chlorocarbons are larger than the natural oceanic emissions, but smaller than the continental $Cl_y$ source. Supplementary Figures S5-S7 show the tropospheric averaged spatial distribution and mean zonal average of each individual SLH species during each period of time.

Supplementary Table S5 shows results for the combined *SLH_low* and *SLH_high* cases. The tropospheric abundance of $Cl_y$ increased by 23−31%, while the $Br_y$ levels decreased by 21−30%. Most notably, the change in halogen burden does not respond linearly to the net change in halogen emissions, as many other processes such as washout and dry-deposition occur simultaneusly. On the same line, $I_y$ abundance during all time periods is reduced by 4−8% for *SLH_low* and increased by 13−21% for *SLH_high*. Supplementary Figure S8 summarizes and compares the SLH surface and tropospheric abundance compiled in Supplementary Table S5.

Short-lived climate forcing agents such as methane, ozone and aerosols are significant contributors to global warming[34,36], therefore the atmospheric oxidizing capacity (e.g., hydroxyl radical concentrations, which in turn is influenced by halogens) that affects their baseline concentrations and lifetime in the atmosphere also affects climate over time in different ways. EDT4 compiles the change in abundance of ozone, methane, water vapour and aerosols induced by natural and anthropogenic SLHs, including the percentage change of each species with respect to the corresponding benchmark simulation where the contribution of SLH sources and chemistry has been omitted (i.e., NoSLH). For ozone, both the tropospheric and stratospheric column reductions driven by SLH are of equivalent magnitude. Indeed, the tropospheric ozone percentage change remains practically constant during all time periods (~−15%) due to the halogen buffering effect on the oxidizing capacity[37]. The absolute stratospheric ozone change during RCP 8.5 is equivalent to present-day due to the larger recovery of the ozone layer induced by the stratospheric cooling moving into the future. However, note that the larger percentage change occurs during present time because the SLH influence on stratospheric ozone peaks during periods of high anthropogenic reactive chlorine levels in the stratosphere, which in turn depends on the evolution of long-lived ozone-depleting substances [77].

Supplementary Table S6 shows the tropospheric and stratospheric abundance of each of the main SLCF considered in this work, as well as their changes with respect to the benchmark configuration during all time periods for the *SLH_low*, *SLH_high* and *CH4_icbc* sensitivities. The global tropospheric ozone burden is, at most, reduced by −2.3% and increased by 2.2% for the high and low emissions scenarios, regardless of the period of time considered. This highlights that the SLH influence on tropospheric ozone is robust. Given the longer lifetime of methane, its model abundance depends on the spin-up, IC/BC and emission trends considered. Consequently, the

methane burden within the *CH4_icbc* sensitivity is increased by ~13% during pre-industrial times and only by ~1.2% during present-day conditions, highlighting the importance of properly determining the modelled $CH_4$ pre-industrial baseline to reduce model uncertainties when performing climate radiative estimations. Similarly, the total $CH_4$ burden is increased (decreased) by ~6% (~−6%) relative to the corresponding RCP 6.0 (RCP8.5) future benchmark scenario for the *CH4_icbc* sensitivity, which is consistent with the assumed methane emissions trends[39]. For the case of aerosols, tropospheric burdens typically differ by 1−5%, with maximum values reaching ~10% over localized regions, although we acknowledge that the representation of the SLH-aerosol interaction is more simplified than the representation of gas-phase reactions. Supplementary Figure S9 summarizes and compares the main results compiled in Supplementary Table S6.

## ST4: Model uncertainties of the SLH-driven radiative effect for the main SLCF

Supplementary Table S7 shows the estimated RE induced by SLH on ozone, methane, stratospheric water-vapour and aerosols during present-day conditions for the complete set of sensitivities, including the resulting model uncertainty for each individual simulation expressed as the multi-year global mean ± 2σ. Three main features become evident from the compiled dataset: first, the multi-year variability (2σ) in the RE for each simulation is smaller than the mean change in RE for the different sensitivities, highlighting that the uncertainty on the SLH impact on the climate system is dominated by the different halogen burden obtained for each individual case; second, that with exception of methane, the smallest and largest RE for the main radiatively active SLCF are obtained for the *SLH_low* and *SLH_high* sensitivities, respectively; third, the largest change on the modelled methane RE with respect to the benchmark setup arises from the *CH4_icbc* sensitivity. Consequently, we confirm that these combined scenarios are the most important ones to estimate the radiative uncertainty. Note that the final three rows for each of the only AANE and AANE+ANT scenarios in Supplementary Table S7 summarize the minimum, maximum and range of values obtained in the sensitivities, and that the difference between the maximum and minimum mean values for each sensitivity is always larger than 2σ for each independent case.

Supplementary Tables S8 and S9 show the modelled RE induced by SLH during pre-industrial and present-day conditions, as well as during both RCP 6.0 and RCP 8.5 scenarios, respectively. In addition to the individual results for each sensitivity, the final bold row for each of the Natural, AANE and AANE+ANT configurations compiles the mean radiative effect of each simulation with its correspondent uncertainty expressed as (RE ± range/2), where range/2 is half of the largest difference ((maximum – minimum)/2) of the mean RE of all the sensitivities performed for each configuration during a single time period. Note that the uncertainty for the net effect of the group of gases and aerosols is the sum of the independent uncertainties of all individual species within each group. The final bold results for each of the configurations shown in Supplementary Tables S8 and S9 are summarized in EDT5. Radiative effects changes are expressed as ΔRE ± error, where the uncertainty error is either: *i)* the largest uncertainty for the net (GAS+AER) ranges compiled in EDT5 for each individual benchmark simulation compared to the pre-industrial scenario; or *ii)* the sum of the ozone RE uncertainties for the two periods of time considered when computing ΔRE, as this is the radiatively active species presenting the largest absolute changes. In all cases we used the larger of the two approaches, although in most cases both *i)* and *ii)* provide identical results when rounded to two significant digits (e.g., uncertainty = 0.03 W $m^{-2}$ for present-day AANE+ANT).

# Supplementary Tables

**Supplementary Table S1. Main halogen reactions controlling the chemistry-climate influence of short-lived halogens on short-lived climate forcers.**

| Reaction number | Reactions [&] | Notes |
|---|---|---|
| R1 | $XY + hv \rightarrow X + Y$ | X,Y = Cl, Br, I |
| R2 | $CH_2XY + hv/OH \rightarrow X + Y$ | X,Y = Cl, Br, I |
| R3 | $X + O_3 \rightarrow XO$ | X = Cl, Br, I |
| R4 | $O_3 + hv \rightarrow O^1D + O_2$ | |
| R5 | $O^1D + H_2O \rightarrow 2OH$ | |
| R6 | $XO + HO_2 \rightarrow HOX$ | X = Cl, Br, I |
| R7 | $HOX + hv \rightarrow X + OH$ | X = Cl, Br, I |
| R8 | $ClNO_2 + hv \rightarrow Cl + NO_2$ | |
| R9 | $VOC + Y \rightarrow RO_2 + HY$ | Y = Cl, Br; R = carbon radical |
| R10 | $RO_2 + NO \rightarrow NO_2 + RO$ | R = carbon radical |
| R11 | $RO + O_2 \rightarrow HO_2$ + carbonyls | R = carbon radical |
| R12 | $HO_2 + NO \rightarrow NO_2 + OH$ | |
| R13 | $NO_2 + hv \rightarrow NO + O(^3P)$ | |
| R14 | $O(^3P) + O_2 \rightarrow O_3$ | |
| R15 | $NO_2 + O_3 \rightarrow NO_3$ | |
| R16 | $OH + CH_4 \rightarrow CH_3O_2 + H_2O$ | |
| R17 | $Cl + CH_4 \rightarrow CH_3O_2 + HCl$ | |
| R18 | $SO_2 + Z \rightarrow$ sulfate | Z = OH, $O_3$, $H_2O_2$, HOBr |
| R19 | $NO_2 + OH \rightarrow HNO_3$ | |
| R20 | $HNO_3 + NH_3 \leftrightarrow$ nitrate + ammonium | |
| R21 | $VOC + Z \rightarrow SOA$ | Z = OH, $O_3$, $NO_3$, Cl, Br |

[&] The complete SLH chemical-scheme, including all photolysis, bi-molecular, termolecular and heterogeneous reactions, has been described in detail in Ordoñez et al.[26], Fernandez et al.[55], Saiz-Lopez et al.[30,56], Li et al.[39] and Cuevas et al.[46]. For the complete set of SLH reactions included in CESM/CAM-Chem, see the supplementary material in Badía et al.[63].

**Supplementary Table S2: Sensitivity simulations performed to evaluate the uncertainty of the SLH-driven radiative effect.**

| Sensitivity[$] / Species[£] | Initial Condition | Boundary Condition | SLH-Aerosol Interaction | SLH-Polar Emissions | Oceanic Emissions[#] | Sea-salt Recycling[€] | |
|---|---|---|---|---|---|---|---|
| | $CH_4$ | $CH_4$ | $Cl_y$, $Br_y$ | $Cl_y$, $Br_y$, $I_y$ | $I_y$ | $Cl_y$ | $Br_y$ |
| **AANE/AANE+ANT** | **2020** | **cyclical 2020** | **ON** | **ON** | **flx_I** | **gamma_Cl** | **gamma_Br** |
| **CH4_icbc[&]** | **year 2000** | **serial 2000-2020** | **ON** | **ON** | **flx_I** | **gamma_Cl** | **gamma_Br** |
| **SLH_low** | **2020** | **cyclical 2020** | **ON** | **ON** | **0.85 × flx_I** | **gamma_Cl** | **0.7 × gamma_Br** |
| **SLH_high** | **2020** | **cyclical 2020** | **ON** | **ON** | **1.40 × flx_I** | **2.0 × gamma_Cl** | **gamma_Br** |
| low_I | 2020 | cyclical 2020 | ON | ON | 0.85 × flx_I | gamma_Cl | gamma_Br |
| high_I | 2020 | cyclical 2020 | ON | ON | 1.40 × flx_I | gamma_Cl | gamma_Br |
| low_Br | 2020 | cyclical 2020 | ON | ON | flx_I | gamma_Cl | 0.7 × gamma_Br |
| high_Cl | 2020 | cyclical 2020 | ON | ON | flx_I | 2.0 × gamma_Cl | gamma_Br |
| polar_OFF | 2020 | cyclical 2020 | ON | Turned OFF | flx_I | gamma_Cl | gamma_Br |
| aerchem_OFF | 2020 | cyclical 2020 | Turned OFF | ON | flx_I | gamma_Cl | gamma_Br |

[$] All sensitivities were performed for both AANE and AANE+ANT benchmark configurations described in EDT1, whose main parameters are shown in the first row of the table as a reference. All sensitivities were computed for present-time conditions, whereas only the main cases in bold (*CH4_icbc*, *SLH_low* and *SLH_high*) were computed during pre-industrial and future RCP 6.0 and RCP 8.5 scenarios. The main RCPs sensitivities considered initial conditions (boundary conditions) representative of 2085 (serial 2085-2100) obtained from Li et al.,[39]. The pre-industrial configuration follows Lamarque et al.,[106].

[&] In order to compute the SLH-driven RE for the *CH4_icbc* sensitivity, we also performed additional sensitivities imposing the 2020 IC and serial 2000-2020 BC to the NoSLH benchmark configuration.

[£] Total inorganic halogen abundance $X_y$ (with X = Cl, Br, I) is defined in the footnote of Supplementary Table S3.

[#] The inorganic iodine oceanic flux (flx_I) due to ozone deposition in seawater is computed online following Eqs. (2) and (3) in Prados-Roman et al.[38].

[€] gamma_Cl and gamma_Br are the default values for $YONO_2$, $YNO_2$ and HOY (Y = Cl, Br) heterogeneous recycling on sea-salt aerosols provided in Supplementary Table 4 of Ordoñez et al.[26]. An equivalent 2×factor was applied to the efficiency of $HNO_3 \rightarrow HCl$ conversion[64].

**Supplementary Table S3: Global mean SLH annual emission flux for the full set of sensitivities performed during present-day conditions.**

| Emission Flux[$] | | Surface Emissions | | | | | | | | | | | | Sea-salt Recycling | | | | | |
|---|---|---|---|---|---|---|---|---|---|---|---|---|---|---|---|---|---|---|---|
| | | SLH-Halocarbons[&] | | | | | | Inorganic Halogens[&] | | | | | | Inorganic Halogens[&] | | | | | |
| | | $SLH^{Cl}$ | | $SLH^{Br}$ | | $SLH^{I}$ | | $Cl_y$ | | $Br_y$ | | $I_y$ | | $Cl_y$ | | $Br_y$ | | $I_y$ | |
| Period (Simulation) | Sensitivity | Flux Gg $yr^{-1}$ | ΔFlux % | Flux Gg $yr^{-1}$ | ΔFlux % | Flux Gg $yr^{-1}$ | ΔFlux % | Flux Gg $yr^{-1}$ | ΔFlux % | Flux Gg $yr^{-1}$ | ΔFlux % | Flux Gg $yr^{-1}$ | ΔFlux % | Flux Gg $yr^{-1}$ | ΔFlux % | Flux Gg $yr^{-1}$ | ΔFlux % | Flux Gg $yr^{-1}$ | ΔFlux % |
| Present-day (AANE) | **AANE** | **61.7** | **-** | **614.8** | **-** | **589.8** | **-** | **74.6** | **-** | **553.4** | **-** | **1831.9** | **-** | **23310.0** | **-** | **4026.4** | **-** | **0.0** | **-** |
| | **CH4_icbc** | **61.7** | **0.0** | **614.8** | **0.0** | **589.8** | **0.0** | **73.7** | **-1.2** | **551.1** | **-0.4** | **1817.7** | **-0.8** | **23155.6** | **-0.7** | **3922.8** | **-2.6** | **0.0** | **-** |
| | **SLH_low** | **61.7** | **0.0** | **614.8** | **0.0** | **589.8** | **0.0** | **75.3** | **1.0** | **551.8** | **-0.3** | **1637.9** | **-10.6** | **22873.3** | **-1.9** | **1810.9** | **-55.0** | **0.0** | **-** |
| | **SLH_high** | **61.7** | **0.0** | **614.8** | **0.0** | **589.8** | **0.0** | **74.6** | **0.0** | **545.5** | **-1.4** | **2396.8** | **30.8** | **30399.2** | **30.4** | **3945.2** | **-2.0** | **0.0** | **-** |
| | low_I | 61.7 | 0.0 | 614.8 | 0.0 | 589.8 | 0.0 | 74.6 | 0.0 | 552.3 | -0.2 | 1596.7 | -12.8 | 23321.1 | 0.0 | 4072.6 | 1.1 | 0.0 | - |
| | high_I | 61.7 | 0.0 | 614.8 | 0.0 | 589.8 | 0.0 | 74.4 | -0.3 | 553.2 | 0.0 | 2427.4 | 32.5 | 23285.6 | -0.1 | 4095.8 | 1.7 | 0.0 | - |
| | low_Br | 61.7 | 0.0 | 614.8 | 0.0 | 589.8 | 0.0 | 75.2 | 0.8 | 551.3 | -0.4 | 1880.1 | 2.6 | 22881.4 | -1.8 | 1858.9 | -53.8 | 0.0 | - |
| | high_Cl | 61.7 | 0.0 | 614.8 | 0.0 | 589.8 | 0.0 | 74.8 | 0.4 | 546.7 | -1.2 | 1812.8 | -1.0 | 30406.3 | 30.4 | 3916.5 | -2.7 | 0.0 | - |
| | polar_OFF | 61.7 | 0.0 | 614.8 | 0.0 | 589.8 | 0.0 | 0.0 | -100.0 | 0.0 | -100.0 | 1764.2 | -3.7 | 23338.7 | 0.1 | 3964.5 | -1.5 | 0.0 | - |
| | aerchem_OFF | 61.7 | 0.0 | 614.8 | 0.0 | 589.8 | 0.0 | 84.3 | 13.1 | 553.9 | 0.1 | 1897.5 | 3.6 | 23046.0 | -1.1 | 2821.5 | -29.9 | 0.0 | - |
| Present-day (AANE+ANT) | **AANE+ANT** | **1450.3** | **-** | **614.8** | **-** | **589.8** | **-** | **7812.6** | **-** | **753.4** | **-** | **1911.0** | **-** | **23392.6** | **-** | **3975.4** | **-** | **0.0** | **-** |
| | **CH4_icbc** | **1385.6** | **-4.5** | **614.8** | **0.0** | **589.8** | **0.0** | **7811.8** | **0.0** | **751.3** | **-0.3** | **1896.5** | **-0.8** | **23250.8** | **-0.6** | **3881.2** | **-2.4** | **0.0** | **-** |
| | **SLH_low** | **1451.4** | **0.1** | **614.8** | **0.0** | **589.8** | **0.0** | **7813.3** | **0.0** | **751.7** | **-0.2** | **1717.8** | **-10.1** | **22971.0** | **-1.8** | **1801.9** | **-54.7** | **0.0** | **-** |
| | **SLH_high** | **1437.0** | **-0.9** | **614.8** | **0.0** | **589.8** | **0.0** | **7812.3** | **0.0** | **745.4** | **-1.1** | **2471.3** | **29.3** | **30480.3** | **30.3** | **3894.5** | **-2.0** | **0.0** | **-** |
| | low_I | 1447.0 | -0.2 | 614.8 | 0.0 | 589.8 | 0.0 | 7812.6 | 0.0 | 752.3 | -0.1 | 1677.9 | -12.2 | 23403.6 | 0.0 | 4018.5 | 1.1 | 0.0 | - |
| | high_I | 1445.3 | -0.3 | 614.8 | 0.0 | 589.8 | 0.0 | 7812.2 | 0.0 | 750.6 | -0.4 | 2498.2 | 30.7 | 23397.5 | 0.0 | 4055.5 | 2.0 | 0.0 | - |
| | low_Br | 1450.8 | 0.0 | 614.8 | 0.0 | 589.8 | 0.0 | 7813.2 | 0.0 | 751.2 | -0.3 | 1957.8 | 2.4 | 22978.4 | -1.8 | 1848.7 | -53.5 | 0.0 | - |
| | high_Cl | 1438.2 | -0.8 | 614.8 | 0.0 | 589.8 | 0.0 | 7812.6 | 0.0 | 746.6 | -0.9 | 1892.3 | -1.0 | 30490.5 | 30.3 | 3867.2 | -2.7 | 0.0 | - |
| | polar_OFF | 1447.5 | -0.2 | 614.8 | 0.0 | 589.8 | 0.0 | 7737.2 | -1.0 | 200.4 | -73.4 | 1841.9 | -3.6 | 23422.3 | 0.1 | 3917.7 | -1.5 | 0.0 | - |
| | aerchem_OFF | 1456.3 | 0.4 | 614.8 | 0.0 | 589.8 | 0.0 | 7822.9 | 0.1 | 754.0 | 0.1 | 1976.3 | 3.4 | 23119.1 | -1.2 | 2734.8 | -31.2 | 0.0 | - |

[$] ΔFlux for each sensitivity (e.g., *SLH_low*) is computed relative to the AANE ($\Delta Flux^{SLH_low}_{AANE}$ = ($Flux^{SLH\_low}$ – $Flux^{AANE}$) / $Flux^{AANE}$ × 100%, no shading) or AANE+ANT ($\Delta Flux^{SLH\_low}_{AANE+ANT}$ = ($Flux^{SLH\_low}$ – $Flux^{AANE+ANT}$) / $Flux^{AANE+ANT}$ × 100%, grey shading) configurations.

[&] The global annual mean source strength of SLH is computed as follows: $SLH^{Cl}$ = $CHCl_3$ + $CH_2Cl_2$ + $C_2Cl_4$ + $C_2H_4Cl_2$ + $C_2HCl_3$ + $CH_2BrCl$ + $CH_2ICl$ + $CHBrCl_2$ + $CHBr_2Cl$; $SLH^{Br}$ = $CHBr_3$ + $CH_2Br_2$ + $CH_2BrCl$ + $CH_2IBr$ + $CHBrCl_2$ + $CHBr_2Cl$; $SLH^{I}$ = $CH_3I$ + $CH_2I_2$ + $CH_2ICl$ + $CH_2IBr$; while the total inorganic halogen abundance is defined as: $Cl_y$ = Cl + $Cl_2$ + ClO + OClO + $Cl_2O_2$ + HCl + HOCl + $ClONO_2$ + $ClNO_2$ + BrCl + ICl; $Br_y$ = Br + $Br_2$ + BrO + HBr + HOBr + $BrONO_2$ + $BrNO_2$ + BrCl + IBr; $I_y$ = I + $I_2$ + IO + OIO + HI + HOI + $IONO_2$ + $INO_2$ + INO + IBr + ICl + $I_2O_2$ + $I_2O_3$ + $I_2O_4$. For total mass computations, only the halogen mass is considered, whereas for the sum of mixing ratios the halogen atomicity for each species is considered.

**Supplementary Table S4: Global mean SLH annual emission flux for the main sensitivity simulations.**

| Emission Flux$ | | | Surface Emissions | | | | | | | | | | | | Sea-salt Recycling | | | | | |
|---|---|---|---|---|---|---|---|---|---|---|---|---|---|---|---|---|---|---|---|---|
| | | | SLH-Halocarbons | | | | | | Inorganic Halogens | | | | | | Inorganic Halogens | | | | | |
| | | | $SLH^{Cl}$ | | $SLH^{Br}$ | | $SLH^{I}$ | | $Cl_y$ | | $Br_y$ | | $I_y$ | | $Cl_y$ | | $Br_y$ | | $I_y$ | |
| Period | Simulation | Sensitivity | Flux | ΔFlux | Flux | ΔFlux | Flux | ΔFlux | Flux | ΔFlux | Flux | ΔFlux | Flux | ΔFlux | Flux | ΔFlux | Flux | ΔFlux | Flux | ΔFlux |
| | | | $Gg\ yr^{-1}$ | % | $Gg\ yr^{-1}$ | % | $Gg\ yr^{-1}$ | % | $Gg\ yr^{-1}$ | % | $Gg\ yr^{-1}$ | % | $Gg\ yr^{-1}$ | % | $Gg\ yr^{-1}$ | % | $Gg\ yr^{-1}$ | % | $Gg\ yr^{-1}$ | % |
| Pre-industrial | Natural | Natural | 60.8 | - | 593.7 | - | 584.5 | - | 66.6 | - | 542.1 | - | 938.6 | - | 6378.0 | - | 2751.9 | - | 0.0 | - |
| | | CH4_icbc | 60.8 | 0.0 | 593.7 | 0.0 | 584.5 | 0.0 | 66.6 | 0.0 | 545.0 | 0.5 | 971.5 | 3.5 | 6399.3 | 0.3 | 2662.0 | -3.3 | 0.0 | - |
| | | SLH_low | 60.8 | 0.0 | 593.7 | 0.0 | 584.5 | 0.0 | 67.8 | 1.8 | 544.3 | 0.4 | 847.3 | -9.7 | 6149.6 | -3.6 | 1342.6 | -51.2 | 0.0 | - |
| | | SLH_high | 60.8 | 0.0 | 593.7 | 0.0 | 584.5 | 0.0 | 66.2 | -0.7 | 534.7 | -1.4 | 1217.2 | 29.7 | 7894.0 | 23.8 | 2655.8 | -3.5 | 0.0 | - |
| Present-day | AANE | AANE | 61.7 | - | 614.8 | - | 589.8 | - | 74.6 | - | 553.4 | - | 1831.9 | - | 23310.0 | - | 4026.4 | - | 0.0 | - |
| | | CH4_icbc | 61.7 | 0.0 | 614.8 | 0.0 | 589.8 | 0.0 | 73.7 | -1.2 | 551.1 | -0.4 | 1817.7 | -0.8 | 23155.6 | -0.7 | 3922.8 | -2.6 | 0.0 | - |
| | | SLH_low | 61.7 | 0.0 | 614.8 | 0.0 | 589.8 | 0.0 | 75.3 | 1.0 | 551.8 | -0.3 | 1637.9 | -10.6 | 22873.3 | -1.9 | 1810.9 | -55.0 | 0.0 | - |
| | | SLH_high | 61.7 | 0.0 | 614.8 | 0.0 | 589.8 | 0.0 | 74.6 | 0.0 | 545.5 | -1.4 | 2396.8 | 30.8 | 30399.2 | 30.4 | 3945.2 | -2.0 | 0.0 | - |
| | AANE+ANT | AANE+ANT | 1450.3 | - | 614.8 | - | 589.8 | - | 7812.6 | - | 753.4 | - | 1911.0 | - | 23392.6 | - | 3975.4 | - | 0.0 | - |
| | | CH4_icbc | 1443.0 | 0.5 | 614.8 | 0.0 | 589.8 | 0.0 | 7811.8 | 0.0 | 751.3 | -0.3 | 1896.5 | -0.8 | 23250.8 | -0.6 | 3881.2 | -2.4 | 0.0 | - |
| | | SLH_low | 1454.9 | 0.3 | 614.8 | 0.0 | 589.8 | 0.0 | 7813.3 | 0.0 | 751.7 | -0.2 | 1717.8 | -10.1 | 22971.0 | -1.8 | 1801.9 | -54.7 | 0.0 | - |
| | | SLH_high | 1440.5 | -0.7 | 614.8 | 0.0 | 589.8 | 0.0 | 7812.3 | 0.0 | 745.4 | -1.1 | 2471.3 | 29.3 | 30480.3 | 30.3 | 3894.5 | -2.0 | 0.0 | - |
| RCP 6.0 | AANE | AANE | 66.6 | - | 701.1 | - | 621.1 | - | 69.0 | - | 557.4 | - | 1684.7 | - | 10930.9 | - | 2990.3 | - | 0.0 | - |
| | | CH4_icbc | 66.6 | 0.0 | 701.1 | 0.0 | 621.1 | 0.0 | 69.6 | 0.9 | 560.9 | 0.6 | 1747.2 | 3.7 | 10876.3 | 0.5 | 3062.4 | 2.4 | 0.0 | - |
| | | SLH_low | 66.6 | 0.0 | 701.1 | 0.0 | 621.1 | 0.0 | 69.3 | 0.5 | 555.1 | -0.4 | 1506.8 | -10.6 | 10565.4 | -3.3 | 1485.3 | -50.3 | 0.0 | - |
| | | SLH_high | 66.6 | 0.0 | 701.1 | 0.0 | 621.1 | 0.0 | 68.2 | -1.2 | 548.3 | -1.6 | 2183.2 | 29.6 | 13921.9 | 27.4 | 2948.0 | -1.4 | 0.0 | - |
| | AANE+ANT | AANE+ANT | 569.5 | - | 701.1 | - | 621.1 | - | 7773.2 | - | 588.4 | - | 1694.8 | - | 10979.7 | - | 2953.8 | - | 0.0 | - |
| | | CH4_icbc | 566.6 | 0.5 | 701.1 | 0.0 | 621.1 | 0.0 | 7807.8 | 0.4 | 585.5 | 0.5 | 1830.6 | 8.0 | 10924.8 | 0.5 | 3020.0 | 2.2 | 0.0 | - |
| | | SLH_low | 570.8 | 0.2 | 701.1 | 0.0 | 621.1 | 0.0 | 7773.5 | 0.0 | 585.9 | -0.4 | 1516.9 | -10.5 | 10620.6 | -3.3 | 1472.1 | -50.2 | 0.0 | - |
| | | SLH_high | 560.9 | -1.5 | 701.1 | 0.0 | 621.1 | 0.0 | 7772.2 | 0.0 | 579.3 | -1.5 | 2191.1 | 29.3 | 13973.9 | 27.3 | 2914.2 | -1.3 | 0.0 | - |
| RCP 8.5 | AANE | AANE | 69.5 | - | 751.6 | - | 638.6 | - | 82.8 | - | 664.1 | - | 2801.5 | - | 16141.3 | - | 2849.5 | - | 0.0 | - |
| | | CH4_icbc | 69.5 | 0.0 | 751.6 | 0.0 | 638.6 | 0.0 | 83.3 | 0.6 | 663.5 | -0.1 | 2781.4 | -0.7 | 16679.9 | 3.3 | 2948.5 | 3.5 | 0.0 | - |
| | | SLH_low | 69.5 | 0.0 | 751.6 | 0.0 | 638.6 | 0.0 | 82.4 | -0.5 | 655.1 | -1.4 | 2469.6 | -11.8 | 15880.4 | -1.6 | 1440.7 | -49.4 | 0.0 | - |
| | | SLH_high | 69.5 | 0.0 | 751.6 | 0.0 | 638.6 | 0.0 | 81.9 | -1.1 | 652.8 | -1.7 | 3633.2 | 29.7 | 20902.6 | 29.5 | 2946.7 | 3.4 | 0.0 | - |
| | AANE+ANT | AANE+ANT | 475.8 | - | 751.6 | - | 638.6 | - | 5721.5 | - | 702.9 | - | 2814.3 | - | 16202.5 | - | 2843.4 | - | 0.0 | - |
| | | CH4_icbc | 473.4 | 0.5 | 751.6 | 0.0 | 638.6 | 0.0 | 5692.9 | 0.5 | 699.3 | 0.5 | 2864.1 | 1.8 | 16121.5 | 0.5 | 2943.1 | 3.5 | 0.0 | - |
| | | SLH_low | 478.5 | 0.6 | 751.6 | 0.0 | 638.6 | 0.0 | 5721.1 | 0.0 | 693.8 | -1.3 | 2482.9 | -11.8 | 15942.3 | -1.6 | 1440.9 | -49.3 | 0.0 | - |
| | | SLH_high | 473.0 | -0.6 | 751.6 | 0.0 | 638.6 | 0.0 | 5720.5 | 0.0 | 691.6 | -1.6 | 3644.1 | 29.5 | 20970.4 | 29.4 | 2940.9 | 3.4 | 0.0 | - |

$ Total inorganic halogen abundance $X_y$ (with X = Cl, Br, I) and Δflux are defined in the footnote of Supplementary Table S3. Results for AANE+ANT are highlighted by grey shading.

**Supplementary Table S5: Global mean surface mixing ratio and tropospheric burden changes of total inorganic halogens for the main sensitivities.**

| Species Abundance[$] | | | $Cl_y$ | | | | $Br_y$ | | | | $I_y$ | | | |
|---|---|---|---|---|---|---|---|---|---|---|---|---|---|---|
| | | | Surface | | Tropo[&] | | Surface | | Tropo[&] | | Surface | | Tropo[&] | |
| Period | Simulation | Sensitivity | vmr | Δvmr | Burden | ΔBurden | vmr | Δvmr | Burden | ΔBurden | vmr | Δvmr | Burden | ΔBurden |
| | | | pptv | % | Tg | % | pptv | % | Tg | % | pptv | % | Tg | % |
| Pre-industrial | Natural | Natural | 6.1 | - | 59.8 | - | 3.0 | - | 23.9 | - | 1.5 | - | 11.2 | - |
| | | CH4_icbc | 6.1 | -0.2 | 59.4 | -0.7 | 3.0 | -2.3 | 23.3 | -2.4 | 1.5 | 1.9 | 11.3 | 0.9 |
| | | SLH_low | 6.1 | -1.1 | 60.5 | 1.1 | 2.1 | -30.3 | 18.0 | -24.8 | 1.4 | -6.5 | 10.7 | -4.0 |
| | | SLH_high | 7.2 | 17.0 | 76.2 | 27.5 | 2.9 | -3.2 | 22.7 | -4.9 | 1.8 | 19.3 | 12.7 | 13.4 |
| Present-day | AANE | AANE | 26.4 | - | 147.9 | - | 4.2 | - | 25.9 | - | 2.3 | - | 13.8 | - |
| | | CH4_icbc | 26.0 | -1.5 | 149.9 | 1.4 | 4.1 | -2.2 | 25.6 | -1.2 | 2.2 | -0.6 | 13.8 | -0.3 |
| | | SLH_low | 25.8 | -2.0 | 147.8 | -0.1 | 2.6 | -37.6 | 18.1 | -30.1 | 2.1 | -8.6 | 13.0 | -6.3 |
| | | SLH_high | 33.3 | 26.1 | 192.0 | 29.8 | 4.1 | -2.2 | 25.0 | -3.6 | 2.8 | 24.3 | 16.5 | 19.2 |
| | AANE+ANT | AANE+ANT | 39.4 | - | 171.9 | - | 4.4 | - | 25.8 | - | 2.3 | - | 14.5 | - |
| | | CH4_icbc | 39.0 | -1.0 | 174.0 | 1.2 | 4.3 | -1.9 | 25.5 | -1.0 | 2.3 | -0.6 | 14.4 | -0.3 |
| | | SLH_low | 38.8 | -1.3 | 171.9 | 0.0 | 2.8 | -35.6 | 18.2 | -29.5 | 2.1 | -8.3 | 13.6 | -6.1 |
| | | SLH_high | 46.2 | 17.4 | 216.1 | 25.7 | 4.3 | -2.2 | 24.9 | -3.3 | 2.9 | 23.4 | 17.1 | 18.4 |
| RCP 6.0 | AANE | AANE | 10.5 | - | 88.2 | - | 3.2 | - | 24.3 | - | 2.2 | - | 13.9 | - |
| | | CH4_icbc | 11.3 | 6.9 | 90.8 | 2.9 | 3.3 | 2.1 | 24.4 | 0.4 | 2.3 | 2.4 | 14.0 | 1.2 |
| | | SLH_low | 10.1 | -3.8 | 87.7 | -0.6 | 2.2 | -30.7 | 18.5 | -23.7 | 2.0 | -8.2 | 13.0 | -5.9 |
| | | SLH_high | 12.8 | 21.3 | 113.3 | 28.4 | 3.2 | -2.0 | 23.3 | -3.9 | 2.7 | 22.4 | 16.3 | 17.5 |
| | AANE+ANT | AANE+ANT | 23.0 | - | 106.9 | - | 3.2 | - | 23.9 | - | 2.2 | - | 14.1 | - |
| | | CH4_icbc | 23.8 | 3.1 | 108.9 | 1.9 | 3.4 | 6.7 | 24.2 | 1.5 | 2.3 | 5.0 | 14.6 | 3.8 |
| | | SLH_low | 22.7 | -1.7 | 106.3 | -0.6 | 2.3 | -30.2 | 18.3 | -23.5 | 2.0 | -8.2 | 13.2 | -5.9 |
| | | SLH_high | 25.3 | 9.6 | 132.0 | 23.4 | 3.2 | -2.0 | 23.0 | -3.7 | 2.7 | 22.3 | 16.5 | 17.3 |
| RCP 8.5 | AANE | AANE | 17.1 | - | 99.2 | - | 3.4 | - | 23.0 | - | 3.2 | - | 17.3 | - |
| | | CH4_icbc | 17.8 | 3.8 | 102.6 | 3.4 | 3.5 | 2.5 | 23.3 | 1.2 | 3.2 | -0.6 | 17.3 | -0.5 |
| | | SLH_low | 16.8 | -2.1 | 98.7 | -0.5 | 2.4 | -30.0 | 18.2 | -21.0 | 2.9 | -10.0 | 16.0 | -7.8 |
| | | SLH_high | 21.4 | 25.0 | 129.6 | 30.6 | 3.4 | 0.9 | 22.6 | -1.8 | 4.0 | 24.9 | 21.0 | 20.8 |
| | AANE+ANT | AANE+ANT | 26.5 | - | 113.6 | - | 3.4 | - | 22.8 | - | 3.2 | - | 17.6 | - |
| | | CH4_icbc | 30.4 | 14.7 | 120.5 | 6.1 | 3.7 | 7.0 | 23.3 | 2.2 | 3.2 | 1.1 | 17.9 | 1.7 |
| | | SLH_low | 26.2 | -1.3 | 113.0 | -0.5 | 2.4 | -29.6 | 18.0 | -21.1 | 2.9 | -10.0 | 16.2 | -7.7 |
| | | SLH_high | 30.8 | 16.1 | 144.0 | 26.7 | 3.5 | 0.9 | 22.4 | -1.5 | 4.0 | 24.8 | 21.2 | 20.6 |

[$] Total inorganic halogen abundance $X_y$ (with X = Cl, Br, I) is defined in the footnote of Supplementary Table S3. The abundance change (Δvmr and ΔBurden) for each species and sensitivity is computed relative to the benchmark scenario during each period of time for the Natural, AANE (no shading) and AANE+ANT (grey shading) configurations.

[&] Tropospheric burden (Tropo) has been computed considering the chemical tropopause (e.g $O_3$ < 150 ppbv).

**Supplementary Table S6: Global mean abundance of the main short-lived climate forcers for the different sensitivities.**

| SLCF Abundance$ | | | Gases | | | | | | | | Aerosols | | | | | |
|---|---|---|---|---|---|---|---|---|---|---|---|---|---|---|---|---|
| | | | $O_3$ | | | | $CH_4$ | | $H_2O$ | | Sulfate | | SOA | | $NH_4NO_3$ | |
| | | | Tropo | | Strat | | Total | | Strat | | Tropo | | Tropo | | Tropo | |
| Period | Simulation | Sensitivity | Col DU | Δcol % | Col DU | Δcol % | Burden Tg | ΔBurden % | vmr ppmv | Δvmr % | Burden Tg | ΔBurden % | Burden Tg | ΔBurden % | Burden Tg | ΔBurden % |
| Pre-industrial | Natural | Natural | 17.8 | - | 275.3 | - | 1996.9 | - | 5.31 | - | 466.1 | - | 912.2 | - | 18.6 | - |
| | | CH4_icbc | 18.1 | 1.7 | 275.3 | 0.0 | 2253.2 | 12.8 | 5.36 | 1.1 | 476.5 | 2.2 | 909.2 | -0.3 | 19.3 | 3.5 |
| | | SLH_low | 18.1 | 2.0 | 274.8 | -0.2 | 2008.1 | 0.6 | 5.27 | -0.7 | 473.6 | 1.6 | 911.7 | -0.1 | 18.5 | -0.6 |
| | | SLH_high | 17.5 | -1.4 | 274.6 | -0.3 | 2018.6 | 1.1 | 5.27 | -0.7 | 463.8 | -0.5 | 917.8 | 0.6 | 18.1 | -2.7 |
| Present-day | AANE | AANE | 25.2 | - | 259.5 | - | 4974.5 | - | 5.61 | - | 1525.9 | - | 822.9 | - | 104.2 | - |
| | | CH4_icbc | 24.8 | -1.5 | 257.2 | -0.9 | 5047.2 | 1.5 | 5.69 | 1.4 | 1507.1 | -1.2 | 833.2 | 1.3 | 115.2 | 10.5 |
| | | SLH_low | 25.8 | 2.3 | 259.2 | -0.1 | 4977.2 | 0.1 | 5.58 | -0.6 | 1530.6 | 0.3 | 824.9 | 0.3 | 106.1 | 1.9 |
| | | SLH_high | 24.6 | -2.3 | 258.8 | -0.3 | 4998.2 | 0.5 | 5.58 | -0.6 | 1518.8 | -0.5 | 828.3 | 0.7 | 102.9 | -1.3 |
| | AANE+ANT | AANE+ANT | 24.9 | - | 258.9 | - | 4991.0 | - | 5.62 | - | 1526.4 | - | 824.8 | - | 105.2 | - |
| | | CH4_icbc | 24.5 | -1.5 | 256.7 | -0.9 | 5052.7 | 1.2 | 5.69 | 1.4 | 1507.7 | -1.2 | 835.0 | 1.2 | 116.4 | 10.6 |
| | | SLH_low | 25.4 | 2.2 | 258.5 | -0.1 | 4994.6 | 0.1 | 5.58 | -0.6 | 1530.9 | 0.3 | 827.0 | 0.3 | 107.2 | 1.9 |
| | | SLH_high | 24.3 | -2.3 | 258.2 | -0.3 | 5015.0 | 0.5 | 5.58 | -0.6 | 1519.3 | -0.5 | 830.0 | 0.6 | 103.7 | -1.5 |
| RCP 6.0 | AANE | AANE | 23.4 | - | 261.4 | - | 4784.6 | - | 5.60 | - | 673.3 | - | 755.6 | - | 223.2 | - |
| | | CH4_icbc | 23.8 | 2.0 | 262.5 | 0.4 | 5073.3 | 6.0 | 5.73 | 2.3 | 711.4 | 5.7 | 773.7 | 2.4 | 227.8 | 2.1 |
| | | SLH_low | 23.9 | 2.2 | 261.0 | -0.2 | 4829.7 | 0.9 | 5.57 | -0.5 | 677.5 | 0.6 | 757.0 | 0.2 | 227.3 | 1.8 |
| | | SLH_high | 23.0 | -1.7 | 260.8 | -0.2 | 4849.2 | 1.3 | 5.57 | -0.5 | 669.0 | -0.6 | 759.8 | 0.6 | 221.9 | -0.6 |
| | AANE+ANT | AANE+ANT | 23.2 | - | 261.2 | - | 4793.3 | - | 5.60 | - | 673.3 | - | 756.3 | - | 225.2 | - |
| | | CH4_icbc | 23.6 | 1.5 | 262.2 | 0.4 | 5080.2 | 6.0 | 5.73 | 2.3 | 711.2 | 5.6 | 775.0 | 2.5 | 230.4 | 2.3 |
| | | SLH_low | 23.7 | 2.1 | 260.8 | -0.2 | 4838.4 | 0.9 | 5.57 | -0.5 | 677.5 | 0.6 | 758.1 | 0.2 | 229.3 | 1.9 |
| | | SLH_high | 22.8 | -1.7 | 260.6 | -0.3 | 4857.6 | 1.3 | 5.57 | -0.5 | 669.0 | -0.6 | 760.7 | 0.6 | 223.7 | -0.7 |
| RCP 8.5 | AANE | AANE | 31.3 | - | 264.1 | - | 14462.0 | - | 6.55 | - | 749.7 | - | 689.0 | - | 259.4 | - |
| | | CH4_icbc | 31.0 | -1.1 | 264.8 | 0.3 | 13566.6 | -6.2 | 6.56 | 0.1 | 785.2 | 4.7 | 710.0 | 3.0 | 251.7 | -3.0 |
| | | SLH_low | 31.8 | 1.6 | 263.7 | -0.2 | 14300.0 | -1.1 | 6.50 | -0.8 | 751.8 | 0.3 | 690.2 | 0.2 | 261.9 | 1.0 |
| | | SLH_high | 30.6 | -2.3 | 263.4 | -0.3 | 14332.6 | -0.9 | 6.50 | -0.7 | 744.7 | -0.7 | 692.5 | 0.5 | 254.1 | -2.0 |
| | AANE+ANT | AANE+ANT | 31.2 | - | 264.0 | - | 14467.5 | - | 6.55 | - | 749.7 | - | 689.6 | - | 261.7 | - |
| | | CH4_icbc | 30.8 | -1.5 | 264.6 | 0.2 | 13572.4 | -6.2 | 6.56 | 0.1 | 785.1 | 4.7 | 711.0 | 3.1 | 254.8 | -2.6 |
| | | SLH_low | 31.7 | 1.6 | 263.6 | -0.2 | 14305.3 | -1.1 | 6.51 | -0.8 | 752.0 | 0.3 | 691.2 | 0.2 | 264.2 | 0.9 |
| | | SLH_high | 30.5 | -2.3 | 263.2 | -0.3 | 14337.8 | -0.9 | 6.51 | -0.7 | 744.9 | -0.6 | 693.2 | 0.5 | 256.1 | -2.2 |

$ The abundance change for each species and sensitivity is computed relative to the benchmark scenario. Tropo column considers the chemical tropopause (e.g $O_3$ < 150 ppbv).

**Supplementary Table S7: Global mean SLH-driven radiative effect for the full set of sensitivities performed for present-day conditions.**

| Species | | Gases | | | | | | Aerosols | | | | | | Total | | | | | |
|---|---|---|---|---|---|---|---|---|---|---|---|---|---|---|---|---|---|---|---|
| | | Ozone | | Methane | | $H_2O^{strat}$ | | Ammonium | | Sulfate | | SOA | | Gases | | Aerosols | | GAS+AER | |
| Period | Simulation | mean | 2σ | mean | 2σ | mean | 2σ | mean | 2σ | mean | 2σ | mean | 2σ | mean | 2σ | mean | 2σ | mean | 2σ |
| | | W m$^{-2}$ | | W m$^{-2}$ | | W m$^{-2}$ | | W m$^{-2}$ | | W m$^{-2}$ | | W m$^{-2}$ | | W m$^{-2}$ | | W m$^{-2}$ | | W m$^{-2}$ | |
| Present-day AANE (Sensitivities) | **AANE** | **-0.218** | **0.009** | **0.093** | **5.E-03** | **0.012** | **6.E-04** | **4.1E-03** | **2.E-04** | **0.031** | **0.001** | **-1.6E-03** | **1.E-04** | **-0.146** | **0.010** | **0.034** | **0.002** | **-0.112** | **0.010** |
| | **CH4_icbc** | **-0.232** | **0.012** | **0.077** | **0.015** | **0.010** | **2.E-03** | **4.5E-03** | **4.E-04** | **0.030** | **0.001** | **-1.4E-03** | **1.E-03** | **-0.165** | **0.022** | **0.035** | **0.004** | **-0.130** | **0.020** |
| | **SLH_low** | **-0.206** | **0.008** | **0.089** | **4.E-04** | **0.011** | **5.E-05** | **4.1E-03** | **3.E-04** | **0.026** | **0.002** | **-4.8E-05** | **2.E-04** | **-0.135** | **0.007** | **0.030** | **0.002** | **-0.106** | **0.007** |
| | **SLH_high** | **-0.239** | **0.009** | **0.094** | **0.006** | **0.012** | **8.E-04** | **5.2E-03** | **5.E-04** | **0.032** | **0.002** | **6.5E-06** | **2.E-04** | **-0.166** | **0.009** | **0.037** | **0.003** | **-0.128** | **0.010** |
| | low_I | -0.213 | 0.008 | 0.090 | 0.002 | 0.011 | 2.E-04 | 4.1E-03 | 2.E-04 | 0.031 | 0.002 | 5.8E-06 | 1.E-04 | -0.142 | 0.011 | 0.034 | 0.002 | -0.107 | 0.011 |
| | high_I | -0.228 | 0.011 | 0.092 | 0.006 | 0.012 | 8.E-04 | 4.4E-03 | 1.E-03 | 0.031 | 0.003 | -3.3E-04 | 2.E-03 | -0.255 | 0.428 | 0.051 | 0.069 | -0.203 | 0.360 |
| | low_Br | -0.211 | 0.008 | 0.089 | 0.001 | 0.011 | 1.E-04 | 4.1E-03 | 3.E-04 | 0.027 | 0.002 | 6.4E-06 | 2.E-04 | -0.142 | 0.014 | 0.031 | 0.002 | -0.110 | 0.013 |
| | high_Cl | -0.226 | 0.008 | 0.093 | 0.005 | 0.012 | 7.E-04 | 5.2E-03 | 4.E-04 | 0.031 | 0.002 | -5.7E-06 | 1.E-04 | -0.153 | 0.008 | 0.036 | 0.002 | -0.116 | 0.009 |
| | polar_OFF | -0.218 | 0.008 | 0.090 | 0.002 | 0.011 | 2.E-04 | 4.1E-03 | 3.E-04 | 0.024 | 0.003 | 4.6E-05 | 1.E-04 | -0.151 | 0.009 | 0.029 | 0.004 | -0.122 | 0.008 |
| | aerchem_OFF | -0.217 | 0.008 | 0.087 | 0.002 | 0.011 | 3.E-04 | 4.3E-03 | 3.E-04 | 0.025 | 0.002 | 1.7E-05 | 5.E-03 | -0.148 | 0.012 | 0.044 | 0.007 | -0.103 | 0.012 |
| Present-day AANE (Range) | min | -0.239 | | 0.077 | | 0.010 | | 4.1E-03 | | 0.024 | | -1.6E-03 | | -0.255 | | 0.029 | | -0.203 | |
| | max | -0.206 | | 0.094 | | 0.012 | | 5.2E-03 | | 0.032 | | 4.6E-05 | | -0.135 | | 0.051 | | -0.103 | |
| | range[&] | 0.017 | | 0.008 | | 0.001 | | 0.001 | | 0.004 | | 0.001 | | 0.060 | | 0.011 | | 0.050 | |
| Present-day AANE+ANT (Sensitivities) | **AANE+ANT** | **-0.237** | **0.009** | **0.097** | **5.E-03** | **0.012** | **6.E-04** | **3.7E-03** | **2.E-04** | **0.030** | **0.001** | **-1.8E-03** | **1.E-04** | **-0.160** | **0.012** | **0.034** | **0.002** | **-0.126** | **0.013** |
| | **CH4_icbc** | **-0.249** | **0.013** | **0.078** | **0.016** | **0.010** | **2.E-03** | **4.1E-03** | **3.E-04** | **0.030** | **0.002** | **-1.6E-03** | **1.E-03** | **-0.182** | **0.021** | **0.034** | **0.003** | **-0.147** | **0.019** |
| | **SLH_low** | **-0.224** | **0.008** | **0.093** | **3.E-04** | **0.012** | **4.E-05** | **3.7E-03** | **3.E-04** | **0.026** | **0.002** | **-5.4E-06** | **1.E-04** | **-0.153** | **0.011** | **0.030** | **0.002** | **-0.123** | **0.010** |
| | **SLH_high** | **-0.258** | **0.010** | **0.098** | **0.006** | **0.012** | **7.E-04** | **4.9E-03** | **4.E-04** | **0.032** | **0.002** | **9.1E-06** | **1.E-04** | **-0.182** | **0.009** | **0.037** | **0.002** | **-0.144** | **0.009** |
| | low_I | -0.231 | 0.009 | 0.094 | 0.002 | 0.012 | 2.E-04 | 3.7E-03 | 2.E-04 | 0.030 | 0.002 | -8.8E-06 | 1.E-04 | -0.158 | 0.009 | 0.034 | 0.003 | -0.124 | 0.009 |
| | high_I | -0.249 | 0.010 | 0.095 | 0.003 | 0.012 | 3.E-04 | 3.8E-03 | 2.E-04 | 0.031 | 0.002 | -4.2E-05 | 2.E-04 | -0.173 | 0.013 | 0.035 | 0.003 | -0.138 | 0.015 |
| | low_Br | -0.230 | 0.008 | 0.093 | 0.001 | 0.012 | 7.E-05 | 3.7E-03 | 2.E-04 | 0.026 | 0.002 | -1.1E-05 | 2.E-04 | -0.156 | 0.007 | 0.030 | 0.002 | -0.125 | 0.009 |
| | high_Cl | -0.245 | 0.009 | 0.097 | 0.005 | 0.012 | 6.E-04 | 4.9E-03 | 3.E-04 | 0.031 | 0.002 | -5.5E-05 | 1.E-04 | -0.167 | 0.005 | 0.036 | 0.003 | -0.131 | 0.003 |
| | polar_OFF | -0.237 | 0.009 | 0.094 | 0.002 | 0.012 | 2.E-04 | 3.7E-03 | 3.E-04 | 0.024 | 0.003 | -3.1E-05 | 2.E-04 | -0.163 | 0.010 | 0.028 | 0.003 | -0.135 | 0.012 |
| | aerchem_OFF | -0.239 | 0.009 | 0.090 | 0.003 | 0.011 | 3.E-04 | 4.0E-03 | 3.E-04 | 0.025 | 0.002 | 9.4E-06 | 5.E-03 | -0.167 | 0.008 | 0.045 | 0.006 | -0.122 | 0.009 |
| Present-day AANE+ANT (Range) | min | -0.258 | | 0.078 | | 0.010 | | 3.7E-03 | | 0.024 | | -1.8E-03 | | -0.182 | | 0.028 | | -0.147 | |
| | max | -0.224 | | 0.098 | | 0.012 | | 4.9E-03 | | 0.032 | | 9.4E-06 | | -0.153 | | 0.045 | | -0.122 | |
| | range[&] | 0.017 | | 0.010 | | 0.001 | | 0.001 | | 0.004 | | 0.001 | | 0.015 | | 0.008 | | 0.013 | |

[&] mean RE values are expressed as the multi-year average for each of the benchmark AANE (no shading) and AANE+ANT (grey shading) configurations, while range is computed as half of the difference between the maximum and minimum RE obtained for the complete set of model sensitivities (expressed as mean ± range/2) during each period of time. Lines highlighted in bold are for the main present-day sensitivities as described in Supplementary Text ST1.

**Supplementary Table S8: Global mean SLH-driven radiative effect for the main sensitivities performed during pre-industrial and present-day.**

| Species | | | Gases | | | | | | Aerosols | | | | | | Total | | | | | |
|---|---|---|---|---|---|---|---|---|---|---|---|---|---|---|---|---|---|---|---|---|
| | | | Ozone | | Methane | | $H_2O^{strat}$ | | Ammonium | | Sulfate | | SOA | | Gases | | Aerosols | | GAS+AER | |
| Period | Simulation | Sensitivity | mean | 2σ | mean | 2σ | mean | 2σ | mean | 2σ | mean | 2σ | mean | 2σ | mean | 2σ | mean | 2σ | mean | 2σ |
| | | | W m$^{-2}$ | | W m$^{-2}$ | | W m$^{-2}$ | | W m$^{-2}$ | | W m$^{-2}$ | | W m$^{-2}$ | | W m$^{-2}$ | | W m$^{-2}$ | | W m$^{-2}$ | |
| Pre-Industrial | Natural | Natural | -0.164 | 0.006 | 0.090 | 5.E-04 | 0.011 | 6.E-05 | 3.5E-04 | 6.E-05 | 0.036 | 0.001 | -3.0E-03 | 3.E-04 | -0.113 | 0.011 | 0.034 | 0.002 | -0.078 | 0.011 |
| | | CH4_icbc | -0.167 | 0.011 | 0.087 | 7.E-04 | 0.011 | 9.E-05 | 3.8E-04 | 7.E-05 | 0.035 | 0.002 | -2.9E-03 | 3.E-04 | -0.115 | 0.018 | 0.034 | 0.002 | -0.081 | 0.017 |
| | | SLH_low | -0.156 | 0.004 | 0.089 | 2.E-03 | 0.011 | 2.E-04 | 3.9E-04 | 3.E-05 | 0.032 | 0.002 | -2.6E-03 | 3.E-04 | -0.103 | 0.012 | 0.030 | 0.002 | -0.073 | 0.013 |
| | | SLH_high | -0.173 | 0.003 | 0.093 | 3.E-03 | 0.012 | 4.E-04 | 5.5E-04 | 4.E-05 | 0.037 | 0.002 | -3.4E-03 | 3.E-04 | -0.119 | 0.017 | 0.035 | 0.003 | -0.083 | 0.017 |
| | | **RE±range[&]** | **-0.164** | **0.009** | **0.089** | **0.003** | **0.011** | **4.E-04** | **3.5E-04** | **1.E-04** | **0.036** | **0.003** | **-0.003** | **4.E-04** | **-0.113** | **0.008** | **0.034** | **0.002** | **-0.078** | **0.005** |
| Present-day | AANE | AANE | -0.218 | 0.009 | 0.093 | 5.E-03 | 0.012 | 6.E-04 | 4.1E-03 | 2.E-04 | 0.031 | 0.001 | -1.6E-03 | 1.E-04 | -0.146 | 0.010 | 0.034 | 0.002 | -0.112 | 0.010 |
| | | CH4_icbc | -0.232 | 0.012 | 0.077 | 2.E-02 | 0.010 | 2.E-03 | 4.5E-03 | 4.E-04 | 0.030 | 0.001 | -1.4E-03 | 1.E-03 | -0.165 | 0.022 | 0.035 | 0.004 | -0.130 | 0.020 |
| | | SLH_low | -0.206 | 0.008 | 0.089 | 4.E-04 | 0.011 | 5.E-05 | 4.1E-03 | 3.E-04 | 0.026 | 0.002 | -1.4E-03 | 2.E-04 | -0.135 | 0.007 | 0.030 | 0.002 | -0.106 | 0.007 |
| | | SLH_high | -0.239 | 0.009 | 0.094 | 6.E-03 | 0.012 | 8.E-04 | 5.2E-03 | 5.E-04 | 0.032 | 0.002 | -1.8E-03 | 2.E-04 | -0.166 | 0.009 | 0.037 | 0.003 | -0.128 | 0.010 |
| | | **RE±range[&]** | **-0.218** | **0.017** | **0.085** | **0.008** | **0.011** | **1.E-03** | **4.1E-03** | **6.E-04** | **0.031** | **0.003** | **-1.6E-03** | **2.E-04** | **-0.146** | **0.015** | **0.034** | **0.004** | **-0.112** | **0.012** |
| | AANE+ANT | AANE+ANT | -0.237 | 0.009 | 0.097 | 5.E-03 | 0.012 | 6.E-04 | 3.7E-03 | 2.E-04 | 0.030 | 0.001 | -1.8E-03 | 1.E-04 | -0.160 | 0.012 | 0.034 | 0.002 | -0.126 | 0.013 |
| | | CH4_icbc | -0.249 | 0.013 | 0.078 | 2.E-02 | 0.010 | 2.E-03 | 4.1E-03 | 3.E-04 | 0.030 | 0.002 | -1.6E-03 | 1.E-03 | -0.182 | 0.021 | 0.034 | 0.003 | -0.147 | 0.019 |
| | | SLH_low | -0.224 | 0.008 | 0.093 | 3.E-04 | 0.012 | 4.E-05 | 3.7E-03 | 3.E-04 | 0.026 | 0.002 | -1.7E-03 | 1.E-04 | -0.153 | 0.011 | 0.030 | 0.002 | -0.123 | 0.010 |
| | | SLH_high | -0.258 | 0.010 | 0.098 | 6.E-03 | 0.012 | 7.E-04 | 4.9E-03 | 4.E-04 | 0.032 | 0.002 | -2.0E-03 | 1.E-04 | -0.182 | 0.009 | 0.037 | 0.002 | -0.144 | 0.009 |
| | | **RE±range[&]** | **-0.237** | **0.017** | **0.088** | **0.010** | **0.011** | **1.E-03** | **3.7E-03** | **6.E-04** | **0.030** | **0.003** | **-1.8E-03** | **2.E-04** | **-0.160** | **0.015** | **0.034** | **0.004** | **-0.126** | **0.012** |

[&] mean RE values are expressed as the multi-year average for each of the benchmark Natural, AANE (no shading) and AANE+ANT (grey shading) configurations, while range is computed as half of the difference between the maximum and minimum RE obtained for the complete set of model sensitivities (expressed as mean ± range/2) during each period of time. The mean RE ± range lines highlighted in bold are summarized in EDT5.

**Supplementary Table S9: Global mean SLH-driven radiative effect for the main sensitivities performed for future climate scenarios.**

| Species | | | Gases | | | | | | Aerosols | | | | | | Total | | | | | |
|---|---|---|---|---|---|---|---|---|---|---|---|---|---|---|---|---|---|---|---|---|
| | | | Ozone | | Methane | | $H_2O^{strat}$ | | Ammonium | | Sulfate | | SOA | | Gases | | Aerosols | | GAS+AER | |
| Period | Simulation | Sensitivity | mean | 2σ | mean | 2σ | mean | 2σ | mean | 2σ | mean | 2σ | mean | 2σ | mean | 2σ | mean | 2σ | mean | 2σ |
| | | | $W\ m^{-2}$ | | $W\ m^{-2}$ | | $W\ m^{-2}$ | | $W\ m^{-2}$ | | $W\ m^{-2}$ | | $W\ m^{-2}$ | | $W\ m^{-2}$ | | $W\ m^{-2}$ | | $W\ m^{-2}$ | |
| RCP 6.0 | AANE | AANE | -0.189 | 0.007 | 0.105 | 3.E-03 | 0.013 | 4.E-04 | 5.0E-03 | 4.E-04 | 0.031 | 0.001 | -1.3E-03 | 2.E-04 | -0.118 | 0.013 | 0.035 | 0.002 | -0.083 | 0.014 |
| | | CH4_icbc | -0.201 | 0.017 | 0.096 | 1.E-02 | 0.012 | 1.E-03 | 5.6E-03 | 1.E-03 | 0.031 | 0.001 | -1.4E-03 | 1.E-03 | -0.131 | 0.021 | 0.036 | 0.004 | -0.095 | 0.018 |
| | | SLH_low | -0.180 | 0.003 | 0.102 | 3.E-04 | 0.013 | 4.E-05 | 5.0E-03 | 4.E-04 | 0.027 | 0.002 | -1.2E-03 | 2.E-04 | -0.110 | 0.010 | 0.032 | 0.001 | -0.078 | 0.009 |
| | | SLH_high | -0.206 | 0.005 | 0.107 | 5.E-03 | 0.013 | 7.E-04 | 6.8E-03 | 5.E-04 | 0.032 | 0.002 | -1.5E-03 | 3.E-04 | -0.130 | 0.013 | 0.038 | 0.001 | -0.091 | 0.013 |
| | | **RE±range[&]** | **-0.189** | **0.013** | **0.101** | **0.006** | **0.013** | **7.E-04** | **5.0E-03** | **9.E-04** | **0.031** | **0.002** | **-1.3E-03** | **2.E-04** | **-0.118** | **0.011** | **0.035** | **0.003** | **-0.083** | **0.009** |
| | AANE+ANT | AANE+ANT | -0.195 | 0.007 | 0.107 | 3.E-03 | 0.013 | 4.E-04 | 4.3E-03 | 4.E-04 | 0.031 | 0.001 | -1.4E-03 | 2.E-04 | -0.122 | 0.013 | 0.035 | 0.002 | -0.087 | 0.012 |
| | | CH4_icbc | -0.210 | 0.016 | 0.097 | 1.E-02 | 0.012 | 1.E-03 | 4.6E-03 | 1.E-03 | 0.031 | 0.001 | -1.6E-03 | 1.E-03 | -0.139 | 0.022 | 0.035 | 0.004 | -0.103 | 0.020 |
| | | SLH_low | -0.186 | 0.003 | 0.104 | 3.E-04 | 0.013 | 4.E-05 | 4.3E-03 | 5.E-04 | 0.028 | 0.002 | -1.3E-03 | 3.E-04 | -0.114 | 0.007 | 0.031 | 0.002 | -0.082 | 0.006 |
| | | SLH_high | -0.212 | 0.005 | 0.109 | 5.E-03 | 0.014 | 7.E-04 | 6.2E-03 | 6.E-04 | 0.032 | 0.002 | -1.6E-03 | 3.E-04 | -0.136 | 0.007 | 0.038 | 0.001 | -0.098 | 0.009 |
| | | **RE±range[&]** | **-0.195** | **0.013** | **0.102** | **0.006** | **0.013** | **7.E-04** | **4.3E-03** | **1.E-03** | **0.031** | **0.002** | **-1.4E-03** | **2.E-04** | **-0.122** | **0.012** | **0.035** | **0.003** | **-0.087** | **0.011** |
| RCP 8.5 | AANE | AANE | -0.235 | 0.009 | 0.113 | 7.E-03 | 0.014 | 9.E-04 | 8.6E-03 | 5.E-04 | 0.028 | 0.001 | -1.0E-03 | 1.E-04 | -0.134 | 0.013 | 0.035 | 0.002 | -0.098 | 0.012 |
| | | CH4_icbc | -0.243 | 0.018 | 0.100 | 1.E-02 | 0.013 | 1.E-03 | 8.5E-03 | 7.E-04 | 0.028 | 0.002 | -1.0E-03 | 2.E-04 | -0.149 | 0.025 | 0.035 | 0.002 | -0.114 | 0.026 |
| | | SLH_low | -0.258 | 0.007 | 0.112 | 6.E-03 | 0.014 | 8.E-04 | 1.1E-02 | 7.E-04 | 0.029 | 0.002 | -1.2E-03 | 2.E-04 | -0.155 | 0.006 | 0.039 | 0.003 | -0.116 | 0.009 |
| | | SLH_high | -0.224 | 0.003 | 0.108 | 2.E-03 | 0.013 | 2.E-04 | 8.6E-03 | 5.E-04 | 0.025 | 0.002 | -9.6E-04 | 2.E-04 | -0.127 | 0.004 | 0.033 | 0.002 | -0.094 | 0.005 |
| | | **RE±range[&]** | **-0.235** | **0.017** | **0.107** | **0.006** | **0.013** | **8.E-04** | **8.6E-03** | **1.E-03** | **0.028** | **0.002** | **-1.0E-03** | **1.E-04** | **-0.134** | **0.014** | **0.035** | **0.003** | **-0.098** | **0.011** |
| | AANE+ANT | AANE+ANT | -0.240 | 0.009 | 0.113 | 7.E-03 | 0.014 | 9.E-04 | 7.8E-03 | 4.E-04 | 0.028 | 0.001 | -1.1E-03 | 1.E-04 | -0.137 | 0.015 | 0.035 | 0.002 | -0.103 | 0.015 |
| | | CH4_icbc | -0.253 | 0.016 | 0.101 | 1.E-02 | 0.013 | 1.E-03 | 7.3E-03 | 7.E-04 | 0.028 | 0.002 | -1.1E-03 | 2.E-04 | -0.159 | 0.027 | 0.034 | 0.002 | -0.125 | 0.028 |
| | | SLH_low | -0.229 | 0.003 | 0.108 | 2.E-03 | 0.014 | 2.E-04 | 7.8E-03 | 5.E-04 | 0.025 | 0.002 | -1.0E-03 | 2.E-04 | -0.130 | 0.007 | 0.032 | 0.003 | -0.099 | 0.009 |
| | | SLH_high | -0.263 | 0.007 | 0.112 | 6.E-03 | 0.014 | 8.E-04 | 1.1E-02 | 7.E-04 | 0.029 | 0.002 | -1.3E-03 | 2.E-04 | -0.158 | 0.007 | 0.038 | 0.003 | -0.120 | 0.008 |
| | | **RE±range[&]** | **-0.240** | **0.017** | **0.107** | **0.006** | **0.013** | **8.E-04** | **7.8E-03** | **2.E-03** | **0.028** | **0.002** | **-1.1E-03** | **1.E-04** | **-0.137** | **0.014** | **0.035** | **0.003** | **-0.103** | **0.013** |

[&] mean RE values are expressed as the multi-year average for each of the benchmark Natural, AANE (no shading) and AANE+ANT (grey shading) configurations, while range is computed as half of the difference between the maximum and minimum RE obtained for the complete set of model sensitivities (expressed as mean ± range/2) during each period of time. The mean RE ± range lines highlighted in bold are summarized in EDT5.

**Supplementary Table S10. Comparison of ozone observations and CESM/CAM-Chem for the pre-industrial period.**

| Location | | Observations[&] | | Model[$] | | Statistics[#] (Natural) | | | Statistics[#] (NoSLH) | | |
|---|---|---|---|---|---|---|---|---|---|---|---|
| Place | (lat,lon) | Years | Obs. ppbv | Natural ppbv | NoSLH ppbv | Bias ppbv | NMB a.u. | MFE % | Bias ppbv | NMB a.u. | MFE % |
| Montsouris | (49.03,2.31) | 1876-1886 | 9.56 | 17.26 | 22.53 | 7.70 | 0.81 | 56.87 | 12.98 | 1.36 | 80.22 |
| Vienna | (48.21,16.37) | 1891-1895 | 12.25 | 18.13 | 22.90 | 5.88 | 0.48 | 39.54 | 10.64 | 0.87 | 61.02 |
| Mont Ventoux | (44.17,5.28) | 1891-1903 | 12.88 | 18.90 | 24.15 | 6.02 | 0.47 | 37.01 | 11.26 | 0.87 | 60.31 |
| Pic du Midi | (42.94,-0.14) | 1874-1884 | 10.75 | 18.65 | 24.05 | 7.90 | 0.73 | 53.01 | 13.30 | 1.24 | 76.08 |
| Coimbra | (40.21,-8.42) | 1890-1895 | 8.00 | 17.68 | 23.13 | 9.68 | 1.21 | 74.57 | 15.13 | 1.89 | 97.39 |
| Tokyo | (35.69,139.69) | 1895-1901 | 14.42 | 15.40 | 20.35 | 0.98 | 0.07 | 34.55 | 5.93 | 0.41 | 40.92 |
| Hong Kong | (22.21,114.16) | 1872-1874 | 7.97 | 12.97 | 16.19 | 5.01 | 0.63 | 40.82 | 8.22 | 1.03 | 60.67 |
| Luanda | (-8.84,13.23) | 1890-1895 | 6.19 | 13.04 | 16.69 | 6.85 | 1.11 | 54.67 | 10.50 | 1.70 | 80.60 |
| Mauritius | (-20.17,57.33) | 1898-1909 | 10.51 | 11.53 | 16.43 | 1.01 | 0.10 | 19.93 | 5.92 | 0.56 | 39.09 |
| Montevideo | (-34.88,-56.17) | 1918-1928 | 12.24 | 13.70 | 17.46 | 1.47 | 0.12 | 17.21 | 5.22 | 0.43 | 34.08 |
| Adelaide | (-34.93,-138.6) | 1880-1907 | 11.52 | 13.27 | 18.72 | 1.75 | 0.15 | 43.27 | 7.20 | 0.62 | 45.67 |
| Hobart | (-42.88, 147.33) | 1876-1879 | 11.45 | 14.15 | 19.87 | 2.70 | 0.24 | 34.32 | 8.42 | 0.74 | 50.75 |

[&] Observations are based on the compendium of pre-industrial measurements performed all over the world between 1870 and 1910 compiled in Fig. 2 in Mickely et al.[102]. Note that these pre-industrial observations possess large uncertainties due to different interferences and calibration corrections. It should also be noted that most Earth system models tend to overestimate these highly uncertain ozone abundances during pre-industrial periods[102].

[$] Model values were computed as the multi-year mean for the NoSLH and Natural benchmark pre-industrial configurations at the model gridpoint containing the exact location where the observations were performed (model resolution is 1.9º latitude × 2.5º longitude).

[#] NMB = Normalized mean bias (range from −2 to +∞); MFE = Mean fractional error (range from 0 to +200%).

# Supplementary Figures

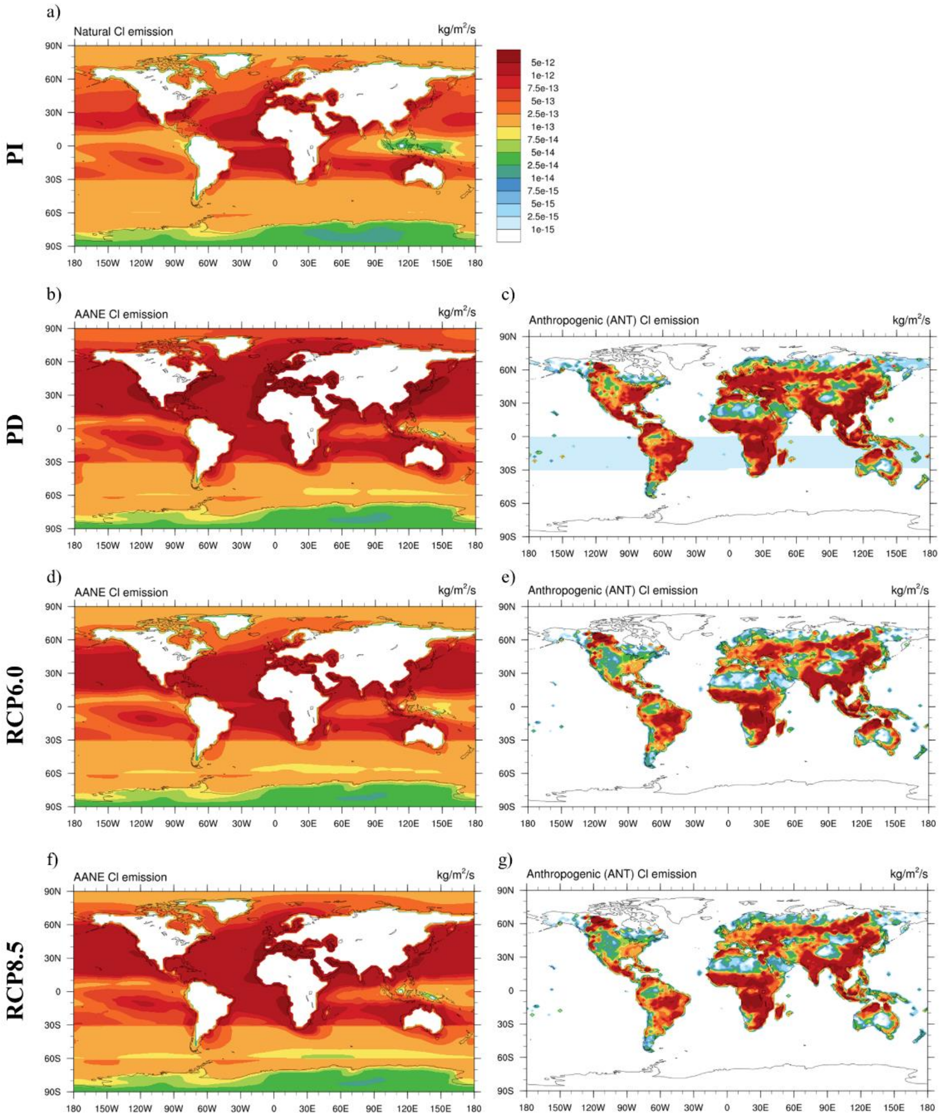


**Supplementary Figure S1. Spatial distribution of global chlorine emissions from natural and anthropogenic sources.** Only natural (panel **a**) source is considered for the pre-industrial (PI) case; while anthropogenically amplified natural emissions (AANE, left column) and pure anthropogenic (ANT, right column) sources are included for present-day (PD, panels **b, c**) and future RCP 6.0 (panels **d, e**) and RCP 8.5 (panels **f, g**) scenarios. All panels have the same emission flux scale and units (kg $m^{-2}$ $s^{-1}$) provided in panel **a**. The tropospheric SSA-chlorine flux is added on top of the surface SLH emissions. A small source of anthropogenic SLH chlorine is present in the tropical region under PD, which results from the inversion model calculation from Claxton et al.[68]. Results for RCP 6.0 and RCP 8.5 projections are for year 2100, while emissions during present-day and pre-industrial periods are representative of year 2020 and 1750, respectively (see EDT1). Panels were created using gsn_csm library for NCL.

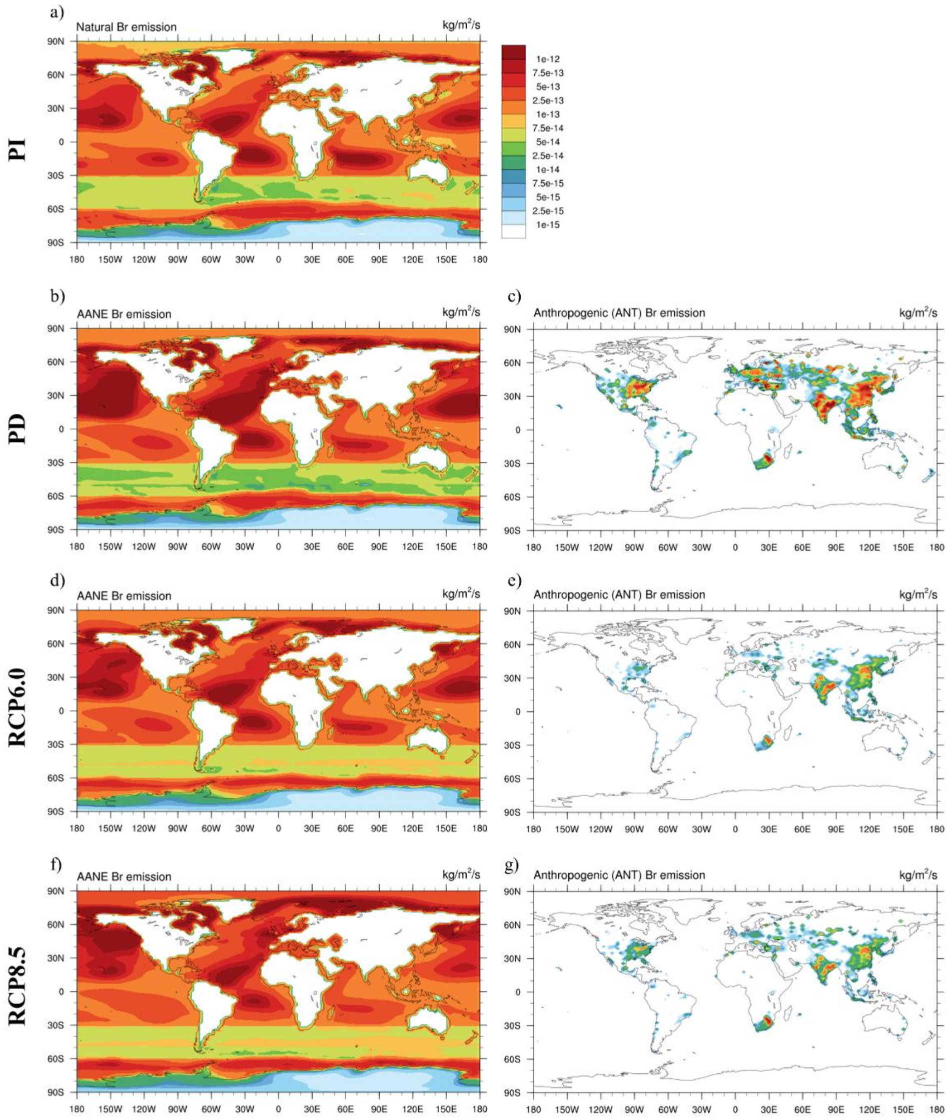


**Supplementary Figure S2. Spatial distribution of global bromine emissions from natural and anthropogenic sources.** Only natural (panel **a**) source is considered for the pre-industrial (PI) case; while anthropogenically amplified natural emissions (AANE, left column) and pure anthropogenic (ANT, right column) sources are included for present-day (PD, panels **b, c**) and future RCP 6.0 (panels **d, e**) and RCP 8.5 (panels **f, g**) scenarios. The tropospheric SSA-bromine flux is added on top of the surface SLH emissions. All panels have the same emission flux scale and units (kg $m^{-2}$ $s^{-1}$) provided in panel **a**. Results for RCP 6.0 and RCP 8.5 projections are for year 2100, while emissions during present-day and pre-industrial periods are representative of year 2020 and 1750, respectively (see EDT1). Panels were created using gsn_csm library for NCL.

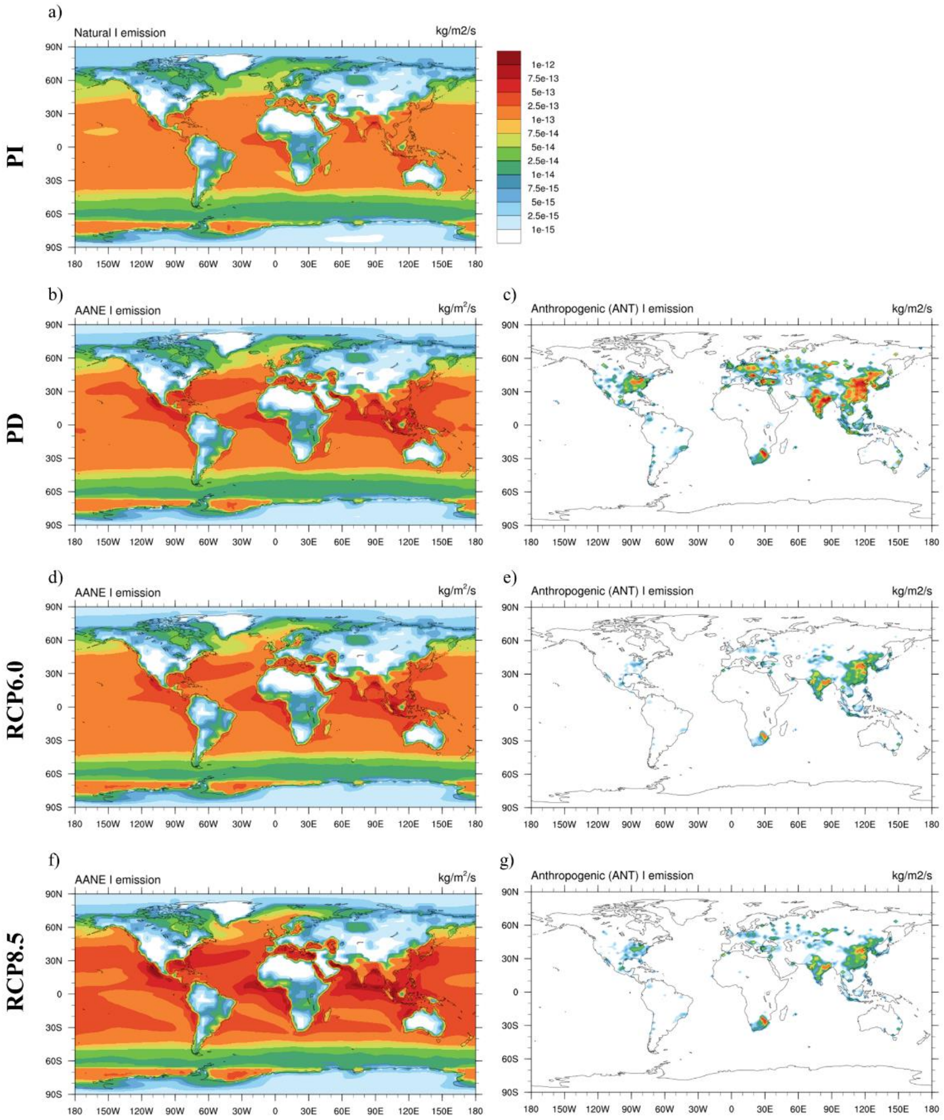


**Supplementary Figure S3. Spatial distribution of global iodine emissions from natural and anthropogenic sources.** Only natural (panel **a**) source is considered for the pre-industrial (PI) case; while anthropogenically amplified natural emissions (AANE, left column) and pure anthropogenic (ANT, right column) sources are included for present-day (PD, panels **b, c**) and future RCP 6.0 (panels **d, e**) and RCP 8.5 (panels **f, g**) scenarios. All panels have the same emission flux scale and units (kg $m^{-2}$ $s^{-1}$) provided in panel **a**. Results for RCP 6.0 and RCP 8.5 projections are for year 2100, while emissions during present-day and pre-industrial periods are representative of year 2020 and 1750, respectively (see EDT1). Panels were created using gsn_csm library for NCL.

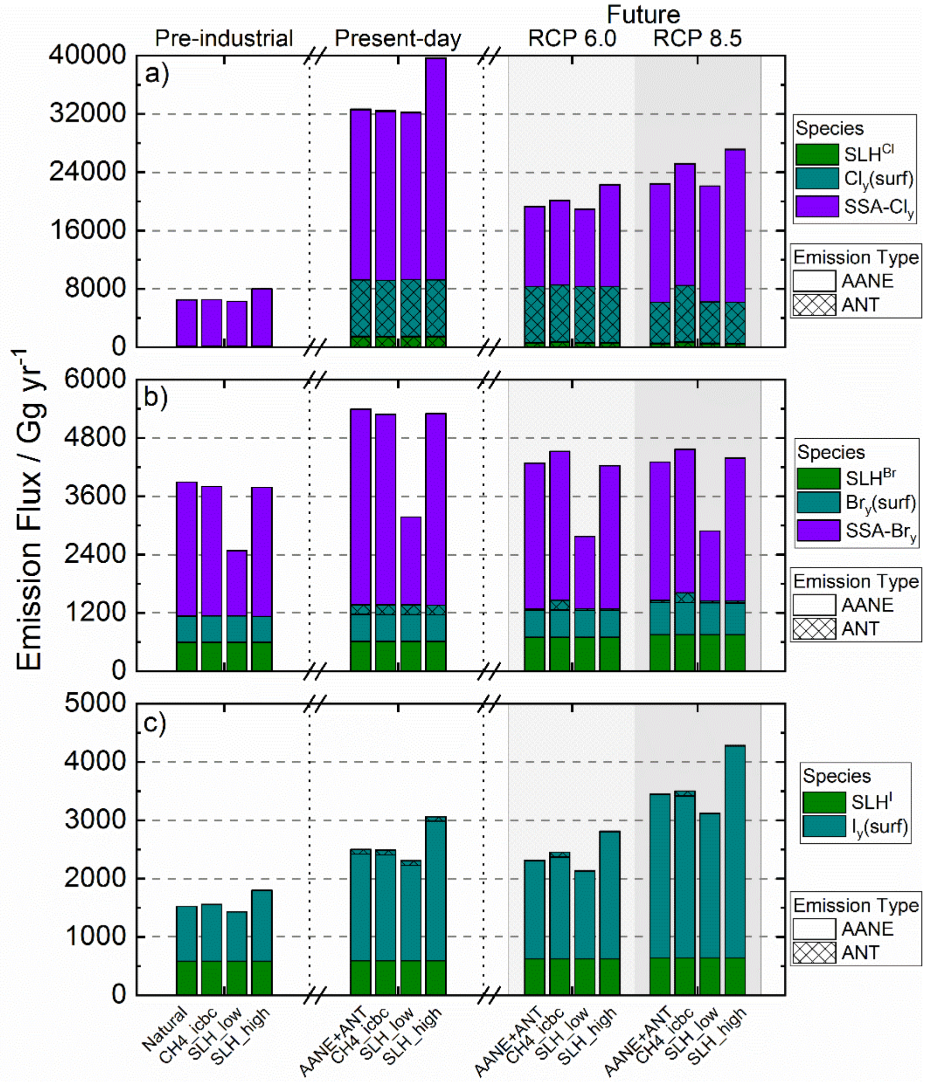


**Supplementary Figure S4. Comparison of natural and anthropogenic global mean SLH annual emissions for the main sensitivity simulations.** The individual contribution arising from SLH-halocarbons, surface inorganic halogens and SSA-dehalogenation for the case of chlorine (**a**), bromine (**b**) and iodine (**c**) are shown, distinguishing the AANE fraction (empty colored bars) with respect to that from ANT (stippled colored bars). Results for the pre-industrial period are on the left of each panel and consider only natural halogen emissions, while the total fluxes in year 2100 for RCP6.0 (light-grey shading) and RCP8.5 (heavy-grey shading) climate scenarios are shown on the right. Results indicate that, with the exception of chlorine, the anthropogenic contribution is negligible compared to the anthropogenically amplified natural emissions, that dominate the SLH flux during all time periods. The spatial distribution of the total sources for each sensitivity is shown in Supplementary Figures S1-S3.

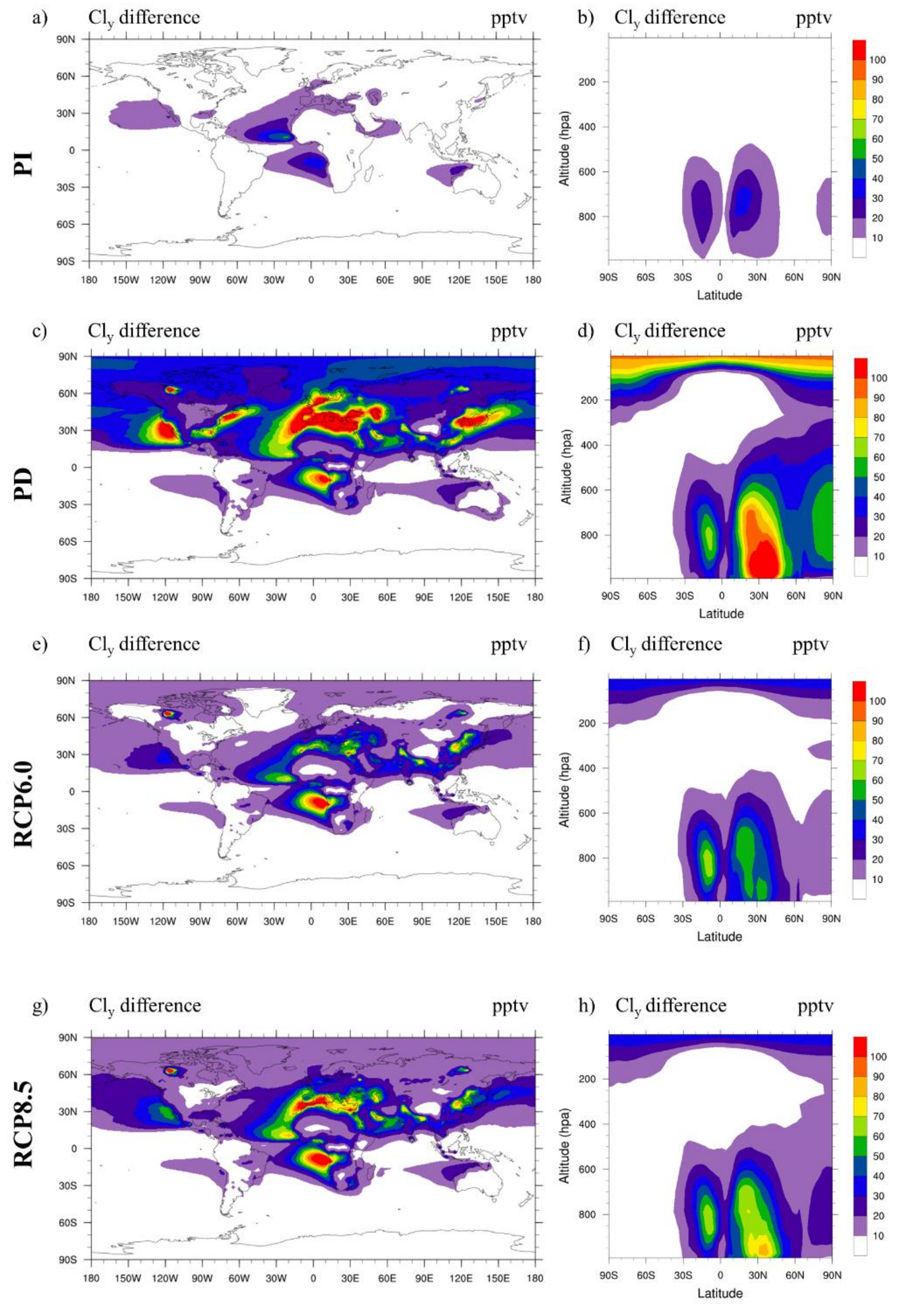


**Supplementary Figure S5. Spatial distribution of the difference in inorganic chlorine species ($Cl_y$) between AANE+ANT and the NoSLH benchmark simulations**. The change in abundance for the annual mean mixing ratio (pptv) vertically averaged within the troposphere (left column) and for the zonal average (middle column) are shown for the pre-industrial case (PI, year 1750, panels **a, b**), present-day (PD, year 2020, panels **c, d**) and future RCP 6.0 (panels **e, f**) and RCP 8.5 (panels **g, h**) scenarios (year 2100). Panels were created using gsn_csm library for NCL.

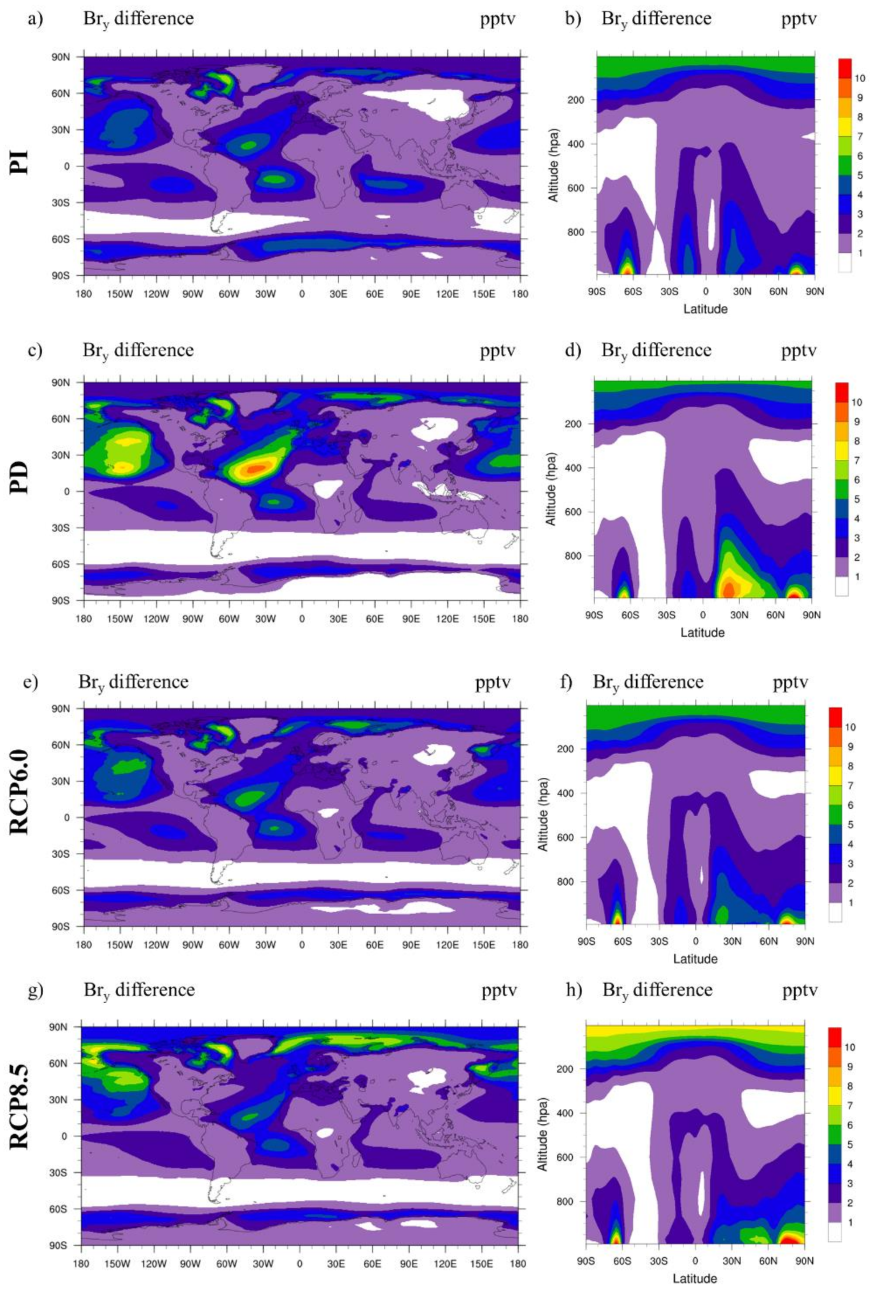


**Supplementary Figure S6. Spatial distribution of the difference in inorganic bromine species ($Br_y$) between AANE+ANT and the NoSLH simulations.** The change in abundance for the annual mean mixing ratio (pptv) vertically averaged within the troposphere (left column) and for the zonal average (middle column) are shown for the pre-industrial case (PI, panels **a, b**), present-day (PD, panels **c, d**) and future RCP 6.0 (panels **e, f**) and RCP 8.5 (panels **g, h**) scenarios. Panels were created using gsn_csm library for NCL.

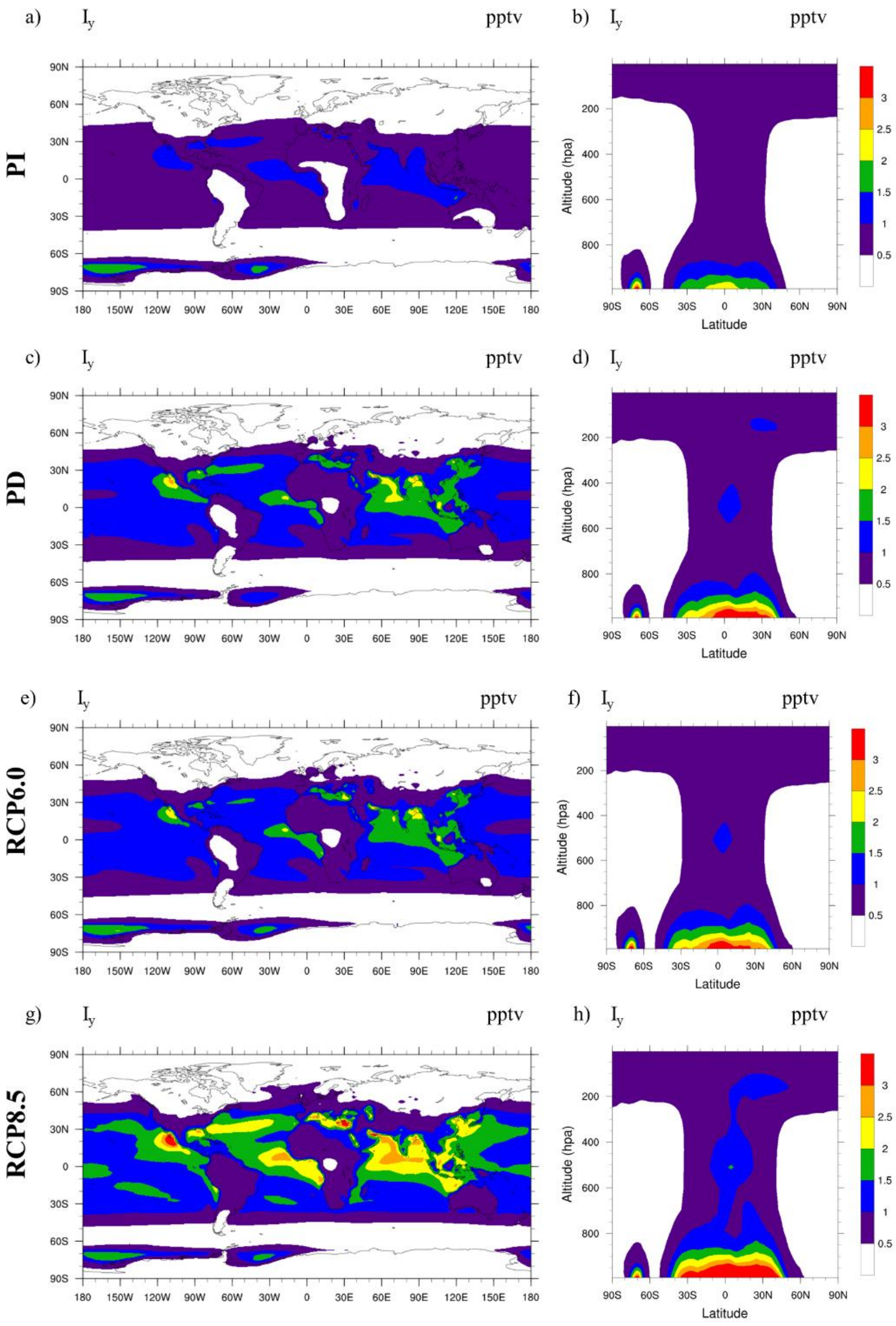


**Supplementary Figure S7. Spatial distribution of inorganic iodine species ($I_y$) for the AANE+ANT simulation.** The annual mean mixing ratio (pptv) vertically averaged within the troposphere (left column) and for the zonal average (middle column) are shown for the pre-industrial case (PI, panels **a, b**), present-day (PD, panels **c, d**) and future RCP 6.0 (panels **e, f**) and RCP 8.5 (panels **g, h**) scenarios. Note that for the NoSLH scenario iodine sources and chemistry are not considered ($I_y$ = 0 pptv). Panels were created using gsn_csm library for NCL.

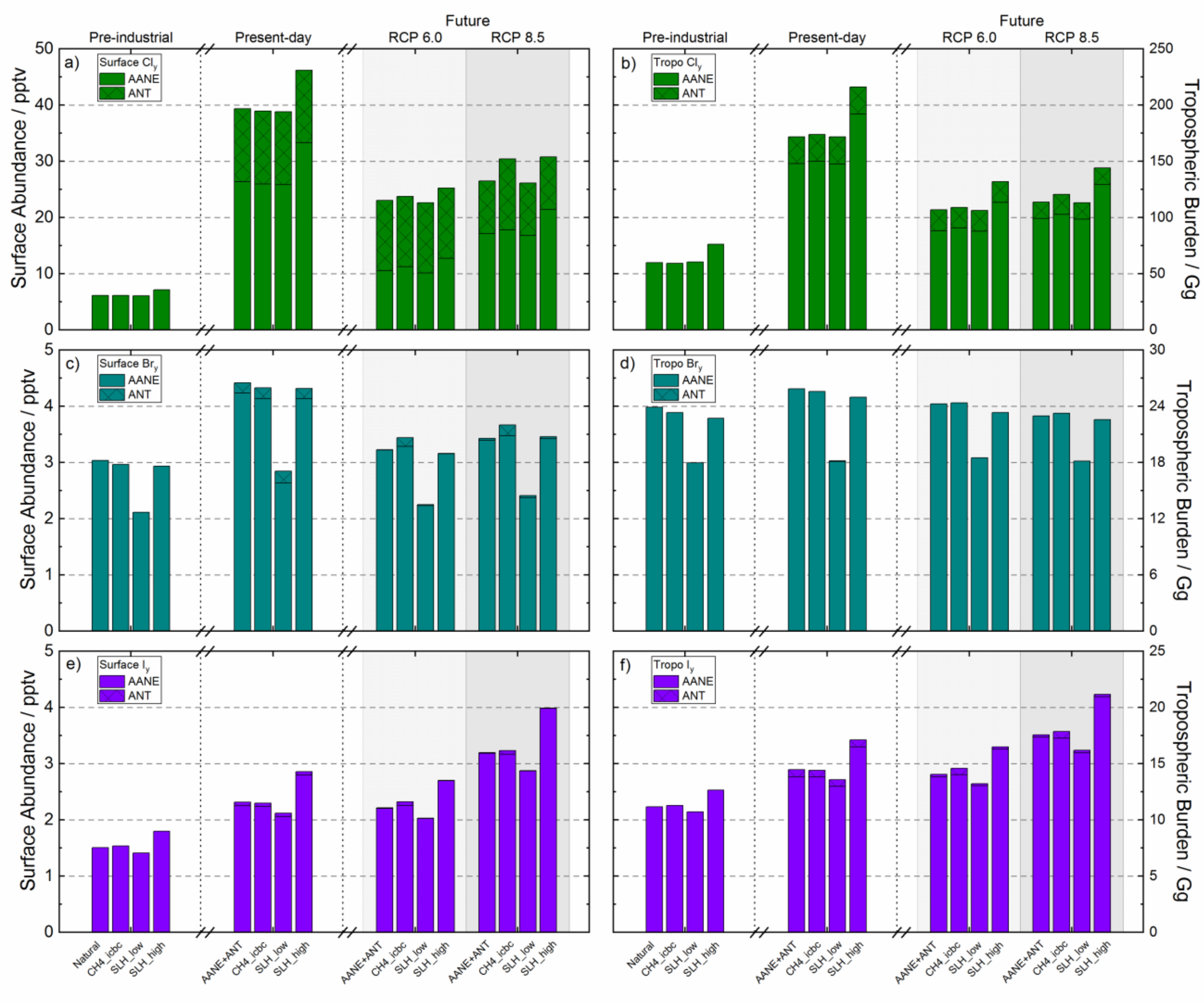


**Supplementary Figure S8. Comparison of global mean SLH abundance for the main sensitivity simulations.** The annual surface abundance (left column) and tropospheric burden (right column) for the case of chlorine (top row, panels **a, b**), bromine (middle row, panels **c, d**) and iodine (bottom row, panels **e, f**) are shown for the only AANE (empty colored bars) and AANE+ANT (stippled colored bars) configurations. Results for the pre-industrial period are on the left of each panel and consider only natural halogen emissions, while the total fluxes in year 2100 for RCP6.0 (light-grey shading) and RCP8.5 (heavy-grey shading) climate scenarios are shown on the right. In all cases, the additional anthropogenic contribution is shown as ANT on top of the values for AANE. Only for the case of the tropospheric bromine burden (panel **d**), AANE+ANT values are slightly smaller than those for AANE, and consequently the stippled dark-cyan bars are not visible on the Figure (see complete dataset in Supplementary Table S5). The spatial and zonal distribution of the SLH abundance for AANE+ANT during each time period is shown in Supplementary Figures S5-S7.

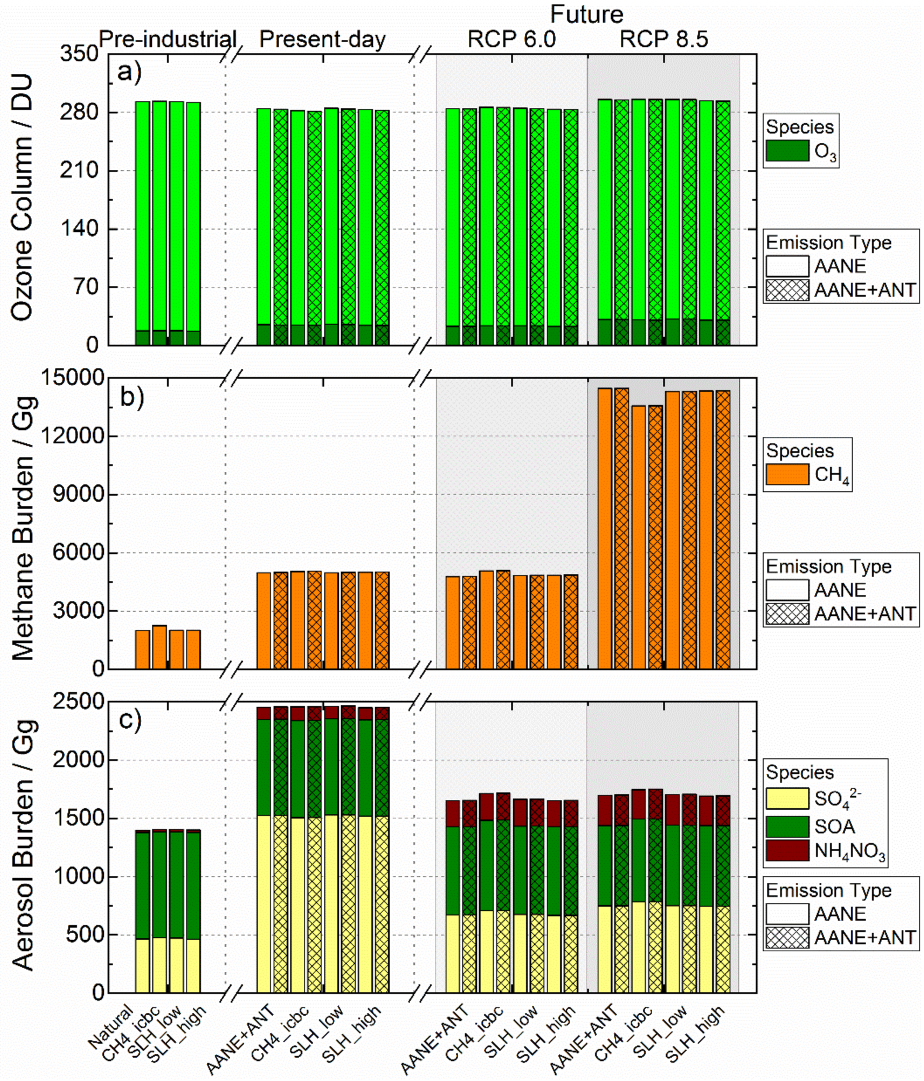


**Supplementary Figure S9. Comparison of global mean SLCF abundance for the main sensitivities during all time periods.** The tropospheric and stratospheric ozone columns are shown in panel **a**, whereas panel **b** presents the total methane burden and panel **c** compiles the tropospheric burden of the main aerosols for AANE (empty colored bars) and AANE+ANT (stippled colored bars) configurations. Results for the pre-industrial period are on the left of each panel and consider only natural halogen emissions, while the total fluxes in year 2100 for RCP6.0 (light-grey shading) and RCP8.5 (heavy-grey shading) climate scenarios are shown on the right.

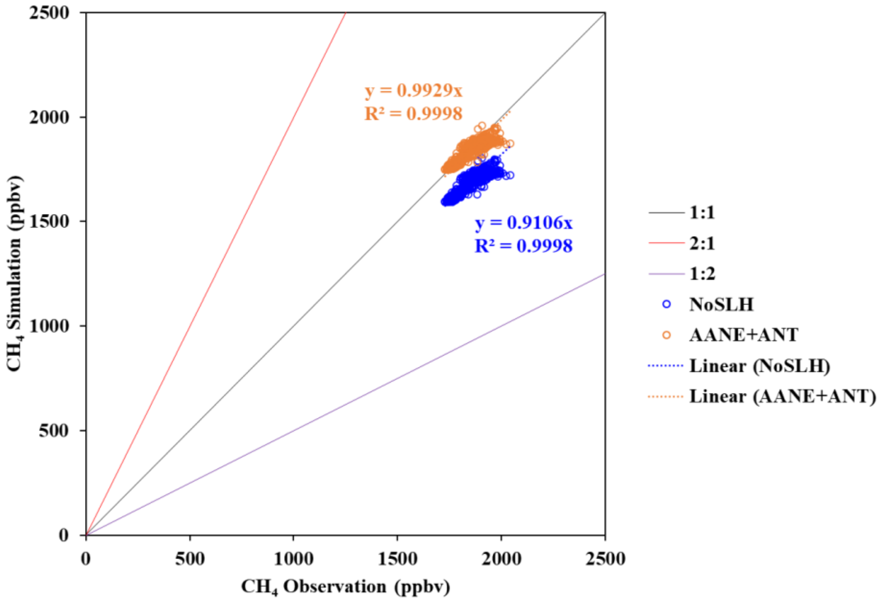


**Supplementary Figure S10. CESM/CAM-Chem validation of methane for present-day conditions.** Correlation of monthly average modelled surface $CH_4$ mixing ratio and observations from the NOAA network[103] are shown in blue for the NoSLH case and in orange for the AANE+ANT configuration.

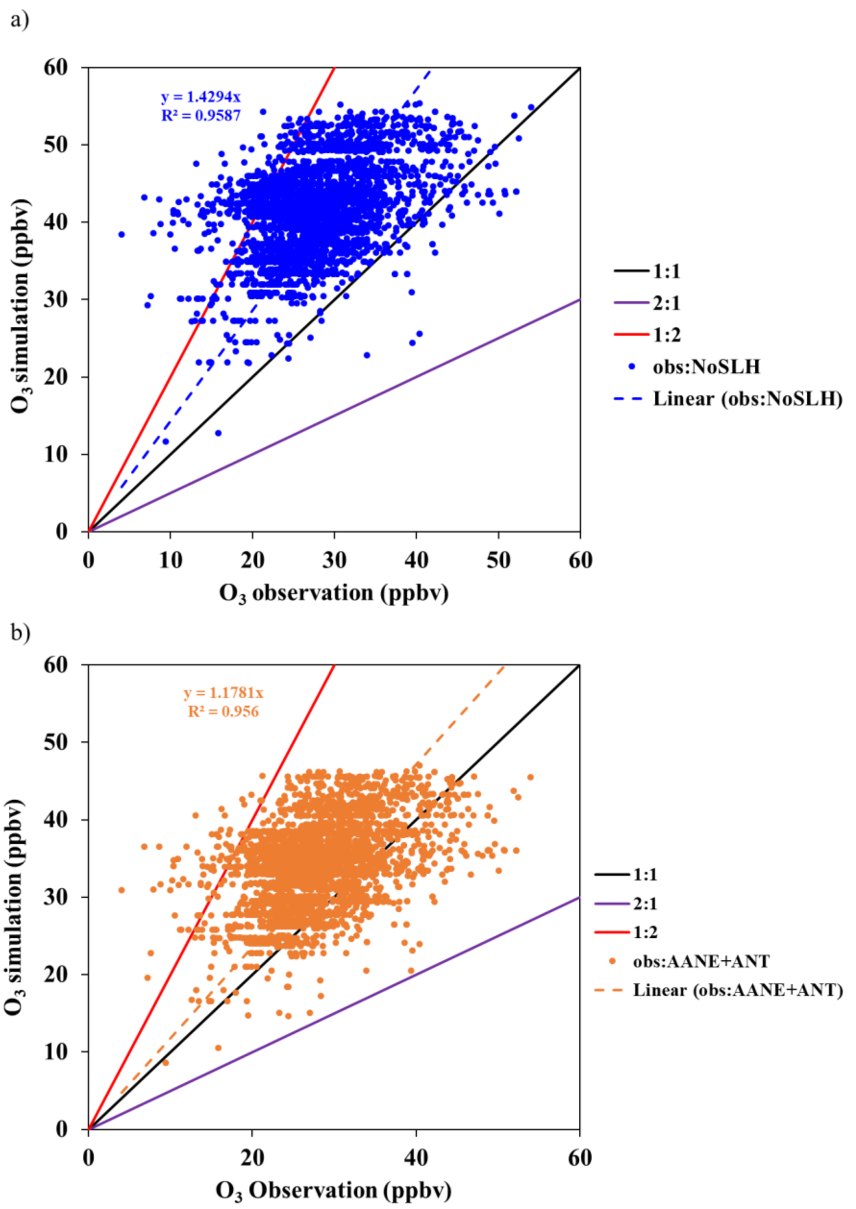


**Supplementary Figure S11. CESM/CAM-Chem validation of ozone for present-day conditions.** Correlation of monthly average modelled surface $O_3$ mixing ratio and observations from the TOAR dataset[104] are shown in blue (panel **a**) for the NoSLH case and in orange (panel **b**) for the AANE+ANT configuration.
